# Integrating Rapid Light- and CO_2_-Response Curves with Low–High–Low Irradiance Kinetics Under Acute Red:Blue Spectra in Leaf Chicory

**DOI:** 10.3390/plants15203194

**Published:** 2026-10-18

**Authors:** Marina Berg Von Linde, Carla Micaela Ripke Ferreira, Ellen Bruna Tacone, Ana Clara Verginio, Elora Berlato Cazela, Bruno Yoshikatsu Suguiura, João Paulo Alves da Silva, Werner Camargos Antunes, José A. M. Demattê, Marcos Rafael Nanni, Renan Falcioni

**Affiliations:** 1 Graduate Program in Agronomy, State University of Maringá, Avenida Colombo, 5790, Maringá 87020-900, Paraná, Brazil; pg407654@uem.br (M.B.V.L.); pg407664@uem.br (C.M.R.F.); pg407665@uem.br (E.B.T.); pg56666@uem.br (A.C.V.); pg407651@uem.br (E.B.C.); pg407650@uem.br (B.Y.S.); ra125202@uem.br (J.P.A.d.S.); wcantunes@uem.br (W.C.A.); mrnanni@uem.br (M.R.N.); 2 Department of Soil Science, Luiz de Queiroz College of Agriculture, University of São Paulo, Avenida Pádua Dias, 11, Piracicaba 13418-260, São Paulo, Brazil; jamdemat@usp.br

**Keywords:** *A*-*C*_i_ response, *A*-PPFD response, *Cichorium intybus* L., chlorophyll fluorescence, dynamic photosynthesis, elevated CO_2_, fluctuating irradiance, stomatal conductance

## Abstract

Rapid response curves and transition kinetics describe complementary dimensions of leaf photosynthesis, yet they are rarely evaluated together under acute spectral contrasts. We quantified net CO_2_ assimilation (*A*), gas exchange and pulse-amplitude-modulated chlorophyll *a* fluorescence in white-light-grown leaf chicory (*Cichorium intybus* L. cv. Pão-de-Açúcar) under R100B0, R90B10, R50B50 and R0B100 chamber conditions, with randomised spectral measurement order. Rapid stepwise assimilation responses to photosynthetic photon flux density (*A*-PPFD) and intercellular CO_2_ (*A*-*C*_i_) were followed by low–high–low irradiance measurements at reference CO_2_ mole fractions (*C*_a_) of 400 and 2000 µmol mol^−1^. R90B10 produced the highest normalised *A*-PPFD area under the curve (8.23 ± 1.04 µmol CO_2_ m^−2^ s^−1^), apparent quantum yield, induction amplitude and integrated carbon gain. R0B100 produced the lowest normalised *A*-*C*_i_ area under the curve (14.45 ± 1.43 µmol CO_2_ m^−2^ s^−1^), empirical CO_2_-response plateau and operational high-*C*_i_ triose-phosphate utilisation (TPU) proxy. At *C*_a_ = 400 µmol mol^−1^, the median time to 90% of the transition amplitude (*t*_90_) was 6.1–12.0 min for *A* and 17.1–24.4 min for stomatal conductance and transpiration. At *C*_a_ = 2000 µmol mol^−1^, *A* approached its final state earlier, with smaller stomatal and transpiration amplitudes under the reported chamber conditions. After the irradiance decreased, *A* reached 90% of its response within the first 60–120 s observation interval, whereas stomatal conductance and transpiration required 11.5–15.6 min to reach the same fraction of their responses, sustaining postillumination water loss. These measurements reveal that acute spectral quality affects the recorded photosynthetic response and carbon–water coordination and that response capacity, amplitude and fractional speed are complementary physiological traits.

## 1. Introduction

Leaves in crop canopies rarely experience constant irradiance. Changes in sunflecks, canopy movement, cloud passage, and programmed lighting can occur faster than coordinated changes in the electron transport rate (ETR), Rubisco activation, metabolite pools, mesophyll CO_2_ transfer, stomatal aperture, and photoprotection. At the physiological steady state, light- and CO_2_-response curves define the operating capacity of these processes across resource gradients, whereas transition measurements determine how rapidly that capacity becomes available after irradiance changes. Both forms of information are therefore required to characterise realised carbon gain under non-steady-state conditions [1,2,3,4,5,6,7].

During a low-to-high-light transition, photochemical and biochemical activation can initially constrain net CO_2_ assimilation (*A*), followed by slower adjustment of stomatal conductance (*g*_sw_). After a light decrease, *A* can decrease before *g*_sw_ and transpiration (*E*), prolonging water loss after the carbon demand has decreased. The relaxation of nonphotochemical quenching (NPQ) can also influence the efficiency of a subsequent increase in irradiance. Response amplitude, threshold times, stabilisation, integrated carbon deficit, and postillumination water loss consequently describe different physiological properties and can vary independently of final high-light values [8,9,10,11,12,13,14,15].

Spectral quality adds an immediate regulatory layer to these transitions. Red and blue photons both support photochemistry, but their absorption within the lamina, excitation distribution, and signalling functions differ. Blue light activates phototropin-dependent guard-cell pathways and influences chloroplast positioning, whereas red-dominant mixtures can provide a large photochemical photon supply. Long-term exposure to contrasting spectra can additionally modify anatomy, pigments, stomatal density, and source–sink relationships; in contrast, acutely changing the actinic spectrum applied to an already developed leaf can affect functional responses before developmental acclimation can occur. Studies in cucumber, tomato, rice, lettuce, wheat, and other crops have shown that the resulting red:blue response is species- and environment-dependent rather than a simple monotonic function of the blue-photon fraction [16,17,18,19,20,21,22,23]. Accordingly, the actinic spectrum imposed during gas exchange measurements should be treated as an experimental factor that may modify the apparent response profile and dynamic behaviour of an already developed leaf rather than as a physiologically neutral background.

Leaf chicory (*Cichorium intybus* L.) is an economically and nutritionally important leafy vegetable, but its photosynthetic response to rapid changes in the actinic spectrum and irradiance remains poorly understood. Existing work has primarily addressed growth under cultivation spectra, steady gas exchange, water relations, or chlorophyll fluorescence under stress [24,25,26,27,28,29]. However, whether the actinic spectrum that supports the greatest photosynthetic capacity also improves carbon gain and carbon–water coordination during rapid irradiance transitions or whether capacity, transition amplitude, and response speed represent partly independent physiological dimensions remain unclear. By integrating rapid *A*-PPFD and *A*-*C*_i_ curves with low–high–low kinetics, the present study explores these relationships in fully developed chicory leaves exposed acutely to contrasting red:blue recipes. An additional unresolved question is whether acute red:blue shifts preserve the coordination between photosystem II (PSII) photochemical regulation and carbon-assimilation performance or transiently uncouple energy conversion from net CO_2_ assimilation. The scientific question is whether greater assimilation in rapid response curves also predicts greater integrated carbon gain during a transition and whether the response magnitude predicts fractional speed or water cost. For supplemental LED lighting in protected cultivation, selecting spectra solely from steady-state responses can overlook the carbon forgone during induction and the water lost while stomata adjust to lower irradiance [8,13,17]. Because blue-light signalling can alter stomatal opening, the red:blue ratio may influence how closely CO_2_ supply and transpirational water loss track photosynthetic demand during these transitions [22,23]. Resolving this coordination provides a physiological basis for evaluating dynamic lighting strategies in terms of carbon gain and leaf water-use efficiency. The experiment does not test a cultivation optimum or yield.

Chlorophyll a fluorescence provides the complementary physiological information required to resolve this coordination because net CO_2_ assimilation is the integrated outcome of stomatal, mesophyll, biochemical, and photochemical processes and therefore does not reveal how absorbed excitation energy is being used. The balance between PSII photochemistry, reaction-centre closure, and regulated energy dissipation varies among species and genotypes because photochemical activation, downstream carbon metabolism, and photoprotection do not respond or relax at identical rates. These differences determine how effectively leaves exploit brief high-light periods, safely dissipate excess excitation, and maintain electron transport coupled with CO_2_ fixation. *F*_v_′/*F*_m_′ describes the efficiency of excitation capture by open PSII reaction centres, whereas Φ_PSII_ represents the operating quantum yield of PSII, and the apparent ETR reflects the electron flux supported by the incident photon supply. NPQ and *q*_N_ describe nonphotochemical quenching through different normalisations, whereas *q*_P_ and *q*_L_ estimate reaction-centre openness under different connectivity assumptions. The joint interpretation of these variables with *A* and the apparent quantum yield of CO_2_ assimilation (Φ_CO2_) can therefore distinguish changes in excitation use and photoprotection from limitations in the conversion of electron transport into net CO_2_ assimilation during acute spectral and irradiance transitions [1,2,4,7,10,19,20,26,27].

Carbon availability may reorganise that coordination. An acute *C*_a_ contrast can therefore serve as a physiological probe, revealing whether spectral differences weaken when the CO_2_ supply is increased or persist after diffusion limitation is partially relieved. Acute CO_2_ enrichment increases substrate availability for carboxylation and can reduce the stomatal aperture required to sustain a given carbon flux. Under fluctuating irradiance, this can accelerate the increase in *A*, alter the relative contributions of diffusive and biochemical limitations, and reduce the water cost of transient stomatal opening. These minute-scale responses are distinct from acclimation to chronically elevated CO_2_, which can modify nitrogen allocation, stomatal development, and source–sink balance [30,31,32,33,34].

We therefore evaluated four acute red:blue recipes in leaf chicory using two linked measurement frameworks: (i) rapid stepwise *A*-PPFD and *A*-*C*_i_ curves to quantify operational light- and carbon-response profiles and (ii) low–high–low kinetics at *C*_a_ = 400 and 2000 µmol mol^−1^ to quantify induction, relaxation, stomatal and transpiration timing, transition costs, photochemical regulation, and multivariate coordination. Simultaneous chlorophyll fluorescence further linked carbon exchange to PSII operating efficiency, electron transport, reaction-centre openness, and regulated energy dissipation. We hypothesised that a red-dominant mixture containing a small blue component would produce greater integrated *A*-PPFD and *A*-*C*_i_ responses than blue-only light, because red photons can support carbon assimilation more efficiently than blue photons, while a small blue component can activate guard-cell signalling [19,22]. We further hypothesised that capacity ranking would not necessarily predict transition speed because biochemical activation and stomatal movement impose time-dependent constraints that are not captured by response magnitude [7,8,17]. Finally, we hypothesised that acute CO_2_ enrichment would accelerate the fractional induction of *A* while reducing the stomatal and transpiration adjustments required during irradiance transitions, because greater CO_2_ availability can relieve diffusional limitation and sustain carboxylation at lower stomatal conductance [23,30]. The spectral measurement order was randomised, and the hypotheses were evaluated through plant-level response profiles and kinetic descriptors.

## 2. Results

### 2.1. Spectral Effects on Complete Light and CO_2_-Response Curves

The normalised *A*-PPFD area under the curve (AUC_norm_) differed among the acute spectra when the plants were treated as repeated blocks (Greenhouse–Geisser *F*(1.75, 12.23) = 4.092; *p* = 0.0479; partial η_p_^2^ = 0.369; Friedman *p* = 0.0045). The means ± SEMs were 6.92 ± 1.35 for R100B0, 8.23 ± 1.04 for R90B10, 7.37 ± 0.98 for R50B50, and 6.14 ± 0.74 µmol CO_2_ m^−2^ s^−1^ for R0B100 (*n* = 8). Holm-adjusted comparisons revealed a stronger response under R90B10 than under R0B100 (paired difference = 2.09, *p* = 0.0210); no other pair met the adjusted criterion. Thus, R90B10 and R0B100 had the highest and lowest *A*-PPFD AUC_norm_ values, respectively.

Spectral separation was stronger for *A*-*C*_i_ AUC_norm_ (Greenhouse–Geisser *F*(1.28, 8.96) = 208.945; *p* < 0.001; partial η_p_^2^ = 0.968; Friedman *p* < 0.001). The means were 21.97 ± 1.71 for R100B0, 22.13 ± 1.64 for R90B10, 19.55 ± 1.57 for R50B50, and 14.45 ± 1.43 µmol CO_2_ m^−2^ s^−1^ for R0B100 (*n* = 8). All the Holm-adjusted pairs differed (*p* < 0.001) except R100B0 versus R90B10 (*p* = 0.350). Empirical *A*_max,CO2_ followed the same pattern: 24.97 ± 1.86, 24.74 ± 1.77, 21.84 ± 1.63, and 16.17 ± 1.36 µmol CO_2_ m^−2^ s^−1^ under R100B0, R90B10, R50B50, and R0B100, respectively (*n* = 8 per spectrum). The plant-blocked spectral contrast was large (HC3 *F*(3, 21) = 95.294, *p* < 0.001, partial η_p_^2^ = 0.967), and every Holm-adjusted contrast differed except R100B0 versus R90B10.

The primary analyses used complete *A*–PPFD and *A*–*C*_i_ curves, including the high-*C*_i_ observations. Complementary comparisons based on recorded analyser classifications preserved the mean spectral ordering over shared measured domains, with lower precision for some contrasts. These comparisons concern the domains represented by each classification subset; the full curves remain the basis for the empirical plateaux and FvCB estimates (Supplementary Sections S1–S12, Tables S1–S17).

**Figure 1:**
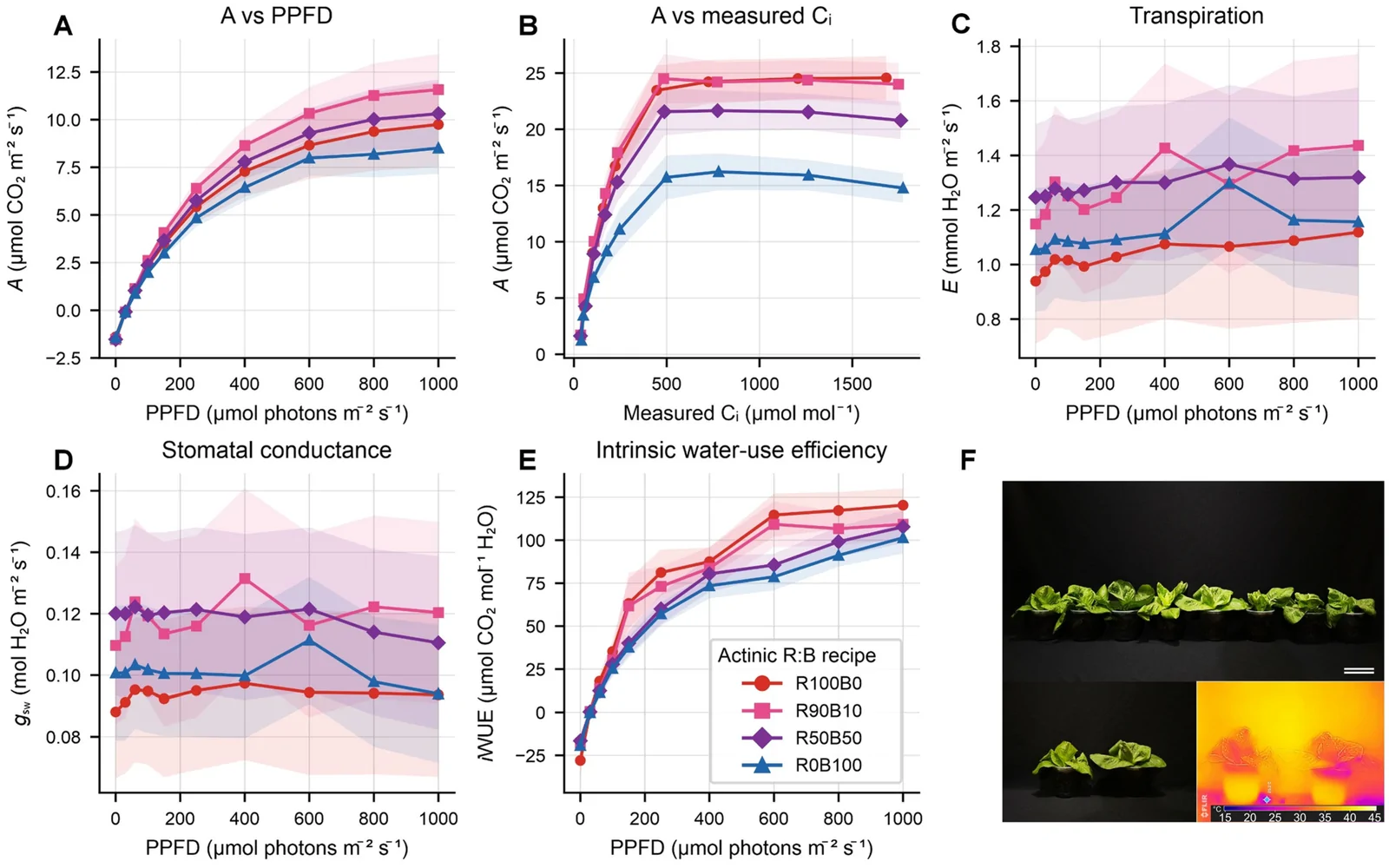
Rapid stepwise gas exchange under four acute red:blue photon recipes. (**A**) *A* versus PPFD; (**B**) *A* versus measured *C*_i_; (**C**) *E* versus PPFD; (**D**) *g*_sw_ versus PPFD; (**E**) *i*WUE versus PPFD; and (**F**) representative visible and radiometric thermal views of plants after 8 h of the common growth photoperiod. Points and bands are the means ± SEMs (*n* = 8 plants per recipe). The *A*-PPFD curves contained ten ordered PPFD levels, and the *A*-*C*_i_ curves contained nine ordered *C*_a_ levels. Scale bar = 10 cm.

Across the individual PPFD steps, *E*, *g*_sw_, and *i*WUE followed spectrum-specific trajectories, with the clearest separation occurring at intermediate and high irradiance (Figure 1C–E). The ordering of the gas exchange responses was not identical among the variables, indicating that differences across spectral conditions in terms of carbon gain were accompanied by, but were not reducible to, changes in stomatal water flux. The visible and radiometric thermal images in Figure 1F were acquired after 8 h of the common growth photoperiod. Thus, Figure 1F shows the representative canopy temperature pattern under the shared growth environment and does not represent a separate spectral growth treatment.

### 2.2. Fluorescence, Thermal Responses, and Curve-Derived Parameters

**Figure 2:**
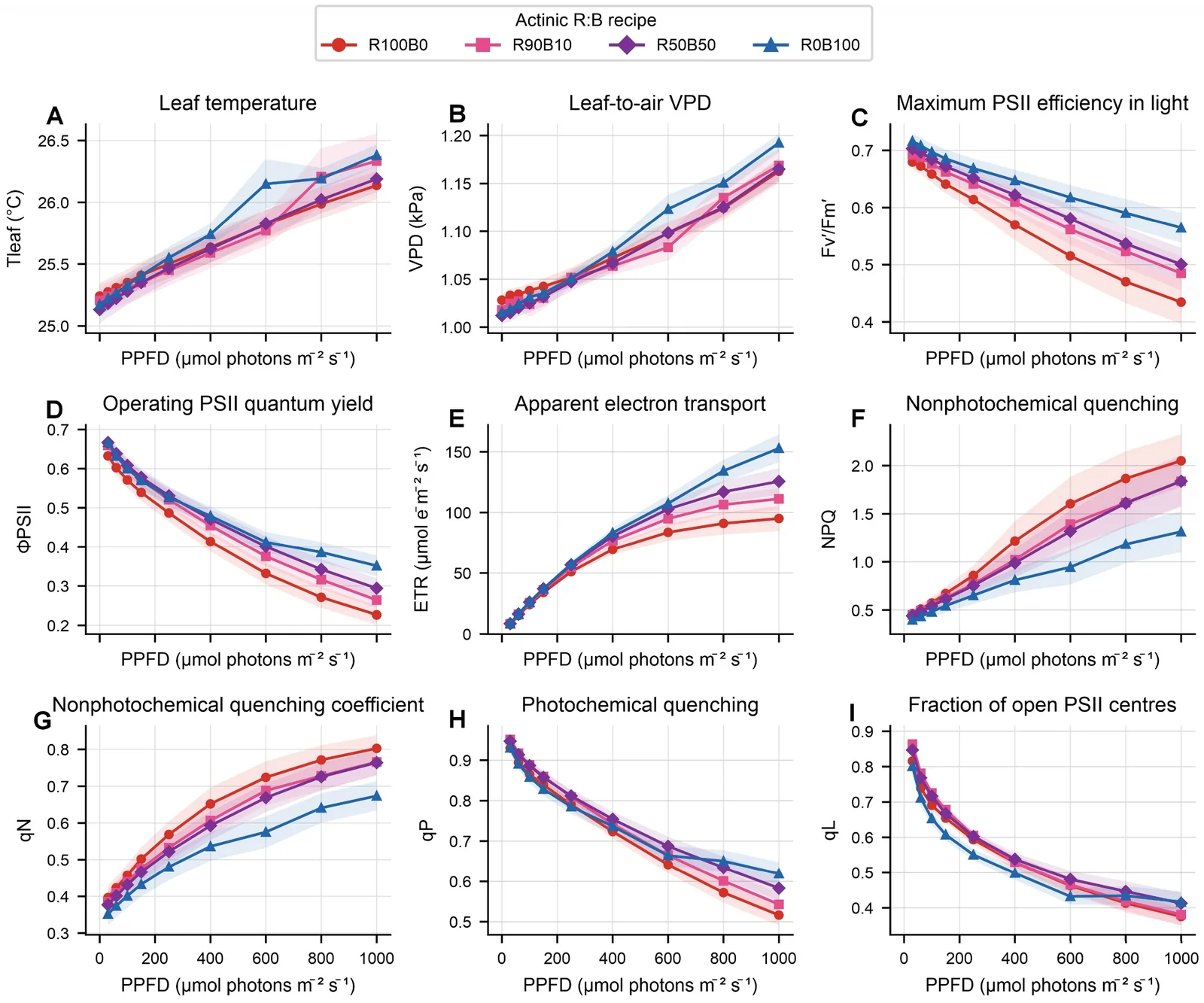
*T*_leaf_, VPD_leaf_, and chlorophyll-fluorescence responses during rapid stepwise *A*-PPFD curves. Panels show (**A**) *T*_leaf_, (**B**) VPD_leaf_, (**C**) *F*_v_′/*F*_m_′, (**D**) Φ_PSII_, (**E**) ETR, (**F**) NPQ, (**G**) *q*_N_, (**H**) *q*_P_, and (**I**) *q*_L_. The values are the means ± SEMs (*n* = 8 plants per recipe).

The stepwise fluorescence profiles changed consistently across the PPFD gradient and showed spectrum-specific trajectories for *F*_v_′/*F*_m_′, Φ_PSII_, ETR, NPQ, *q*_N_, *q*_P_, and *q*_L_ (Figure 2). Increasing the PPFD decreased the *F*_v_′/*F*_m_′ and Φ_PSII_, indicating a lower efficiency of excitation capture by open PSII reaction centres and a lower realised PSII quantum yield, respectively. The apparent ETR nevertheless increased because the greater incident photon flux offset the decline in quantum efficiency. NPQ and *q*_N_ increased, whereas *q*_P_ and *q*_L_ decreased, indicating stronger nonphotochemical quenching and a lower fraction of open PSII reaction centres under their respective connectivity models. *T*_leaf_ and VPD_leaf_ also diverged at high PPFD, with R0B100 reaching the highest values. The stepwise fluorescence profiles in Figure 2 are presented descriptively; formal treatment inference for fluorescence dynamics was based on the induction and relaxation baseline-corrected area under the curve (AUC_bc_) analyses reported in Section 2.5.

The apparent quantum yield (*α*) differed among the spectra (HC3 *F*(3, 21) = 4.825, *p* = 0.0104, partial η_p_^2^ = 0.600). R90B10 (0.04559 ± 0.00217) exceeded R0B100 (0.03794 ± 0.00268) after the Holm adjustment (*p* = 0.0284). The plant-blocked spectrum tests were not significant for *A*_p,max_ (*p* = 0.0954), *A*_max_ (*p* = 0.1065), *R*_d,light_ (*p* = 0.0575), the light compensation point (*p* = 0.1556), the PPFD at 75% modelled saturation (*p* = 0.8842), or *g*_sw_ at a PPFD of 400 µmol photons m^−2^ s^−1^ (*p* = 0.1951).

Among the empirical *A*-*C*_i_ descriptors, *A*_max,CO2_ and carboxylation efficiency (CE) differed among the spectra, whereas Γ_app_ did not. The CE was 0.13684 ± 0.01454, 0.14797 ± 0.01595, 0.12649 ± 0.01428, and 0.08866 ± 0.01435 mol m^−2^ s^−1^ in the common treatment order (HC3 *F*(3, 21) = 16.112, *p* < 0.001, partial η_p_^2^ = 0.816). R0B100 was lower than R100B0, R90B10, and R50B50 after the Holm adjustment, and R90B10 exceeded R50B50. Γ_app_ ranged from 11.33 to 20.40 µmol CO_2_ mol^−1^ and did not differ among the spectra (HC3 *F*(3, 21) = 1.047, *p* = 0.3926). Γ_app_ is an extrapolated empirical zero crossing, not an independently measured biochemical compensation point.

**Figure 3:**
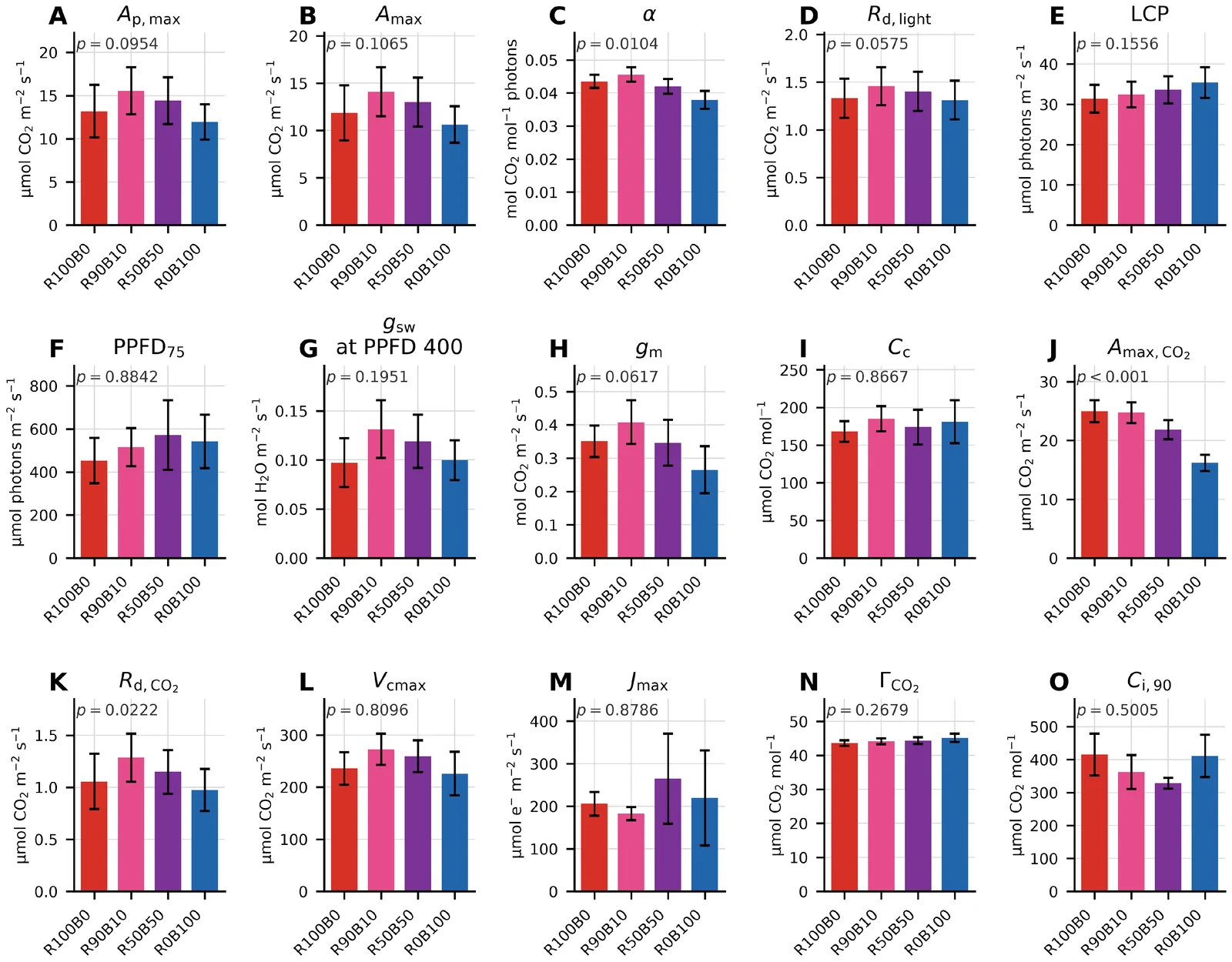
Physiological descriptors derived from rapid stepwise *A*-PPFD and *A*-*C*_i_ curves. (**A**) *A*_p,max_; (**B**) *A*_max_; (**C**) *α*; (**D**) *R*_d,light_; (**E**) light compensation point; (**F**) PPFD_75_, the PPFD reaching 75% of the response amplitude above *A*(*Q* = 0); (**G**) *g*_sw_ at a PPFD of 400 µmol photons m^−2^ s^−1^; (**H**) *g*_m_; (**I**) *C*_c_; (**J**) empirical *A*_max,CO2_; (**K**) *R*_d,CO2_, supplied by the paired Yin light-response estimate and fixed in the finite-*g*_m_ CO_2_ model; (**L**) *V*_cmax_; (**M**) *J*_max_ under the stated *J*-PPFD relationship; (**N**) Γ_CO2_, the model-derived net compensation point; and (**O**) *C*_i,90_, the *C*_i_ reaching 90% of the modelled response amplitude. The bars are the means ± SEMs from eight plant-level estimates per spectrum, except *g*_m_ under R100B0 (*n* = 7). Panel *p* values are the corresponding plant-blocked HC3 comparisons in Table 1. For panels H, I and *L*–O, these are secondary descriptive comparisons of fitted point estimates: they do not propagate within-curve estimation uncertainty or establish biochemical spectral differences or equivalence. SEM represents between-plant variation; parameter uncertainty and sensitivity to fitting assumptions are assessed separately in Supplementary Sections S5, S8, S9 and S11.

Among the curve-derived endpoints, the respiration parameter used in the finite-*g*_m_ Farquhar–von Caemmerer–Berry (FvCB) CO_2_ model (*R*_d,CO2_) differed among the spectra (HC3 *F*(3, 21) = 3.953, *p* = 0.0222, partial η_p_^2^ = 0.427). R90B10 and R50B50 exceeded R0B100 after the Holm adjustment. This parameter was supplied by the paired Yin light-response estimate and fixed during the finite-*g*_m_ fit. The operational high-*C*_i_ TPU proxy (TPU_op_) also showed a strong spectrum effect (HC3 *F*(3, 21) = 86.640, *p* < 0.001, partial η_p_^2^ = 0.963). The means were 8.53 ± 0.67, 8.51 ± 0.63, 7.49 ± 0.57 and 5.58 ± 0.47 µmol m^−2^ s^−1^ in the common treatment order, expressed as triose-phosphate equivalents. R100B0 and R90B10 did not differ in the primary calculation, whereas the remaining Holm-adjusted contrasts were significant. The fitted *g*_m_ (*p* = 0.0617), *C*_c_ (*p* = 0.8667), *V*_cmax_ (*p* = 0.8096), *J*_max_ (*p* = 0.8786), Γ_CO2_ (*p* = 0.2679) and *C*_i,90_ (*p* = 0.5005) yielded nonsignificant nominal comparisons of coefficients. These secondary comparisons describe the estimates under a common fitting convention; they are not used to infer either spectral differences or equivalence in independently identified biochemical or diffusional capacities. The tests for all the endpoints displayed in Figure 3 are reported in Table 1, with uncertainty and sensitivity analyses in Supplementary Section S5.

The upper-bound estimates influenced the *J*_max_ summaries. The 30-curve sensitivity gave R50B50 and R0B100 means of 159.59 and 108.00 µmol e^−^ m^−2^ s^−1^, versus 264.64 and 219.50 in the complete dataset. The balanced 28-curve sensitivity, comprising plants 1–6 and 8 across all spectra, gave a nominal coefficient *p* value of 0.0033 versus 0.8786 for all 32 curves. These comparisons quantify sensitivity to the two upper-bound estimates from plant 7; all original curves and primary results are retained. The fitted *J*_max_ comparisons therefore cannot establish a stable biochemical ranking (Supplementary Section S11, Table S13).

### 2.3. Gas-Exchange Induction Under Acute Spectral and CO_2_ Contrasts

**Figure 4:**
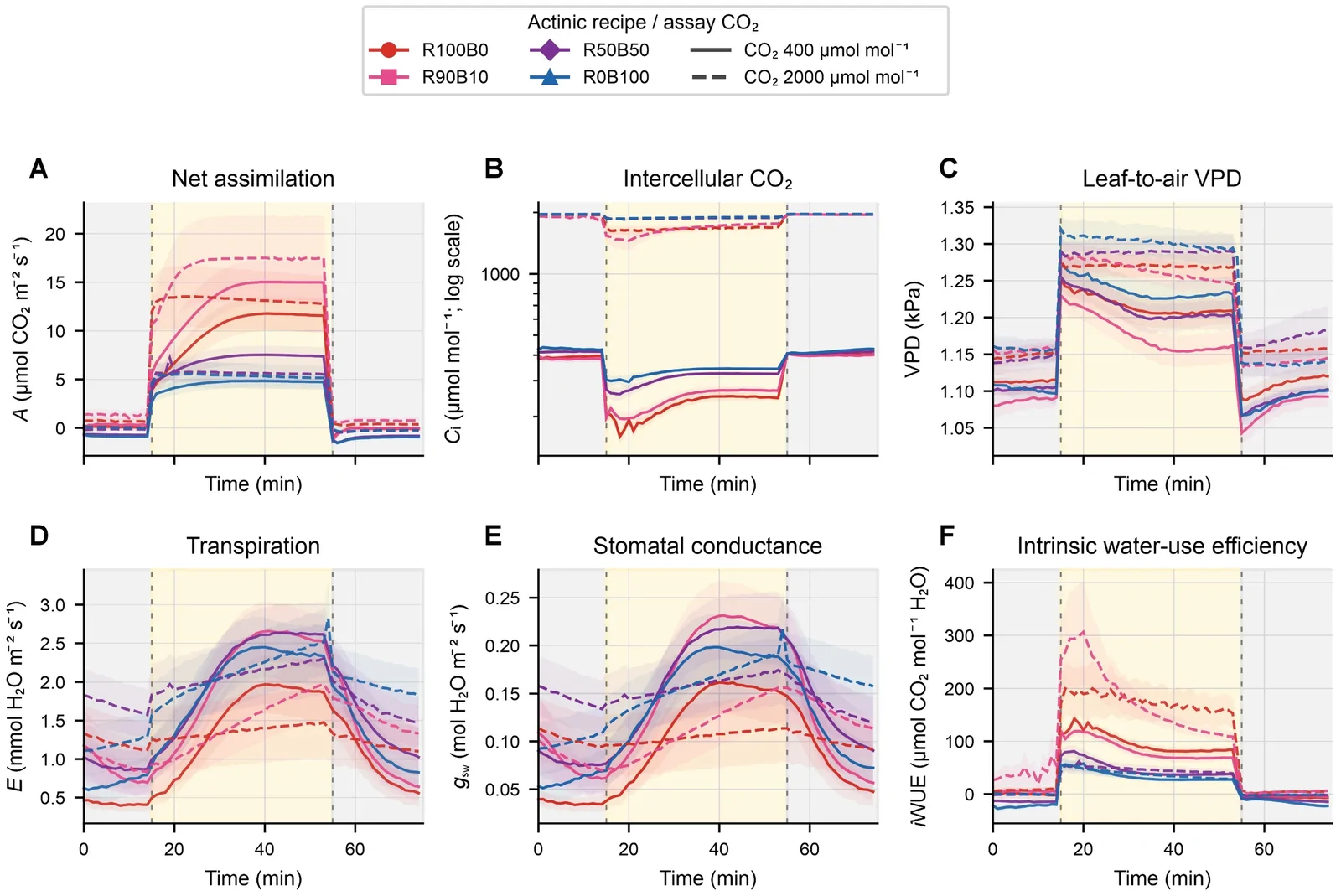
Gas exchange kinetics during the 75 min low–high–low protocol. (**A**) *A*; (**B**) *C*_i_ on a logarithmic axis; (**C**) VPD_leaf_; (**D**) *E*; (**E**) *g*_sw_; and (**F**) *i*WUE. Lines and bands are the means ± SEMs for four acute red:blue recipes; line type identifies *C*_a_ = 400 or 2000 µmol mol^−1^. Each recipe × *C*_a_ combination contained *n* = 8 plant series; pointwise, *n* = 7–8. Vertical boundaries mark changes in the PPFD from 50 to 1000 and back to 50 µmol photons m^−2^ s^−1^.

Compared with the rapid *A*-PPFD curve means, the low-to-high-light transition resolved larger spectral differences (Figure 4). The assimilation AUC_bc_ was greatest under R90B10 at both *C*_a_ concentrations: 494.94 at 400 and 593.86 µmol CO_2_ m^−2^ s^−1^ min at *C*_a_ = 2000 µmol mol^−1^. R100B0 ranked second (385.91 and 482.58), R50B50 was 296.49 and 224.11, and R0B100 was 210.46 and 208.52 µmol CO_2_ m^−2^ s^−1^ min, respectively. The spectral conditions differed strongly in the AUC_bc_ of *A* (*F*(1.30, 9.08) = 26.646, *p* < 0.001, *q* < 0.001, partial η_p_^2^ = 0.792), whereas the *C*_a_ contrast and spectrum × *C*_a_ interaction did not meet the prespecified threshold.

The largest differences between *C*_a_ conditions occurred in stomatal conductance and transpiration. At *C*_a_ = 400 µmol mol^−1^, the spectrum-specific mean induction amplitudes ranged from 0.119 to 0.158 mol H_2_O m^−2^ s^−1^ for *g*_sw_ and from 1.469–1.833 mmol H_2_O m^−2^ s^−1^ for *E*; at *C*_a_ = 2000 µmol mol^−1^, the corresponding ranges were 0.017–0.080 mol H_2_O m^−2^ s^−1^ and 0.334–1.186 mmol H_2_O m^−2^ s^−1^, respectively. Consistent with these smaller adjustments, *g*_sw_ AUC_bc_ decreased from 3.35–4.45 at *C*_a_ = 400 to 0.35–2.01 mol H_2_O m^−2^ s^−1^ min at *C*_a_ = 2000 (*F*(1, 7) = 17.711, *p* = 0.004, *q* = 0.020, partial η_p_^2^ = 0.717). *E* showed the same *C*_a_ pattern (*F*(1, 7) = 10.394, *p* = 0.015, *q* = 0.036, partial η_p_^2^ = 0.598). The spectral contrast on *E* was nominally significant (*p* = 0.046) but did not meet the family-adjusted criterion (*q* = 0.057).

The spectral conditions differed in the signed induction AUC_bc_ of *i*WUE (*F*(1.64, 11.50) = 37.698, *p* < 0.001, *q* < 0.001, partial η_p_^2^ = 0.843) and *C*_i_ (*F*(1.71, 11.97) = 38.351, *p* < 0.001, *q* < 0.001, partial η_p_^2^ = 0.846). The spectrum × *C*_a_ tests for *C*_i_ and *i*WUE had nominal *p* values of 0.025 and 0.032, respectively, but both yielded *q* = 0.081 and therefore did not meet the family-adjusted criterion. Nevertheless, at *C*_a_ = 2000 µmol mol^−1^, the mean signed *i*WUE AUC_bc_ values were 6429.82 for R100B0, 4748.12 for R90B10, 1832.50 for R50B50, and 1508.48 µmol CO_2_ mol^−1^ H_2_O min for R0B100.

### 2.4. Response Amplitudes, Response Times, Stabilisation, and Transition Costs

At *C*_a_ = 400 µmol mol^−1^, the increase in *A* ranged from 5.63 ± 0.62 µmol CO_2_ m^−2^ s^−1^ under R0B100 to 14.68 ± 1.99 µmol CO_2_ m^−2^ s^−1^ under R90B10 (Table 2). The median *A t*_90_ ranged from 6.1 min under R0B100 to 12.0 min under R100B0, whereas *g*_sw_ and *E* ranged from 17.5–24.4 and 17.1–24.0 min, respectively. The final high-light *g*_sw_ means ranged from 0.154 to 0.220 mol H_2_O m^−2^ s^−1^, and the final *E* means ranged from 1.877 to 2.617 mmol H_2_O m^−2^ s^−1^. Sustained stabilisation accounted for 68.9–76.9% of the observed induction span for *g*_sw_ and 67.6–75.6% for *E*. R90B10 therefore combined the greatest carbon response with a relatively long approach to its greater final state and the greatest absolute *C*_def_.

At *C*_a_ = 2000 µmol mol^−1^, the increase in *A* ranged from 5.21–16.19 µmol CO_2_ m^−2^ s^−1^, and the final high-light *A* ranged from 5.19 to 17.50 µmol CO_2_ m^−2^ s^−1^. The median *A t*_90_ occurred by the first measurement under R100B0, R50B50, and R0B100 and was 3.1 min under R90B10. In contrast, the median *t*_90_ ranged from 28.7–32.1 min for *g*_sw_ and from 27.5–31.8 min for *E*. *C*_def_ ranged from 0.059 to 1.896 mmol CO_2_ m^−2^ at *C*_a_ = 2000 µmol mol^−1^ and from 0.575 to 4.639 mmol CO_2_ m^−2^ at *C*_a_ = 400 µmol mol^−1^. It varied with spectrum (Greenhouse‒Geisser *F*(1.51, 10.54) = 10.889, *p* = 0.0042, *q* = 0.0084, partial η_p_^2^ = 0.609), *C*_a_ (*F*(1, 7) = 16.683, *p* = 0.0047, *q* = 0.0047, partial η_p_^2^ = 0.704), and their interaction (Greenhouse‒Geisser *F*(1.88, 13.18) = 5.141, *p* = 0.0238, *q* = 0.0475, partial η_p_^2^ = 0.423).

After the PPFD decreased from 1000 to 50 µmol photons m^−2^ s^−1^, the decline in *A* was 5.68–15.00 µmol CO_2_ m^−2^ s^−1^ at *C*_a_ = 400 and 5.42–16.72 µmol CO_2_ m^−2^ s^−1^ at *C*_a_ = 2000. The median *A t*_90_ occurred by the first poststep measurement for every treatment, whereas *g*_sw_ and *E* ranged from 11.6–15.6 and 11.5–14.5 min, respectively. *W*_exc_ ranged from 449–560 mmol H_2_O m^−2^ at *C*_a_ = 400 and 109–231 mmol H_2_O m^−2^ at *C*_a_ = 2000. *C*_a_ conditions differed in *W*_exc_ (*F*(1, 7) = 30.193, *p* < 0.001, *q* = 0.0018, partial η_p_^2^ = 0.812), whereas spectrum (*p* = *q* = 0.284) and spectrum × *C*_a_ (*p* = *q* = 0.694) did not. The downstep thus revealed a consistent temporal mismatch between the rapid decline in carbon assimilation and the slower decline in stomatal water loss.

Across spectra and *C*_a_ levels, the conservative first-order reporting criterion retained induction models for the 5/64 *A*, 44/64 *g*_sw_, and 36/64 *E* trajectories. The median [Q1–Q3] *L* and *τ* were 1.71 [0.50–1.93] and 8.48 [7.97–9.44] min for *A*, 4.73 [2.79–6.87] and 10.95 [9.37–12.81] min for *g*_sw_, and 3.44 [2.55–5.42] and 11.46 [9.62–12.70] min for *E*, respectively. During relaxation, no *A* trajectory met the criterion because the response reached the destination range before sufficient intermediate observations were recorded. Models were retained for 47/64 *g*_sw_ and 24/64 *E* trajectories; *L* and *τ* were 1.43 [1.09–2.43] and 5.18 [4.58–6.18] min for *g*_sw_ and 0.65 [0.25–0.77] and 5.54 [4.43–6.34] min for *E*, respectively. These model-dependent estimates support the slower stomatal-water adjustment but were not used as primary treatment tests.

### 2.5. Photochemical, Photoprotective, and Thermal Dynamics

**Figure 5:**
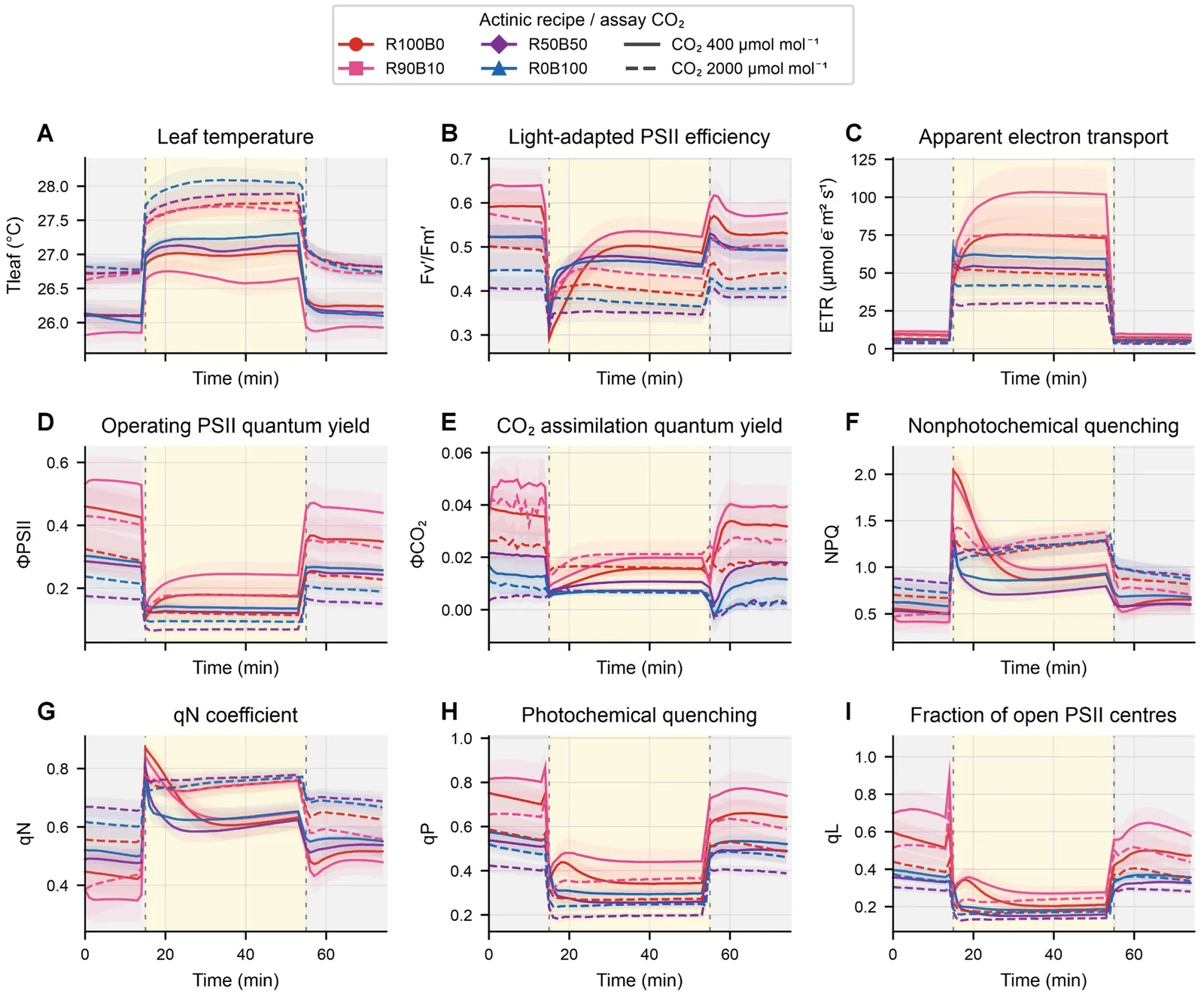
Leaf-temperature and fluorescence kinetics during the low–high–low protocol. (**A**) *T*_leaf_; (**B**) *F*_v_′/*F*_m_′; (**C**) ETR; (**D**) Φ_PSII_; (**E**) Φ_CO2_; (**F**) NPQ; (**G**) *q*_N_; (**H**) *q*_P_; and (**I**) *q*_L_. Lines and bands are the means ± SEMs; the line type indicates *C*_a_ = 400 or 2000 µmol mol^−1^. Each recipe × *C*_a_ combination contained *n* = 8 plant series; pointwise, *n* = 7–8.

During induction (Figure 5), the spectrum affected the AUC_bc_ of Φ_PSII_ (*F*(1.50, 10.51) = 35.857; *p* < 0.001; *q* < 0.001; partial η_p_^2^ = 0.837) and NPQ (*F*(1.28, 8.94) = 30.817; *p* < 0.001; *q* < 0.001; partial η_p_^2^ = 0.815). Under R90B10, Φ_PSII_ decreased from 0.532 to 0.243 at *C*_a_ = 400 and from 0.404 to 0.177 at *C*_a_ = 2000, whereas ETR increased from 11.2 to 102.2 and from 8.5 to 74.7 µmol e^−^ m^−2^ s^−1^, respectively. NPQ increased from 0.409 to 1.010 at *C*_a_ = 400 and from 0.508 to 1.362 at *C*_a_ = 2000. Similarly, R90B10 produced the greatest negative Φ_PSII_ AUC_bc_ (−11.51 and −8.81) and the greatest positive NPQ AUC_bc_ (28.23 and 30.72, respectively).

The spectral conditions also differed in *T*_leaf_ (*F*(1.91, 13.35) = 51.862, *p* < 0.001, *q* < 0.001, partial η_p_^2^ = 0.881) and VPD_leaf_ (*F*(1.76, 12.32) = 18.400, *p* < 0.001, *q* < 0.001, partial η_p_^2^ = 0.724) during induction. R0B100 showed the greatest thermal excursion, with *T*_leaf_ increasing by approximately 1.29 °C at *C*_a_ = 400 and 1.27 °C at *C*_a_ = 2000 between the initial low-light and final high-light states. Differences among spectral conditions also met the family-adjusted criterion for *F*_v_′/*F*_m_′, ETR, Φ_CO2_, *q*_N_, *q*_P_, and *q*_L_ (Table 3), describing concurrent changes in excitation use, regulated dissipation, reaction-centre openness, and leaf energy balance.

After the PPFD returned to 50 µmol photons m^−2^ s^−1^, the spectrum affected the AUC_bc_ of *A* (*F*(1.24, 8.70) = 32.943, *q* < 0.001), *C*_i_ (*F*(1.61, 11.26) = 54.844, *q* < 0.001), and *i*WUE (*F*(1.48, 10.34) = 52.635, *q* < 0.001). The *C*_i_ and *i*WUE interactions yielded *q* = 0.087 and did not meet the adjusted criterion. *C*_a_ conditions differed strongly in postillumination *g*_sw_ (*F*(1, 7) = 51.774, *q* < 0.001, partial η_p_^2^ = 0.881) and *E* (*F*(1, 7) = 27.211, *q* = 0.003, partial η_p_^2^ = 0.795). The differences among the spectral conditions persisted across all the photochemical and quenching variables during relaxation; the effect of Ca on NPQ was *p* = 0.045, but *q* = 0.182.

### 2.6. Multivariate Organisation of Dynamic and Curve Traits

**Figure 6:**
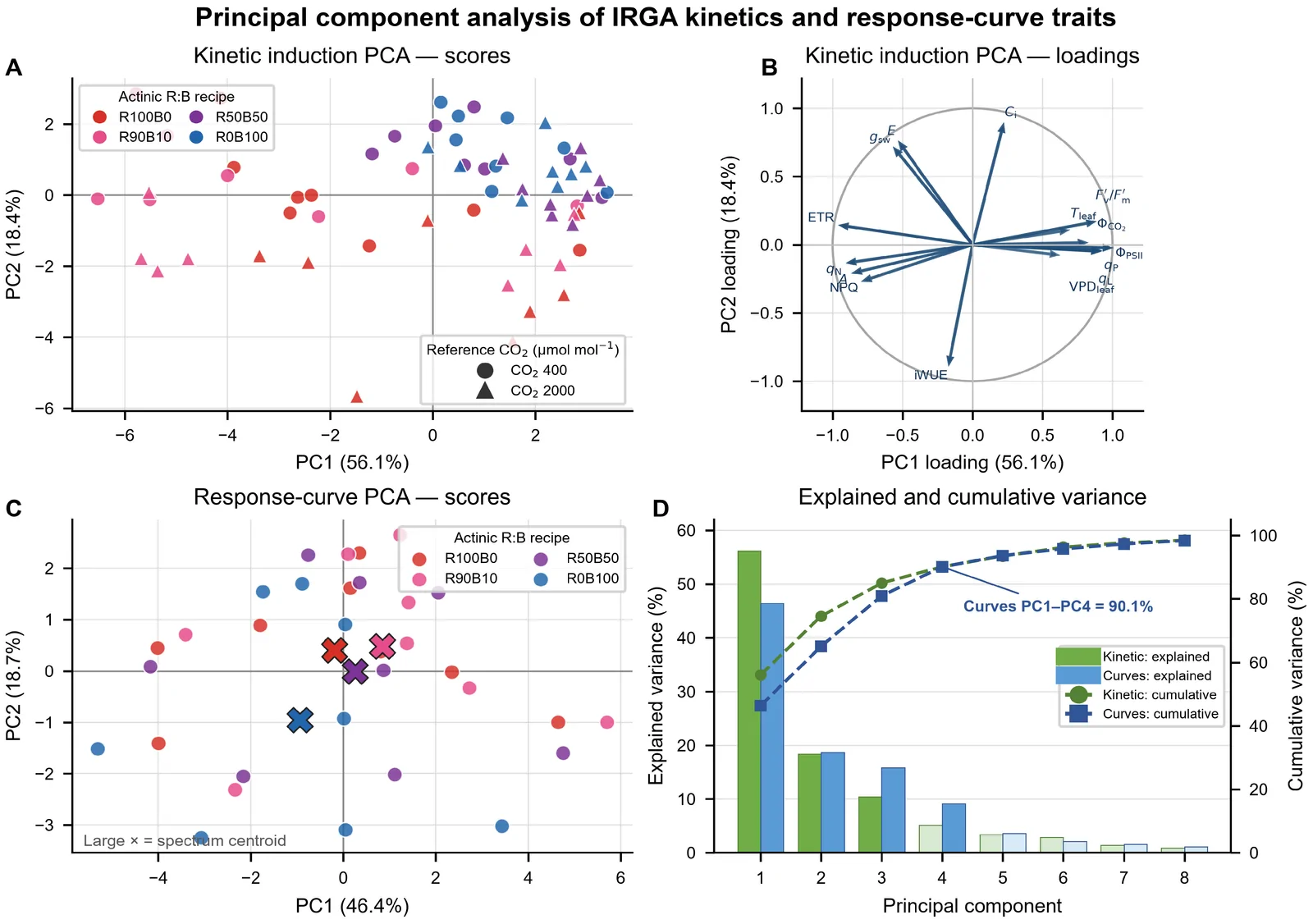
Principal component analyses of induction kinetics and response curve parameters. (**A**) Kinetic PCA scores from 15 signed-induction AUC_bc_ descriptors across 64 plants × spectrum × *C*_a_ series (8 plants × 4 spectra × 2 *C*_a_ conditions); colours identify spectral recipes, and symbols identify *C*_a_. (**B**) Kinetic correlation loadings. (**C**) Response curve nonlinear iterative partial least squares (NIPALS) PCA scores from the 15 descriptors shown in Figure 3 and TPU_op_ across 32 plant × spectrum curve sets; large crosses indicate spectrum centroids. One boundary-constrained *g*_m_ estimate was unavailable and was not imputed. (**D**) Explained and cumulative variance for PC1–PC8. These components account for 98.50% of the kinetic variance and 98.37% of the response curve variance. The four response curve components meet the eigenvalue ≥ 1 criterion and together explain 90.08% of the variance. Variables were centred and scaled to unit variance; component signs are arbitrary. PCA summarises the covariance among the reported descriptors, including coefficients that are weakly constrained by individual curves; it does not identify independent biochemical mechanisms. The complete PC1–PC4 correlations are shown in Figure S1, and the sensitivity to alternative variable sets is reported in Supplementary Sections S10 and S11.

The kinetic principal component analysis (PCA) retained three components with eigenvalues of at least 1 (Figure 6A,B). PC1 explained 56.12% of the standardised variance and contrasted Φ_PSII_, *q*_P_, *q*_L_, *F*_v_′/*F*_m_′, and Φ_CO2_ with ETR, *q*_N_, *A*, and NPQ. This axis therefore represented the transition from efficient low-light photochemistry and open PSII reaction centres toward high-light electron transport, carbon gain, and regulated dissipation. PC2 explained 18.41% and contrasted *C*_i_, *E*, and *g*_sw_ with *i*WUE, resolving the stomatal and carbon–water dimensions of induction. Together, PC1 and PC2 accounted for 74.53% of the variance, and PC3 explained an additional 10.45%. The two *C*_a_ conditions separated primarily along the dimension associated with internal CO_2_, stomatal water flux, and *i*WUE, whereas spectral separation occurred mainly along the carbon‒photochemical axis.

For the response curve PCA (Figure 6C,D), PC1, PC2, PC3 and PC4 explained 46.41, 18.69, 15.85 and 9.14% of the variance, respectively (90.08% cumulative), and all four met the eigenvalue ≥ 1 criterion. PC1 represented the shared magnitude of light-response descriptors and stomatal conductance, with positive correlations for *A*_p,max_ (0.935), *R*_d,CO2_ (0.919), *A*_max_ (0.918), *g*_sw_ at PPFD 400 (0.889), *R*_d,light_ (0.777) and *α* (0.766). PC2 linked *g*_m_ (0.727), *A*_max,CO2_ (0.651), TPU_op_ (0.600) and *C*_c_ (0.507), in contrast to Γ_CO2_ (−0.649) and the light compensation point (−0.603). PC3 mainly described the variation in fitted *J*_max_ (0.840) and *V*_cmax_ (0.766), with negative contributions from *C*_c_ (−0.574) and Γ_CO2_ (−0.511). PC4 captured additional saturation and high-*C*_i_ variation, with correlations for the PPFD at 75% saturation (0.438), TPU_op_ (−0.414), *A*_max,CO2_ (−0.405) and *V*_cmax_ (0.395). The R90B10 centroid was positive on PC1, whereas R0B100 was negative on PC1 and PC2. The passive *A*-PPFD AUC_norm_ correlated with PC1 (0.914), whereas the *A*-*C*_i_ AUC_norm_ and CE correlated with PC1 (0.551 and 0.686) and PC2 (0.657 and 0.662), respectively. These axes integrate the reported curve descriptors under the stated fitting convention; their correlations do not by themselves establish independent physiological mechanisms.

The 30-curve and balanced 28-curve PCA sensitivities retained four components and explained 90.32% and 90.22% of the variance, respectively. The smallest canonical correlations between the four-dimensional score spaces on common observations were 0.9944 and 0.9939, respectively, whereas the largest angles between the corresponding loading subspaces were 18.37° and 18.32°. Thus, the overall score configuration remained close while the weights assigned to fitted coefficients changed materially. A separate PCA of nine light-response and empirical descriptors retained three components, which explained 93.96% of the variance. These checks support a descriptive integration of the measured response dimensions without interpreting the model-dependent axes as independently identified mechanisms (Supplementary Section S11, Table S14).

## 3. Discussion

### 3.1. Acute Spectral Regulation Links Capacity with Transition Physiology

The combined response curve and kinetic datasets show differences in response magnitude and temporal organisation among the acute spectral conditions. The separation of the *A*-PPFD AUC_norm_ was moderate, whereas the *A*-*C*_i_ profiles, induction AUC_bc_, PSII variables, quenching and *T*_leaf_ differed greatly. All the leaves developed under common white light, and the measurement order was randomised. These comparisons therefore concern acute chamber illumination rather than developmental spectral acclimation. The observed gas exchange and fluorescence responses support the consideration of the actinic recipe when interpreting leaf measurements, while the specific regulatory mechanisms require independent evidence [1,2,4,8,9,10,11,12,13,17,18,19,22,23].

R90B10 combined the highest *A*-PPFD AUC_norm_, the highest *α*, the largest induction amplitude, and the greatest assimilation AUC_bc_. Its larger absolute *C*_def_ does not indicate inferior induction. *C*_def_ integrates the absolute distance from a larger final *A*, whereas *t*_90_ expresses the time required to complete a fixed fraction of each treatment-specific amplitude. *A* treatment can therefore result in the acquisition of more carbon throughout induction while leading to the accumulation of a greater absolute deficit relative to an even higher final state. This distinction is central when comparing spectra or genotypes that differ in response amplitude: final capacity, fractional speed, and integrated carbon cost must be evaluated together [35,36].

The observed carbon response was nonmonotonic across the red:blue ratios. R90B10 had higher values for several carbon traits than the red-only and blue-rich conditions, whereas R0B100 had the lowest *A*-*C*_i_ AUC_norm_. This ranking concerns the measured leaf responses and does not establish an optimal cultivation spectrum. Studies in tomato have shown strong red:blue differences in steady-state photosynthesis with relatively small differences in induction, whereas work in wheat has shown faster induction with blue enrichment in several environments [17,37]. Species, photon absorption, growth history, irradiance and transition conditions may therefore influence how response magnitude and timing covary [16,17,18,19,20,21,22,23,37].

A possible explanation for the greater R90B10 response is that red-rich illumination supported carbon assimilation, whereas a small blue component contributed to guard-cell signalling or excitation regulation. The concurrent gas exchange and fluorescence profiles are consistent with this hypothesis but do not test these mechanisms directly. The *g*_sw_ AUC_bc_ contrast did not meet the family-adjusted criterion, and this nonsignificant result does not exclude a stomatal contribution. Separating diffusional, photochemical and biochemical contributions would require independent measurements [18,19,22,23].

The acute design defines the biological scope of the observations. Contrasting growth spectra can modify anatomy, pigments, stomatal density and source–sink relationships over days or weeks [16,17,18,19,20,21,24,25,28,29]. Here, leaves developed under common white light and experienced spectral changes only during measurement. These acute leaf responses provide physiological evidence for assessing how spectral composition and irradiance transitions jointly affect carbon gain and water loss, informing the evaluation of dynamic lighting strategies. The present rankings do not establish a cultivation spectrum or predict biomass or marketable yield.

### 3.2. Blue-Only Light Constrained the CO_2_-Response Capacity

R0B100 had the smallest AUC_norm_ across the commonly measured *C*_i_ domain and the lowest empirical *A*_max,CO2_ at a common incident PPFD. These are directly supported differences in the rapid operational *A*_‒_*C*_i_ profiles. Photon penetration, chloroplast positioning and excitation balance are plausible contributors because blue and red-rich light can affect these processes differently [18,19,22,23]. None were independently measured here, so the data do not reveal a unique mechanism.

R0B100 had a lower CE, empirical *A*_max,_ CO_2_ and operational TPU_op_, indicating a smaller carbon response across the measured *C*_i_ domain. This pattern can coexist with nonsignificant contrasts in fitted *g*_m_, *C*_c_, *V*_cmax_ and *J*_max_ because the empirical plateau and the mechanistic parameters contain different information. The plateau is strongly informed by the high-*C*_i_ observations, whereas joint FvCB parameter estimation depends on representation of the limiting regions and covariance among parameters [38,39,40,41,42,43,44,45,46,47]. Thirty of the 32 finite-*g*_m_ solutions contained no *A*_c_-limited observation points, so those curves provided little information about *V*_cmax_; the sensitivity of *J*_max_ and *g*_m_ also depended on the assumed light-response function and respiration offset. Accordingly, the reported FvCB estimates and their nominal comparisons are used only as secondary descriptions of the fitted solutions. The spectral conclusions rest on the complete observed response profiles, empirical descriptors and measured kinetic trajectories; neither an effect nor an absence of an effect on *V*_cmax_, *J*_max_ or *g*_m_ is inferred from these coefficient tests. Γ_app_ is an extrapolated empirical zero crossing, and Γ_CO2_ is a net compensation point derived from the FvCB model; neither is Γ*. TPU_op_ expresses the offset-corrected high-*C*_i_ assimilation flux on a three-carbon equivalent scale. Its lower value under R0B100 is consistent with lower high-*C*_i_ carbon throughput but does not independently indicate end-product utilisation or a unique TPU limitation [48,49]. Differences in excitation distribution, activation state, mesophyll transfer and downstream carbon metabolism remain plausible physiological explanations that require independent discrimination [18,19,43].

### 3.3. Responses to Elevated C_a_ Were Associated with Reorganised Carbon–Water Dynamics

At *C*_a_ = 2000 µmol mol^−1^, assimilation approached its destination state earlier, and the stomatal and transpiration amplitudes were lower than those at *C*_a_ = 400 µmol mol^−1^. The median *A t*_90_ occurred within the first recorded interval for the three spectra, whereas *g*_sw_ and *E* adjusted more slowly over smaller amplitudes. This pattern is consistent with published acute CO_2_ responses [30,31,32,33,34]. The *C*_a_ conditions were measured in separate sessions under their stated chamber-control settings; thus, the interpretation includes the effective microclimate and flow reported in Table 4. The absence of a *C*_a_ contrast in signed *A* AUC_bc_ does not contradict the shorter *t*_90_: AUC_bc_ includes amplitude and the full trajectory, whereas *t*_90_ measures fractional response timing.

These me_a_surements concern acute exposure of pre-existing leaves and do not measure long-term CO_2_ acclimation. Chronic enrichment can change Rubisco allocation, stomatal development and source–sink balance [30,31,32,33,34]. The spectral measurement order was randomised, and the flow targets were instrument-control settings rather than planned biological factors. Flow distributions and mean *T*_leaf_ and VPD_leaf_ differed between *C*_a_ conditions (Table 4); these effective conditions should accompany interpretation of the carbon–water responses. Plant pairing accommodates repe_a_ted observations, whereas standardised leaf rank maintains developmental comparability even when physical leaf identity changes. The between-session *C*_a_ comparisons therefore describe responses under the measured conditions; spectral randomisation and plant pairing do not establish th_a_t all session-associated influences were eliminated.

The *A*-*C*_i_ sequence began at ambient *C*_a_, decreased to 50 µmol mol^−1^ and then moved directly to 700 µmol mol^−1^ before it reached higher concentrations. This common path defines the rapid CO_2_-response profile compared across spectra. Measuring the low-*C*_a_ segment before the highest CO_2_ exposure standardised the sequence, while the fitted descriptors remained conditional on the acquisition path and duration [23,31,33,34]. Longer holds would quantify equilibr_a_tion, and return-to-ambient or reverse sequences would quantify history dependence under an additional protocol. The present comparison concerns the observed rapid profiles; the separate low–high–low irradiance series characterise the subsequent time course of carbon and water-flux adjustment.

Photon distribution within the lamina is one possible explanation for the differences between blue-only and red-rich profiles [19,50,51]. The concurrent fluorescence measurements also revealed differences in the PSII operating state and excitation-energy partitioning among the spectral conditions (Figure 5; Table 3). These observ_a_tions do not identify the underlying mechanism or demonstrate that CO_2_ caused the photochemical differences. NPQ and *q*_N_ describe nonphotochemical quenching; *q*_P_ and *q*_L_ estimate PSII reaction-centre openness under different connectivity assumptions and are not thermal dissipation measures [52,53,54,55,56,57,58,59,60]. Interpreting these variables jointly with *A* is useful for describing response coordination, provided that apparent ETR assumptions and rapid-acquisition assumptions are retained.

The downstep analysis revealed a pronounced carbon–water mismatch. *A* 90% transition toward the final low-light state occurred within the first recorded interval, but *g*_sw_ and *E* required approximately 12–16 min. Consequently, transpiration remained above its final low-light state after *A* had already completed most of its decline. *W*_exc_ was lower at *C*_a_ = 2000 µmol mol^−1^, even though the closing times were not faster, because the stomatal and transpiration amplitudes were smaller. Integrated water cost therefore depends on both the kinetic delay and the magnitude of the water-flux excursion. This direction-dependent lag is characteristic of stomatal hysteresis under fluctuating irradiance and has direct implications for carbon–water performance [61,62,63,64,65,66,67,68].

### 3.4. Photochemistry, Photoprotection, and Leaf Temperature Were Coordinated

R90B10 combined high assimilation with the greatest induction-phase NPQ response. This coexistence is physiologically coherent: NPQ is not evidence of poor performance by itself but a regulated route for dissipating excitation that exceeds instantaneous photochemical use. Under R90B10, ETR and *A* increased strongly, whereas Φ_PSII_, *q*_P_, and *q*_L_ decreased and NPQ increased. The concurrent decline in *F*_v_′/*F*_m_′ and Φ_PSII_ and increase in apparent ETR are not contradictory: PSII quantum efficiency decreased as the PPFD increased, while the greater incident photon flux still supported a greater electron-transport rate. The leaves therefore sustained greater electron transport and carbon assimilation while simultaneously increasing regulated thermal dissipation and reducing the proportion of open PSII centres. The opposing PC1 loadings captured this transition from low-light photochemical efficiency to high-light electron transport, carbon assimilation, and photoprotection [52,53,54,55,56,57,58,59,60,69].

The greatest increase in *T*_leaf_ and VPD_leaf_ occurred in R0B100. Leaf temperature reflects the balance among absorbed radiation, sensible heat exchange, boundary-layer conductance, stomatal water loss, and latent cooling; it cannot be assigned to wavelength alone [67,70]. In the present data, the thermal trajectory changed in parallel with spectral differences in *E*, *g*_sw_, and fluorescence, indicating that energy absorption and dissipation were reorganised together. *A* multispecies LI-6800 study likewise revealed that changes in Φ_PSII_ and *T*_leaf_ can track non-steady-state *A* and *g*_sw_, although the strength of those relationships varies among species [71]. *T*_leaf_ is therefore a complementary component of the measured physiology, not a substitute for gas exchange.

The differences in NPQ, *q*_N_, *q*_P_, *q*_L_, Φ_PSII_ and ETR remained after the irradiance returned to low light. The relaxation phase therefore contains information about the state carried from the preceding high-light phase. Persistent quenching can influence photon use during subsequent transitions, as shown in phase-specific studies [66,67]. Our single low–high–low event describes this carry-over but cannot establish repeated-cycle behaviour or daily productivity.

### 3.5. Multivariate Integration, Experimental Scope, and Implications

The two PCAs integrate complementary dimensions of the response curves and kinetics. The kinetic ordination links carbon and photochemical adjustment with the stomatal-water response. In the response curve PCA, PC1 combines the light-response magnitude, stomatal conductance and respiration terms; PC2 links mesophyll and chloroplastic CO_2_ descriptors with empirical high-*C*_i_ assimilation and TPU_op_; PC3 reflects the variation in the fitted carboxylation and electron-transport parameters; and PC4 describes the additional saturation structure. Together, the four components explain 90.08% of the variance. These are descriptive covariance dimensions: several variables are derived from the same curves, *A*_p,max_ = *A*_max_ + *R*_d,light_ is an exact relationship, and uncertainty in coupled FvCB parameters can influence their loadings. The original multivariate framework is therefore retained alongside a targeted sensitivity analysis using the 15 Figure 3 descriptors. This analysis retains four components and 89.78% of the variance, with canonical correlations of 1.00000, 0.99999, 0.99975 and 0.98995 relative to the original four-component score subspace. This comparison concerns variable selection and does not establish the stability of individual FvCB coefficients. The complementary boundary-influence analysis is reported in Section S11. The integrated descriptor framework is interpreted jointly with curve-level comparisons [72].

Common growth conditions, standardised leaf rank, randomised measurement order and repeated plant measurements support the assessment of acute spectral responses in developed leaves. The order R100B0, R90B10, R50B50 and R0B100 is used for presentation and does not describe treatment allocation. Initial and final low-light phases provide direct observations of starting states and relaxation, with relatively stable initial assimilation tails and different relaxation patterns for stomatal conductance and transpiration (Supplementary Section S6). The experiment characterises one cultivar and a single low–high–low event at approximately 1 min resolution. It does not directly measure repeated-cycle memory, canopy integration, biochemical enzyme capacity or long-term cultivation performance.

The response profiles also define distinct questions for subsequent experiments. Future comparisons of acquisition durations could test the relationships between rapid and fully equilibrated curves, whereas repeated irradiance cycles, additional genotypes and cultivation experiments could test transfer to daily carbon gain and growth. These extensions address protocol dependence and biological generality beyond the present acute leaf comparison. Higher-frequency acquisition during the first 2 min would resolve the fastest assimilation responses more precisely [35,71].

Standardising the fourth or fifth fully expanded leaf was particularly important because developmental age can alter dynamic behaviour without producing proportional changes in steady-state capacity. Older cucumber leaves, for example, show slower photosynthetic induction and stomatal opening and closing despite retaining similar steady-state traits [68]. Restricting leaf rank therefore reduced developmental variation and strengthened the physiological interpretation of the spectral comparison. The concurrent *T*_leaf_ trajectory adds a further integrative dimension because rapid thermal and VPD_leaf_ changes can modify *A* and *g*_sw_ during irradiance transitions [70]. Treating *T*_leaf_ and VPD_leaf_ as part of the measured response, rather than as passive consequences of illumination, consequently, strengthens the interpretation of spectral effects on carbon‒water coordination and supports the consideration of the leaf energy balance as an active component of dynamic photosynthesis.

The first hypothesis was supported by the greater integrated rapid response to red-rich recipes than blue-only light. R90B10 produced the highest *A*-PPFD AUC_norm_ and apparent quantum yield, and R100B0 and R90B10 formed the highest empirical *A*-*C*_i_ respo_n_se group. The second hypothesis was also supported: response magnitude and fractional speed were not interchangeable. R90B10 achieved the greatest induction amplitude and integrated carbon gain without being uniformly the fastest recipe; its larger absolute *C*_def_ reflected the distance to a higher final assimilation state. The responses at *C*_a_ = 2000 µmol mol^−1^ were consistent with the third hypothesis, combining earlier assimilation induction with smaller stomatal and transpiration amplitudes under the reported chamber conditions. After the light decreased, *A* approached its low-light state within the first observation interval, while *g*_sw_ and *E* required approximately 12–16 min, sustaining postillumination water loss. The linked curves, fluorescence measurements and tr_a_nsition kinetics therefore reveal complementary aspects of acute spectral regulation and carbon–water coordination in chicory leaves.

## 4. Materials and Methods

### 4.1. Plant Material and Growth Conditions

Seeds of chicory (*Cichorium intybus* L. cv. Pão-de-Açúcar) were germinated in 96-cell trays containing a pine-bark-based commercial horticultural substrate (MecPlant, Telêmaco Borba, Paraná, Brazil). After the emergence of two true leaves, the seedlings were transplanted into individual 750 mL pots containing the same substrate. Physiological measurements were conducted during the linear vegetative phase of rosette growth on an intact, fully expanded leaf in the fourth or fifth position counted from the apex.

The plants received full-strength (1× full-power; 50 mL per application) complete Hoagland nutrient solution every 2 d, adjusted to a pH of 5.3. Water was supplied daily at approximately 08:00 and 18:00 to maintain a uniformly moist substrate without prolonged drainage. This irrigation regime was used to maintain active photosynthesis and prevent water deficit from becoming a co-limiting factor during gas exchange measurements because of the long photoperiod and the time required to complete all the photosynthetic curves during the plants’ linear growth phase.

The plants were grown under commercially available neutral-white light-emitting diode (LED) luminaires (Philips, Signify Netherlands B.V., Eindhoven, The Netherlands) at a photosynthetic photon flux density (PPFD) of 500 µmol photons m^−2^ s^−1^, with a 16 h light/8 h dark photoperiod and an air temperature of 22–26 °C. The nominal daily light integral was 28.8 mol photons m^−2^ d^−1^. All the plants developed in this common white-light environment; red:blue treatments were imposed only within the LI-6800 measurement chamber. Twenty uniform, healthy plants without visible mechanical injury, nutrient deficiency symptoms, or pests were maintained, from which eight were selected at random for the complete physiological experiment.

Measurements were conducted over 10 d, between 08:00 and 18:00, in the laboratory under controlled environmental conditions and the common 16 h light/8 h dark regime. At each measurement session, an intact, fully expanded leaf occupying the fourth or fifth position from the apex was selected. Because new leaves emerged during this measurement period, the physical leaves used could differ among the sessions such that the leaf rank and developmental status, rather than the leaf identity, remained standardised. Within each complete *A*-PPFD curve, *A*-*C*_i_ curve, or kinetic series, all sequential observations were obtained from the same enclosed leaf area.

Spectral measurement order was randomised within the repeated plant-level comparisons. All *A*-PPFD and *A*-*C*_i_ response curves were obtained before the kinetic measurements. Randomisation refers to the spectral comparisons, rather than the prescribed PPFD and *C*_a_ steps within each complete curve or trajectory. The spectra are presented in the common order R100B0 (100% red:0% blue), R90B10 (90% red:10% blue), R50B50 (50% red:50% blue) and R0B100 (0% red:100% blue); this is the reporting order. Each kinetic trajectory followed the low–high–low irradiance programme at a reference *C*_a_ of either 400 or 2000 µmol mol^−1^, with the two *C*_a_ conditions measured in separate sessions. Between complete protocols, the plants were returned to the common growth environment before re-enclosure, analyser matching and chamber equilibration.

Biological independence was defined at the plant level. Each of the eight plants contributed one complete response curve or kinetic trajectory under each applicable treatment condition. Sequential values recorded within a curve or trajectory were repeated physiological observations from the same enclosed leaf area and were not treated as additional biological replicates. Repeated treatment conditions within plants were accommodated by paired or repeated-measures analyses.

### 4.2. Gas-Exchange System, Chamber Conditions, and Actinic Spectra

Gas exchange and modulated chlorophyll *a* fluorescence were measured simultaneously from the same 6 cm^2^ leaf area using an LI-6800 portable photosynthesis system fitted with a 6800–01A fluorometer chamber (LI-COR Biosciences, Lincoln, NE, USA). The system was warmed, leak-checked, matched, and examined for stable analyser operation before measurements. The fan speed was maintained near 10,000 rpm, the differential pressure (Δ*P*) was maintained at 0.2 kPa, the reference O_2_ concentration was 21%, the chamber block temperature was maintained near 25 °C, and the relative humidity (RH) was maintained near 65%. Open-system CO_2_ and H_2_O mass balances provided net CO_2_ assimilation (*A*) and transpiration (*E*); stomatal conductance to water vapour (*g*_sw_) and intercellular CO_2_ mole fraction (*C*_i_) were calculated with the instrument conductance equations, including boundary-layer terms. The reference CO_2_ mole fraction (*C*_a_), total flow, leaf temperature (*T*_leaf_), RH, and leaf-to-air vapour-pressure deficit (VPD_leaf_) were retained for every observation (Table 4).

Four acute actinic recipes were generated with the 6800–01A red channel (nominal peak 625 nm) and blue channel (nominal peak 475 nm): R100B0 (100% red:0% blue), R90B10 (90% red:10% blue), R50B50 (50% red:50% blue), and R0B100 (0% red:100% blue) (LI-COR Biosciences, Lincoln, NE, USA). The labels refer to programmed channel contributions to the total chamber PPFD. Instrument-reported photon fractions were calculated as *f*_R_ = *Q*_R_/(*Q*_R_ + *Q*_B_) and *f*_B_ = *Q*_B_/(*Q*_R_ + *Q*_B_); the far-red output (nominal peak at 735 nm) was set to zero so that the experiment isolated red and blue actinic effects (LI-COR Biosciences, Lincoln, NE, USA). After leaf enclosure, the target conditions were approached for 5 min. The sample and reference analysers (ΔCO_2_ and ΔH_2_O in both the reference and sample chambers) were then matched to correct analyser offsets, and the signals were allowed to stabilise for an additional 3 min before acquisition. Matching was repeated after large changes in *C*_a_ or in the reference and sample IRGA signals.

The total chamber flow was selected for each protocol to maintain stable reference and sample differentials, *C*_a_, RH, and chamber pressure: 1200 µmol s^−1^ for *A*-PPFD curves, predominantly 800 µmol s^−1^ for *A*-*C*_i_ curves, 800 or 1200 µmol s^−1^ during kinetics at *C*_a_ = 400 µmol mol^−1^, and predominantly 750 µmol s^−1^ during kinetics at *C*_a_ = 2000 µmol mol^−1^. Within each protocol setting, the LI-6800 automatic mass-flow control maintained the programmed condition. Flow was not a spectral treatment, and the four spectra were measured under the same chamber-control configuration within each plant-level protocol block. This ensured stable analyser operation, maintained reference and sample IRGA control, and allowed the data to be compared under the programmed LI-6800 settings without operator adjustment. The effective conditions are summarised in Table 4.

The recorded variables were *A*, *E*, *g*_sw_, *C*_i_, *T*_leaf_, RH, and VPD_leaf_. Intrinsic water use efficiency was calculated as *i*WUE = *A*/*g*_sw_, stomatal conductance to CO_2_ as *g*_sc_ = *g*_sw_/1.6, and instantaneous water use efficiency as WUE = *A*/*E* [38,39].

$$\begin{array}{c} iWUE=\frac{A}{g_{\mathrm{sw}}}\\ g_{\mathrm{sc}}=\frac{g_{\mathrm{sw}}}{1.6}\\ WUE=\frac{A}{E} \end{array}$$

### 4.3. Stepwise A-PPFD and A-C_i_ Response Curves

Rapid stepwise *A*-PPFD curves were obtained under each spectral recipe using ten decreasing irradiance levels: 1000, 800, 600, 400, 250, 150, 100, 60, 30 and 0.05 µmol photons m^−2^ s^−1^. The reference *C*_a_ concentration was 400 µmol mol^−1^. After initial chamber preparation, the program allowed recording after a minimum of 50 s when its differential-signal criteria were met or at a maximum wait of 60 s. The criteria for differential CO_2_ were slope and standard-deviation limits of 0.1 and 0.1, respectively; the corresponding H_2_O limits were 0.5 and 0.1. All observations and their recorded stability classifications were retained. Eight complete plant-level curves were measured per spectrum. The common descending sequence standardised the irradiance protocol for comparison of apparent quantum yield, saturation, the light-curve respiration term and stomatal response. These are rapid operational curves: analyser stability is distinct from full physiological equilibration, and *R*_d,light_ is an apparent fitted offset. Retained stability classifications are reported in Supplementary Section S6. The comparison concerns complete rapid response profiles acquired with a common program; prolonged equilibration would define a separate acquisition protocol.

Rapid _A‒Ci_ curves were measured at a PPFD of approximately 1000 µmol photons m^−2^ s^−1^. The reference *C*_a_ concentrations were 400, 300, 200, 100, 50, 700, 1000, 1500, and 2000 µmol mol^−1^, with approximately 60.5 s between the retained records. The sequence proceeded directly from 50 to 700 µmol mol^−1^, without an intervening ambient-CO_2_ step. The measured *C*_i_, rather than the programmed *C*_a_, was used as the response-axis value. Eight complete plant-level curves were obtained per spectrum. Each plant constituted an independent biological replicate and contributed one complete response curve under each spectral condition. Consequently, at each *C*_a_ step, the eight replicate observations originated from eight different plants. The sequential observations within each plant-level curve were retained as ordered response measurements for empirical and Farquhar–von Caemmerer–Berry (FvCB) model fitting and were not counted as additional biological replicates. These discrete stepwise curves describe the specified rapid response protocol. Curve-level summaries and fitted parameters were subsequently obtained once per plant and spectrum [73].

### 4.4. Chlorophyll Fluorescence and Derived Variables

In a separate measurement, leaves were dark-adapted for 12 h before minimum fluorescence (*F*_o_) and maximum fluorescence (*F*_m_) were obtained with the rectangular saturation routine set to 10,000 µmol photons m^−2^ s^−1^. For each measured leaf, *F*_o_ and *F*_m_ were determined after 12 h of dark adaptation before the corresponding light-adapted fluorescence protocol. During light-adapted gas-exchange measurements, steady fluorescence (*F*_s_) was recorded, and maximum light-adapted fluorescence (*F*_m_′) was determined with the built-in multiphase-flash routine at 10,000 µmol photons m^−2^ s^−1^. The dark modulation rate was set to 500 Hz, and the light-adapted fluorescence acquisition settings were a light modulation rate of 5 kHz and a flash modulation rate of 250 kHz. All the spectra used identical fluorescence settings. The variable fluorescence was *F*_v_ = *F*_m_ − *F*_o_, the maximum PSII quantum efficiency was *F*_v_/*F*_m_, and the light-adapted variables followed standard pulse-amplitude-modulated definitions [52,53,54,55,56,57,58,59,69].

$$\begin{array}{c} F_{v}=F_{m}-F_{o}\\ \frac{F_{v}}{F_{m}}=\frac{F_{m}-F_{o}}{F_{m}}\\ \frac{{F_{v}}^{\prime }}{{F_{m}}^{\prime }}=\frac{{F_{m}}^{\prime }-{F_{o}}^{\prime }}{{F_{m}}^{\prime }}\\ \Phi_{\mathrm{PSII}}=\frac{{F_{m}}^{\prime }-F_{s}}{{F_{m}}^{\prime }} \end{array}$$

The operating quantum yield of PSII (Φ_PSII_), light-adapted potential PSII efficiency (*F*_v_′/*F*_m_′), apparent electron transport rate (ETR), apparent quantum yield of CO_2_ assimilation (Φ_CO2_), nonphotochemical quenching (NPQ), photochemical quenching coefficient (*q*_P_), nonphotochemical quenching coefficient (*q*_N_), and lake-model coefficient (*q*_L_) were calculated from the displayed equations. The ETR calculation assumed a leaf absorptance of *α*_leaf_ = 0.85 and equal partitioning of absorbed photons to PSII (0.50) and therefore represents apparent PSII electron transport. *q*_L_ estimates the fraction of open PSII reaction centres under the lake model [60].

$$\begin{array}{c} \mathrm{ETR}=\Phi_{\mathrm{PSII}}\times \mathrm{PPFD}\times \alpha_{\mathrm{leaf}}\times 0.50\\ \Phi_{CO2}=\frac{A}{\mathrm{PPFD}}\\ \mathrm{NPQ}=\frac{F_{m}-{F_{m}}^{\prime }}{{F_{m}}^{\prime }}\\ q_{P}=\frac{{F_{m}}^{\prime }-F_{s}}{{F_{m}}^{\prime }-{F_{o}}^{\prime }} \end{array}$$

$$\begin{array}{c} q_{N}=1-\frac{{F_{m}}^{\prime }-{F_{o}}^{\prime }}{F_{m}-F_{o}}\\ q_{L}=q_{P}\frac{{F_{o}}^{\prime }}{F_{s}} \end{array}$$

$${F_{o}}^{\prime }=\frac{F_{o}}{\frac{F_{v}}{F_{m}}+\frac{F_{o}}{{F_{m}}^{\prime }}}$$

When *F*_o_′ was not measured directly, it was estimated from *F*_o_, *F*_v_/*F*_m_, and *F*_m_′ using the displayed Oxborough–Baker relation [74,75]. *F*_o_, *F*_m_, *F*_v_, and *F*_v_/*F*_m_ were retained as dark-adapted baseline and computational quantities and were not treated as acute spectral-response endpoints because they were obtained before application of the corresponding actinic recipe. NPQ and *q*_N_ were treated as related nonphotochemical quenching descriptors, whereas *q*_P_ and *q*_L_ estimate PSII reaction-centre openness under the puddle and lake connectivity models, respectively.

### 4.5. Curve Models and Physiological Parameters

The *A*-PPFD curves were fitted with the nonrectangular hyperbola displayed below, where *Q* is the PPFD, *α* is the initial apparent quantum yield, *P*_max_ is the asymptotic gross assimilation rate, *θ* is the curvature, and *R*_d,light_ is the apparent light-curve respiration term [76]. Bounded robust nonlinear least squares used multiple starting values with 0.00001 ≤ *α* ≤ 0.5, 0.05 ≤ *P*_max_ ≤ 300, 0.01 ≤ *θ* ≤ 0.999, and 0 ≤ *R*_d,light_ ≤ 30.

$$A(Q)=\frac{\alpha Q+P_{\max}-\sqrt{{\left(\alpha Q+P_{\max}\right)}^{2}-4\theta \alpha QP_{\max}}}{2\theta }-R_{d,\mathrm{light}}$$

The derived light-response quantities were apparent maximum gross assimilation (*A*_p,max_ = *P*_max_), apparent maximum net assimilation (*A*_max_ = *P*_max_ − *R*_d,light_), *α*, *R*_d,light_, the light-compensation point (LCP) defined by *A*(*Q*) = 0, and the first modelled *Q* reaching 75% of the response amplitude above *A*(*Q* = 0), which was used as the operational light saturation point (LSP). *g*_sw_ at a PPFD of 400 µmol photons m^−2^ s^−1^ was obtained by linear interpolation. The empirical *A*-*C*_i_ plateau was fitted with the displayed saturating exponential, where the carboxylation efficiency (CE) is the slope at *C*_i_ = Γ_app_ and Γ_app_ is the apparent *C*_i_ compensation point. *A*_max,CO2_, CE, and Γ_app_ were constrained to 0.05–500 µmol CO_2_ m^−2^ s^−1^, 0.00001 ≤ CE ≤ 5 mol m^−2^ s^−1^, and −500 to 1000 µmol mol^−1^, respectively.

$$A=A_{\max,CO2}\left[1-\exp\left\{-\frac{\mathrm{CE}(C_{i}-\Gamma_{\mathrm{app}})}{A_{\max,CO2}}\right\}\right]$$

For the empirical exponential, d*A*/d*C*_i_ = CE exp[−CE(*C*_i_ − Γ_app_)/*A*_max,CO2_], so CE is the tangent at *C*_i_ = Γ_app_. Its units are (µmol CO_2_ m^−2^ s^−1^)/(µmol CO_2_ mol^−1^), which is equivalent to mol m^−2^ s^−1^. Γ_app_ is an extrapolated zero crossing below the measured *C*_i_ range and is distinct from the specified photorespiratory Γ*.

The FvCB framework describes C_3_ assimilation as the minimum of Rubisco-carboxylation-limited (*A*_c_), electron-transport-limited (*A*_j_), and triose-phosphate-utilisation-limited (*A*_p_) rates after accounting for the respiration term [40]. The model was evaluated across the measured *C*_i_ sequence so that each retained point contributed to the fitted limitation region associated with the lowest predicted rate.

$$\begin{array}{c} A=\min(A_{c},A_{j},A_{p})-R_{d}\\ A_{c}=\frac{V_{c\max}(C_{c}-\Gamma^{*})}{C_{c}+K_{c}(1+\frac{O}{K_{o}})}\\ A_{j}=\frac{J(C_{c}-\Gamma^{*})}{4C_{c}+8\Gamma^{*}}\\ A_{p}=3\mathrm{TPU} \end{array}$$

In these expressions, Γ* is the CO_2_ compensation point in the absence of day respiration, *J* is the electron-transport rate, *K*_c_ and *K*_o_ are the Michaelis–Menten constants of Rubisco for CO_2_ and O_2_, respectively, and *O* is the chloroplastic O_2_ concentration.

The FvCB fits accounted for finite mesophyll conductance [41,42,43,44,45,46,47]. The nonrectangular light hyperbola supplied *A*_p,max_, *A*_max_, *α*, *R*_d,light_, the light compensation point and the PPFD at 75% modelled saturation; *g*_sw_ at PPFD 400 was interpolated. The empirical exponential supplied *A*_max,CO2_, CE and Γ_app_. The ambient summary of the finite-*g*_m_ solution supplied *g*_m_ and *C*_c_, whereas its full-curve fit supplied *V*_cmax_, *J*_max_ and Γ_CO2_. The separate apparent-*C*_i_ FvCB solution supplied *C*_i,90_, defined as the first *C*_i_ reaching 90% of its modelled response amplitude. In the finite-*g*_m_ fit, *R*_d,CO2_ was fixed to the paired Yin fluorescence-based light-response estimate; it was not freely estimated from *A*-*C*_i_ observations. *C*_c_ was calculated as *C*_i_ − *A*_model_/*g*_m_, using fitted net assimilation and temperature-adjusted conductance. *J*(*Q*) was linked to *J*_max_ through the displayed nonrectangular hyperbola with *α*_J_ = 0.30 and *θ*_J_ = 0.70; these coefficients were fixed across the spectra. *J*_max_ is thus the fitted asymptote under that relationship, which is distinct from *J* at the measurement irradiance and apparent fluorescence ETR. The full equations, bounds, kinetic constants, pressure and temperature corrections, optimisation settings, individual fits and uncertainties are provided in Supplementary Sections S5, S8 and S9 [77,78,79]. The R100B0 *g*_m_ summary uses the seven non-boundary estimates; plant 3’s upper-bound estimate is reported individually in Table S10. Operational TPU_op_ was calculated for each plant × spectrum curve as the median[(*A* + *R*_d,CO2_)/3] over the retained measured *C*_i_ ≥ 800 µmol mol^−1^. Its values are apparent respiration-corrected high-*C*_i_ assimilation expressed in triose-phosphate equivalents. This proxy describes carbon throughput under the specified protocol and is not an independently identified biochemical TPU capacity.

$$J(Q)=\frac{\alpha_{J}Q+J_{\max}-\sqrt{{\left(\alpha_{J}Q+J_{\max}\right)}^{2}-4\theta_{J}\alpha_{J}QJ_{\max}}}{2\theta_{J}}$$

$${\mathrm{TPU}}_{\mathrm{op}}=\underset{C_{i}\geq 800}{\mathrm{median}}\left[\frac{A+R_{d,CO2}}{3}\right]$$

### 4.6. Low–High–Low Light Kinetics

Each kinetic series lasted 15 min at a PPFD of 50 µmol photons m^−2^ s^−1^, 40 min at a PPFD of 1000 µmol photons m^−2^ s^−1^, and 20 min after the return to a PPFD of 50 µmol photons m^−2^ s^−1^. The median acquisition interval was 60 s. Reference *C*_a_ was maintained at either 400 or 2000 µmol mol^−1^ throughout each series; the low–high–low transition therefore concerned irradiance. The *C*_a_ = 400 and 2000 µmol mol^−1^ series were conducted in separate measurement sessions. Eight plants were measured under each of the four spectra at both carbon levels, yielding 64 complete series. The plant was the biological unit, and the observations within a series constituted one physiological trajectory. The initial low-light assimilation tails were relatively stable: the median tail ranges were 0.40% and 1.23% of the subsequent induction amplitude in the *C*_a_ = 400 and 2000 sessions, respectively. Water-flux tails and final-low versus initial-low states showed greater variation (Supplementary Section S6). The initial and final low-light observations characterise the measured starting states and variable-specific relaxation under the common kinetic programme.

For each transition and variable, *X*_0_ was the median of the final 20% of observations in the origin phase, and *X*_f_ was the corresponding median in the destination phase, with at least three observations per tail. The response amplitude was Δ*X* = *X*_f_ − *X*_0_. The signed baseline-corrected area (AUC_bc_) was calculated once per series by trapezoidal integration of *X*(*t*) − *X*_0_, expressed in the physical response unit multiplied by minutes. No smoothing was applied to AUC_bc_.

$${\mathrm{AUC}}_{\mathrm{bc}}=\sum_{j=1}^{k-1}\frac{\left(X_{j}-X_{\mathrm{base}})+(X_{j+1}-X_{\mathrm{base}}\right)}{2}(t_{j+1}-t_{j})$$

Directional first-passage times *t*_10_, *t*_50_, *t*_63.2_, and *t*_90_ were defined as the first crossings of 10, 50, 63.2, and 90% of Δ*X*, respectively. Crossing times were obtained by linear interpolation between adjacent observations without extrapolation. *A* centred three-observation median was used only to identify crossings. For *A*, *g*_sw_, and *E*, a first-order response with lag *L* and time constant *τ* was fitted as a secondary descriptive model. *A* fit was retained for reporting when at least four observations lay between 4 and 96% of the normalised transition progress, the log-linear slope was negative, *L* ≥ 0, *τ* > 0, and *R*^2^ ≥ 0.80. Threshold times and integrated costs remained the primary comparisons because they quantify observed transition progress without requiring a single exponential shape.

$$X(t)=X_{f}+(X_{0}-X_{f})\exp\left[-\frac{t-L}{\tau }\right],t\geq L$$

Integrals at 300 and 700 s were restricted to the observed time domain. The first observation in the destination phase occurred 60–119.9 s after the final observation of the preceding phase. *A* threshold crossed before that first record is therefore reported as “by first measurement”, which identifies the observed interval containing the response rather than assigning an unmeasured instantaneous time.

### 4.7. Kinetic Response Times, Stabilisation, and Transition Costs

The timing summaries for *A*, *g*_sw_, and *E* included trajectories whose Δ*X* followed the expected direction, increasing after the 50–1000 µmol photons m^−2^ s^−1^ step and decreasing after the 1000–50 µmol photons m^−2^ s^−1^ step, and whose robust amplitude signal-to-noise ratio was at least 3. MAD_0_ and MAD_f_ are the median absolute deviations of the origin and destination tails, respectively. Response-time, stomatal opening, stomatal closing, and integrated cost calculations followed current guidance for dynamic photosynthesis [35,71,80].

$$\begin{array}{c} {\mathrm{SNR}}_{\mathrm{amp}}=\frac{\left|X_{f}-X_{0}\right|}{1.4826\times \max({\mathrm{MAD}}_{0},{\mathrm{MAD}}_{f})}\\ P_{X}(t)=100\frac{X(t)-X_{0}}{X_{f}-X_{0}}\\ {\mathrm{RT}}_{\mathrm{stab}}=100\frac{t_{\mathrm{stab}}}{T_{\mathrm{observed}}} \end{array}$$

Sustained stabilisation was the first time a complete 180 s window was initiated within *X*_f_ ± max(0.10|Δ*X*|, 2.5 MAD_f_), provided that at least 80% of all later observations remained within that band and that no three consecutive observations occurred outside it. RT_stab_ expresses *t*_stab_ as a percentage of the observed duration of the destination phase, typically 39 min for induction and 19 min for relaxation.

$$\begin{array}{c} d=\mathrm{sign}(X_{f}-X_{0})\\ D_{X}=\int_{0}^{T}\max\{0,d[X_{f}-X(t)]\}dt\\ C_{\mathrm{def}}=\frac{60}{1000}\int_{0}^{T}\max[0,A_{f}-A(t)]dt\\ W_{\mathrm{exc}}=60\int_{0}^{T}\max[0,E(t)-E_{f}]dt \end{array}$$

The direction factor *d* standardised an increase or decrease to positive progress, and *D*_X_ is the direction-standardised distance-to-final integral for a generic response *X*. For the induction of *A*, *C*_def_ integrated the assimilation remaining below *A*_f_; for the relaxation of *E*, *W*_exc_ integrated transpiration above *E*_f_. With time expressed in minutes, the displayed factors convert *C*_def_ to mmol CO_2_ m^−2^ and *W*_exc_ to mmol H_2_O m^−2^. These quantities combine the transition amplitude and duration and are interpreted together with *t*_90_. Table 2 reports |Δ*A*| so that induction and relaxation can be compared as transition magnitudes. Before every series, leaves underwent the same enclosure, analyser matching, equilibration, and stability checks described for the *A*-PPFD and *A*-*C*_i_ response curves.

### 4.8. Integrated Response Curve Analysis, Blocked Inference, and Principal Component Analysis

For AUC_norm_, observations were ordered by measured PPFD or *C*_i_ before integration. Values at common boundaries were obtained by linear interpolation between adjacent points, followed by trapezoidal integration and division by domain width. The shared domains were 0.05–1000 µmol photons m^−2^ s^−1^ for *A*-PPFD and 58–1380 µmol mol^−1^ for *A*-*C*_i_.

$${\mathrm{AUC}}_{\mathrm{norm}}=\frac{1}{x_{\max}-x_{\min}}\sum_{j=1}^{k-1}\frac{y_{j}+y_{j+1}}{2}(x_{j+1}-x_{j})$$

For complete curve-level AUC_norm_, the spectrum was tested as a within-plant factor by repeated-measures analysis of variance (ANOVA). The Greenhouse–Geisser correction adjusted the degrees of freedom when within-subject sphericity was not met. Holm-adjusted paired comparisons controlled the familywise error rate across spectral contrasts, and Friedman tests provided a distribution-free assessment of the same within-plant ranking. Every fitted endpoint reported in Figure 3 or Table 1 was analysed from one curve-level estimate per plant and spectrum. The linear model included spectrum and plant as fixed terms, used HC3 heteroscedasticity-consistent covariance to reduce small-sample sensitivity to unequal residual variance, and tested the three spectrum coefficients jointly. Holm-adjusted paired comparisons were interpreted after a significant omnibus spectrum test. Available paired plants were used for endpoints with a missing estimate. The partial η_p_^2^ was calculated as the spectrum sum of squares divided by the sum of the spectrum and residual sums of squares [81]. For *g*_m_, *C*_c_, *V*_cmax_, *J*_max_, Γ_CO2_ and *C*_i,90_, the comparisons were treated as secondary descriptive summaries of the fitted solutions because parameter identification depends on branch coverage and fitting assumptions. They were not used to establish biochemical treatment effects or physiological equivalence. Parameter-bound and stability-classification sensitivities were evaluated separately without replacing the primary observations or fits (Supplementary Sections S11 and S12).

Signed kinetic AUC_bc_ was analysed separately for each transition and response in a 4 × 2 repeated-measures design, with spectrum and *C*_a_ as within-plant factors and plant as the subject. Greenhouse–Geisser correction was applied to the spectrum and spectrum × *C*_a_; *C*_a_ had two levels and required no correction. The Benjamini‒Hochberg procedure controlled the false discovery rate within each transition, model effect, and physiological family: gas exchange (*A*, *C*_i_, *g*_sw_, *E*, *i*WUE), chamber microclimate (VPD_leaf_, *T*_leaf_), photochemical efficiency (*F*_v_′/*F*_m_′, Φ_PSII_, ETR, Φ_CO2_), and quenching/partitioning (NPQ, *q*_N_, *q*_P_, *q*_L_). Table 3 reports the nominal *p* and family-adjusted *q*, with *α* = 0.05. *C*_def_ and *W*_exc_ were analysed with the same 4 × 2 structure as a separate two-endpoint multiplicity family. Timing descriptors are reported as medians and interquartile ranges.

Principal component analysis was performed after centring and scaling each variable to unit variance. The kinetic PCA integrated 15 signed-induction AUC_bc_ descriptors across the 64 plant × spectrum × *C*_a_ series. The response curve PCA integrated the 15 Figure 3 descriptors—*A*_p,max_, *A*_max_, *α*, *R*_d,light_, light-compensation point, and PPFD at 75% modelled saturation; *g*_sw_ at PPFD 400, *g*_m_, *C*_c_, *A*_max,CO2_, *R*_d,CO2_, *V*_cmax_, *J*_max_, Γ_CO2_ and *C*_i,90_; and operational TPU_op_ across 32 plant × spectrum curve sets. The *g*_m_ cell for R100B0 plant 3 was left missing because its estimate reached the upper bound; no value was imputed. NIPALS uses least-squares score/loading updates and sequential deflation over observed cells, with a relative convergence tolerance of 10^−12^. *A*-PPFD AUC_norm_, *A*-*C*_i_ AUC_norm_ and CE were projected passively as correlations with the scores. Equivalent eigenvalues were variance proportions multiplied by 16; components with eigenvalues ≥ 1 were retained, and signs were treated as arbitrary [72]. A targeted sensitivity used the 15 Figure 3 descriptors to assess the influence of TPU_op_. Additional analyses compared the primary 32-curve matrix with 30- and balanced 28-curve matrices, each re-centred and re-scaled. Section S11 specifies their plant and spectrum composition. These analyses assess the dependence of the descriptive ordination on particular coefficients; they do not replace the complete dataset. *A* further sensitivity used all 32 curve sets and the nine light-response and empirical descriptors specified in Section S11. Score-space comparisons used common curve rows and accounted for the different number of retained components.

Statistical analyses and graph preparation were performed using RStudio version 2026.06 (Posit Software, PBC, Boston, MA, USA), SigmaPlot version 16 (Systat Software, Inc., San Jose, CA, USA), Statistica version 12 (StatSoft, Inc., Tulsa, OK, USA), Python version 3.13 (Python Software Foundation, Beaverton, OR, USA), Unscrambler X version 10.4 (CAMO Software AS, Oslo, Norway), Microsoft 365 (Microsoft Corporation, Redmond, WA, USA), and CorelDRAW 2025 (Alludo, Ottawa, ON, Canada). Additional calculations used Python 3.12 with NumPy, SciPy and pandas.

## 5. Conclusions

The acute red:blue composition affected the recorded response curve profile and transition physiology of white-light-grown leaf chicory. R90B10 provided the strongest overall carbon response, combining the highest normalised *A*-PPFD area, apparent quantum yield, induction amplitude and integrated carbon gain. R100B0 and R90B10 had the greatest empirical *A*-*C*_i_ responses, whereas R0B100 had the smallest response and operational high-*C*_i_ TPU proxy. These findings show that response capacity, amplitude and fractional timing are complementary traits. At *C*_a_ = 2000 µmol mol^−1^, assimilation approached its final state earlier, and the stomatal and transpiration amplitudes were lower under the reported chamber conditions. After the irradiance decreased, assimilation completed most of its response within the first recorded interval, while stomatal conductance and transpiration required approximately 12–16 min, sustaining postillumination water loss. Carbon gain and water use were therefore imperfectly coordinated, and integrated water cost depended on both response delay and flux amplitude. The actinic spectrum should be reported as part of gas-exchange and fluorescence measurement protocols. Further work can test the transfer of these acute leaf responses to repeated irradiance cycles, genotypes, canopy assimilation and cultivation performance.

## Figures and Tables

**Table 1:** The complete curve-level *A*-PPFD and *A*-*C*_i_ responses under the four acute spectra are presented in the common order R100B0, R90B10, R50B50 and R0B100. The spectral measurement order was randomised. The values are the means ± SEMs. AUC_norm_ was calculated from eight complete plant curves per spectrum across shared measured domains. Fitted descriptors were compared using plant-blocked HC3 models. Each descriptor contains eight estimates per spectrum except *g*_m_ (*n* = 7, 8, 8 and 8). *R*_d,CO2_ is the paired Yin light-der_i_ved respiration estimate fixed in the finite-*g*_m_ CO_2_ model. TPU_op_ = median[(*A* + *R*_d,CO2_)/3] at measured *C*_i_ ≥ 800 µmol mol^−1^ and is expressed in triose-phosphate equivalents. The complete-curve responses and empirical descriptors support the spectral interpretation. Comparisons of *g*_m_, *C*_c_, *V*_cmax_, *J*_max_, Γ_CO2_ and *C*_i,90_ are retained as secondary descriptive summaries of the fitted coefficients; they do not establish biochemical differences or equivalence. Sections S5 and S11 document the parameter-specific identification and sensitivity underlying this distinction. The response curve principal component analysis (PCA) includes the 15 descriptors shown in Figure 3 and TPU_op_.

Endpoint (Unit)	R100B0	R90B10	R50B50	R0B100	Plant-Level Comparison
***A*-PPFD curve descriptors**					
*A*-PPFD AUC_norm_ (µmol CO_2_ m^−2^ s^−1^)	6.92 ± 1.35	8.23 ± 1.04	7.37 ± 0.98	6.14 ± 0.74	*F*_GG_(1.75, 12.23) = 4.092; *p* = 0.0479
*A*_p,max_ (µmol CO_2_ m^−2^ s^−1^)	13.19 ± 3.05	15.55 ± 2.72	14.40 ± 2.70	11.94 ± 2.04	HC3 *F*(3, 21) = 2.412; *p* = 0.0954
*A*_max_ (µmol CO_2_ m^−2^ s^−1^)	11.86 ± 2.92	14.09 ± 2.58	13.00 ± 2.59	10.63 ± 1.92	HC3 *F*(3, 21) = 2.303; *p* = 0.1065
*α* (mol CO_2_ mol^−1^ photons)	0.04351 ± 0.00197	0.04559 ± 0.00217	0.04200 ± 0.00225	0.03794 ± 0.00268	HC3 *F*(3, 21) = 4.825; *p* = 0.0104
*R*_d,light_ (µmol CO_2_ m^−2^ s^−1^)	1.33 ± 0.21	1.46 ± 0.20	1.40 ± 0.21	1.31 ± 0.20	HC3 *F*(3, 21) = 2.927; *p* = 0.0575
Light-compensation point (µmol photons m^−2^ s^−1^)	31.39 ± 3.47	32.39 ± 3.19	33.58 ± 3.38	35.37 ± 3.81	HC3 *F*(3, 21) = 1.930; *p* = 0.1556
PPFD at 75% modelled saturation (µmol photons m^−2^ s^−1^)	453.03 ± 105.50	515.32 ± 88.85	571.09 ± 161.75	541.17 ± 124.28	HC3 *F*(3, 21) = 0.216; *p* = 0.8842
*g*_sw_ at PPFD 400 (mol H_2_O m^−2^ s^−1^)	0.0973 ± 0.0249	0.1314 ± 0.0294	0.1189 ± 0.0270	0.0998 ± 0.0203	HC3 *F*(3, 21) = 1.712; *p* = 0.1951
***A*-*C*_i_ curve descriptors**					
*A*-*C*_i_ AUC_norm_ (µmol CO_2_ m^−2^ s^−1^)	21.97 ± 1.71	22.13 ± 1.64	19.55 ± 1.57	14.45 ± 1.43	*F*_GG_(1.28, 8.96) = 208.945; *p* < 0.001
*A*_max,CO2_ (µmol CO_2_ m^−2^ s^−1^)	24.97 ± 1.86	24.74 ± 1.77	21.84 ± 1.63	16.17 ± 1.36	HC3 *F*(3, 21) = 95.294; *p* < 0.001
Carboxylation efficiency, CE (mol m^−2^ s^−1^)	0.13684 ± 0.01454	0.14797 ± 0.01595	0.12649 ± 0.01428	0.08866 ± 0.01435	HC3 *F*(3, 21) = 16.112; *p* < 0.001
Γ_app_ (µmol CO_2_ mol^−1^)	20.40 ± 2.76	18.37 ± 6.02	19.71 ± 3.35	11.33 ± 6.69	HC3 *F*(3, 21) = 1.047; *p* = 0.3926
*g*_m_ (mol CO_2_ m^−2^ s^−1^)	0.351 ± 0.047	0.408 ± 0.066	0.346 ± 0.069	0.265 ± 0.071	HC3 *F*(3, 20) = 2.877; *p* = 0.0617
*C*_c_ (µmol CO_2_ mol^−1^)	168.10 ± 13.73	185.07 ± 16.50	173.91 ± 23.09	181.14 ± 28.38	HC3 *F*(3, 21) = 0.241; *p* = 0.8667
*R*_d,CO2_ (µmol CO_2_ m^−2^ s^−1^)	1.06 ± 0.27	1.28 ± 0.23	1.15 ± 0.21	0.97 ± 0.20	HC3 *F*(3, 21) = 3.953; *p* = 0.0222
*V*_cmax_ (µmol CO_2_ m^−2^ s^−1^)	235.67 ± 31.40	272.67 ± 30.04	259.39 ± 30.19	226.01 ± 41.71	HC3 *F*(3, 21) = 0.322; *p* = 0.8096
*J*_max_ (µmol e^−^ m^−2^ s^−1^)	205.60 ± 27.90	182.87 ± 15.24	264.64 ± 106.12	219.50 ± 111.92	HC3 *F*(3, 21) = 0.224; *p* = 0.8786
Operational TPU_op_ (µmol m^−2^ s^−1^)	8.53 ± 0.67	8.51 ± 0.63	7.49 ± 0.57	5.58 ± 0.47	HC3 *F*(3, 21) = 86.640; *p* < 0.001
Γ_CO2_ (µmol CO_2_ mol^−1^)	43.62 ± 0.82	44.15 ± 0.85	44.32 ± 0.97	45.16 ± 1.23	HC3 *F*(3, 21) = 1.410; *p* = 0.2679
*C*_i,90_ (µmol CO_2_ mol^−1^)	415.26 ± 63.50	362.02 ± 51.45	327.96 ± 16.48	410.97 ± 64.02	HC3 *F*(3, 21) = 0.814; *p* = 0.5005

**Table 2:** Assimilation amplitude, response time, stomatal and transpiration stabilisation, and integrated physiological costs during light induction and relaxation. Treatments are presented in the common order R100B0, R90B10, R50B50, and R0B100 within each *C*_a_ level.

*C*_a_ (µmol mol^−1^)	Spectrum	|Δ*A*| (µmol CO_2_ m^−2^ s^−1^)	*A t*_90_ (min)	*g*_sw_ *t*_90_ (min)	*E t*_90_ (min)	RT_stab_, % (*g*_sw_/*E*)	Integrated Cost
**Induction: PPFD 50 to 1000 µmol photons m^−2^ s^−1^; cost = carbon deficit, *C*_def_ (mmol CO_2_ m^−2^)**							
400	R100B0	11.52 ± 1.57	12.0 [11.2–15.0]	18.0 [14.6–24.7]	17.7 [14.5–24.6]	*g*_sw_: 68.9 [51.0–77.8] *E*: 67.6 [48.4–77.8]	3.881 ± 0.846
400	R90B10	14.68 ± 1.99	11.9 [10.5–18.6]	17.5 [16.3–27.5]	17.1 [16.0–27.3]	*g*_sw_: 73.7 [73.2–79.1] *E*: 73.7 [73.2–79.5]	4.639 ± 1.044
400	R50B50	8.14 ± 0.87	10.4 [6.5–10.5]	24.4 [14.5–25.8]	24.0 [14.5–25.2]	*g*_sw_: 69.2 [68.0–73.2] *E*: 68.8 [66.0–72.5]	1.163 ± 0.274
400	R0B100	5.63 ± 0.62	6.1 [3.0–9.5]	22.5 [13.4–27.8]	21.9 [13.4–26.7]	*g*_sw_: 76.9 [71.2–80.0] *E*: 75.6 [71.2–78.1]	0.575 ± 0.169
2000	R100B0	12.20 ± 2.67	By first measurement	28.7 [27.7–29.9]	27.5 [21.5–29.7]	*g*_sw_: 76.3 [71.9–81.0] *E*: 69.2 [66.7–78.9]	0.173 ± 0.101
2000	R90B10	16.19 ± 3.91	3.1 [0.5–7.2]	32.1 [31.7–32.4]	31.8 [31.3–32.2]	*g*_sw_: 79.2 [78.4–82.1] *E*: 78.9 [76.9–82.1]	1.896 ± 0.797
2000	R50B50	5.72 ± 0.89	By first measurement	29.6 [25.9–31.2]	28.0 [26.0–30.6]	*g*_sw_: 76.3 [65.4–79.0] *E*: 71.8 [69.0–77.0]	0.086 ± 0.024
2000	R0B100	5.21 ± 1.16	By first measurement	30.8 [30.0–31.3]	29.4 [27.1–30.3]	*g*_sw_: 79.5 [76.3–82.6] *E*: 76.6 [71.2–78.2]	0.059 ± 0.022
**Relaxation: PPFD 1000 to 50 µmol photons m^−2^ s^−1^; cost = water loss above the final low-light state, *W*_exc_ (mmol H_2_O m^−2^)**							
400	R100B0	11.67 ± 1.61	By first measurement	11.7 [10.7–12.9]	11.5 [10.4–12.4]	*g*_sw_: 63.2 [57.9–69.7] *E*: 63.2 [56.6–69.7]	449.4 ± 106.9
400	R90B10	15.00 ± 2.07	By first measurement	11.6 [8.6–14.7]	11.8 [8.4–14.3]	*g*_sw_: 63.2 [46.1–80.3] *E*: 63.2 [46.1–78.9]	560.2 ± 98.6
400	R50B50	8.24 ± 0.90	By first measurement	12.2 [11.4–14.7]	11.8 [11.1–14.2]	*g*_sw_: 63.2 [63.2–71.1] *E*: 63.2 [63.2–71.1]	474.4 ± 70.8
400	R0B100	5.68 ± 0.66	By first measurement	12.3 [11.0–13.7]	11.9 [10.7–13.2]	*g*_sw_: 68.4 [57.9–75.0] *E*: 65.8 [57.9–73.7]	458.3 ± 52.2
2000	R100B0	12.50 ± 2.77	By first measurement	15.0 [14.9–15.9]	13.4 [13.2–15.7]	*g*_sw_: 81.6 [77.6–84.2] *E*: 73.7 [72.4–76.3]	108.9 ± 37.2
2000	R90B10	16.72 ± 4.12	By first measurement	15.0 [14.5–15.3]	14.5 [14.3–15.2]	*g*_sw_: 78.9 [78.9–84.2] *E*: 78.9 [76.3–81.6]	230.9 ± 76.7
2000	R50B50	5.79 ± 0.89	By first measurement	14.3 [12.1–15.0]	11.5 [10.7–14.1]	*g*_sw_: 78.9 [65.8–78.9] *E*: 68.4 [61.8–79.0]	221.8 ± 53.4
2000	R0B100	5.42 ± 1.20	By first measurement	15.6 [15.4–16.2]	13.0 [10.9–14.7]	*g*_sw_: 84.2 [78.9–84.2] *E*: 63.2 [52.6–78.9]	131.0 ± 24.5

Eight plant trajectories were measured for each spectrum × *C*_a_ combination. |Δ*A*| and integrated costs are presented as the mean ± SEM of all eight trajectories (*n* = 8). *t*_90_ and RT_stab_ are medians [Q1-Q3] calculated from the eight plant trajectories in which the corresponding event was evaluated (*n* = 8). “By first measurement” indicates that the median threshold crossing occurred within the first 60–120 s post-transition observation interval.

**Table 3:** Plant-level repeated-measures comparisons for the signed baseline-corrected area (AUC_bc_) during induction and relaxation. Each cell reports the Greenhouse–Geisser-adjusted *F* test where applicable, nominal *p*, Benjamini–Hochberg family-adjusted *q*, and partial η_p_^2^. Spectrum and *C*_a_ were within-plant factors; *n* = 8 plants completed all eight spectrum × *C*_a_ conditions. A positive or negative AUC_bc_ retains the direction of change relative to the origin-phase baseline. These contrasts use the plant as the biological unit; individual sequential records are not additional replicates.

Response and Domain (AUC_bc_ Unit)	Spectrum	*C*_a_	Spectrum × *C*_a_
**Induction: PPFD 50 to 1000 µmol photons m^−2^ s^−1^**			
Carbon *A* (µmol CO_2_ m^−2^ s^−1^ min)	*F*(1.30, 9.08) = 26.65; *p* < 0.001; *q* < 0.001; η_p_^2^ = 0.792	*F*(1.00, 7.00) = 0.10; *p* = 0.765; *q* = 0.765; η_p_^2^ = 0.014	*F*(1.06, 7.41) = 1.36; *p* = 0.283; *q* = 0.283; η_p_^2^ = 0.162
Carbon *C*_i_ (µmol CO_2_ mol^−1^ min)	*F*(1.71, 11.97) = 38.35; *p* < 0.001; *q* < 0.001; η_p_^2^ = 0.846	*F*(1.00, 7.00) = 1.51; *p* = 0.258; *q* = 0.430; η_p_^2^ = 0.178	*F*(1.45, 10.15) = 6.01; *p* = 0.025; *q* = 0.081; η_p_^2^ = 0.462
Carbon Φ_CO2_ (mol CO_2_ mol^−1^ photons min)	*F*(1.68, 11.79) = 14.07; *p* = 0.001; *q* = 0.001; η_p_^2^ = 0.668	*F*(1.00, 7.00) = 2.19; *p* = 0.182; *q* = 0.275; η_p_^2^ = 0.239	*F*(1.84, 12.89) = 0.58; *p* = 0.563; *q* = 0.750; η_p_^2^ = 0.076
Water *g*_sw_ (mol H_2_O m^−2^ s^−1^ min)	*F*(1.98, 13.89) = 3.54; *p* = 0.057; *q* = 0.057; η_p_^2^ = 0.336	*F*(1.00, 7.00) = 17.71; *p* = 0.004; *q* = 0.020; η_p_^2^ = 0.717	*F*(2.37, 16.62) = 1.43; *p* = 0.269; *q* = 0.283; η_p_^2^ = 0.170
Water *E* (mmol H_2_O m^−2^ s^−1^ min)	*F*(1.94, 13.55) = 3.95; *p* = 0.046; *q* = 0.057; η_p_^2^ = 0.361	*F*(1.00, 7.00) = 10.39; *p* = 0.015; *q* = 0.036; η_p_^2^ = 0.598	*F*(2.54, 17.80) = 1.59; *p* = 0.230; *q* = 0.283; η_p_^2^ = 0.185
Water *i*WUE (µmol CO_2_ mol^−1^ H_2_O min)	*F*(1.64, 11.50) = 37.70; *p* < 0.001; *q* < 0.001; η_p_^2^ = 0.843	*F*(1.00, 7.00) = 1.02; *p* = 0.345; *q* = 0.432; η_p_^2^ = 0.128	*F*(1.42, 9.91) = 5.52; *p* = 0.032; *q* = 0.081; η_p_^2^ = 0.441
Microclimate VPD_leaf_ (kPa min)	*F*(1.76, 12.32) = 18.40; *p* < 0.001; *q* < 0.001; η_p_^2^ = 0.724	*F*(1.00, 7.00) = 1.90; *p* = 0.211; *q* = 0.211; η_p_^2^ = 0.213	*F*(2.04, 14.31) = 1.15; *p* = 0.344; *q* = 0.344; η_p_^2^ = 0.142
Microclimate *T*_leaf_ (°C min)	*F*(1.91, 13.35) = 51.86; *p* < 0.001; *q* < 0.001; η_p_^2^ = 0.881	*F*(1.00, 7.00) = 2.37; *p* = 0.168; *q* = 0.211; η_p_^2^ = 0.253	*F*(2.27, 15.89) = 1.42; *p* = 0.273; *q* = 0.344; η_p_^2^ = 0.168
PSII *F*_v_′/*F*_m_′ (dimensionless min)	*F*(1.60, 11.23) = 43.63; *p* < 0.001; *q* < 0.001; η_p_^2^ = 0.862	*F*(1.00, 7.00) = 0.27; *p* = 0.621; *q* = 0.621; η_p_^2^ = 0.037	*F*(1.32, 9.24) = 1.33; *p* = 0.293; *q* = 0.750; η_p_^2^ = 0.159
PSII Φ_PSII_ (dimensionless min)	*F*(1.50, 10.51) = 35.86; *p* < 0.001; *q* < 0.001; η_p_^2^ = 0.837	*F*(1.00, 7.00) = 1.94; *p* = 0.206; *q* = 0.275; η_p_^2^ = 0.217	*F*(1.15, 8.07) = 0.64; *p* = 0.470; *q* = 0.750; η_p_^2^ = 0.083
PSII ETR (µmol e^−^ m^−2^ s^−1^ min)	*F*(1.47, 10.30) = 20.15; *p* < 0.001; *q* < 0.001; η_p_^2^ = 0.742	*F*(1.00, 7.00) = 2.36; *p* = 0.168; *q* = 0.275; η_p_^2^ = 0.253	*F*(1.16, 8.15) = 0.04; *p* = 0.882; *q* = 0.882; η_p_^2^ = 0.005
Nonphotochemical quenching NPQ (dimensionless min)	*F*(1.28, 8.94) = 30.82; *p* < 0.001; *q* < 0.001; η_p_^2^ = 0.815	*F*(1.00, 7.00) = 0.78; *p* = 0.407; *q* = 0.542; η_p_^2^ = 0.100	*F*(1.16, 8.12) = 0.50; *p* = 0.524; *q* = 0.667; η_p_^2^ = 0.067
Nonphotochemical quenching *q*_N_ (dimensionless min)	*F*(1.44, 10.07) = 27.10; *p* < 0.001; *q* < 0.001; η_p_^2^ = 0.795	*F*(1.00, 7.00) = 0.09; *p* = 0.777; *q* = 0.777; η_p_^2^ = 0.012	*F*(1.16, 8.14) = 0.25; *p* = 0.667; *q* = 0.667; η_p_^2^ = 0.034
Reaction-centre openness *q*_P_ (dimensionless min)	*F*(1.43, 10.03) = 19.51; *p* < 0.001; *q* < 0.001; η_p_^2^ = 0.736	*F*(1.00, 7.00) = 2.30; *p* = 0.173; *q* = 0.398; η_p_^2^ = 0.247	*F*(1.13, 7.93) = 1.04; *p* = 0.350; *q* = 0.667; η_p_^2^ = 0.129
Reaction-centre openness *q*_L_ (dimensionless min)	*F*(1.38, 9.63) = 19.64; *p* < 0.001; *q* < 0.001; η_p_^2^ = 0.737	*F*(1.00, 7.00) = 2.01; *p* = 0.199; *q* = 0.398; η_p_^2^ = 0.223	*F*(1.32, 9.24) = 1.18; *p* = 0.325; *q* = 0.667; η_p_^2^ = 0.144
**Relaxation: PPFD 1000 to 50 µmol photons m^−2^ s^−1^**			
Carbon *A* (µmol CO_2_ m^−2^ s^−1^ min)	*F*(1.24, 8.70) = 32.94; *p* < 0.001; *q* < 0.001; η_p_^2^ = 0.825	*F*(1.00, 7.00) = 0.00; *p* = 0.968; *q* = 0.968; η_p_^2^ = 0.000	*F*(1.07, 7.50) = 0.75; *p* = 0.422; *q* = 0.422; η_p_^2^ = 0.097
Carbon *C*_i_ (µmol CO_2_ mol^−1^ min)	*F*(1.61, 11.26) = 54.84; *p* < 0.001; *q* < 0.001; η_p_^2^ = 0.887	*F*(1.00, 7.00) = 2.87; *p* = 0.134; *q* = 0.223; η_p_^2^ = 0.291	*F*(1.16, 8.13) = 6.74; *p* = 0.028; *q* = 0.087; η_p_^2^ = 0.491
Carbon Φ_CO2_ (mol CO_2_ mol^−1^ photons min)	*F*(1.97, 13.82) = 11.15; *p* = 0.001; *q* = 0.002; η_p_^2^ = 0.614	*F*(1.00, 7.00) = 4.19; *p* = 0.080; *q* = 0.210; η_p_^2^ = 0.374	*F*(1.97, 13.79) = 0.86; *p* = 0.445; *q* = 0.655; η_p_^2^ = 0.109
Water *g*_sw_ (mol H_2_O m^−2^ s^−1^ min)	*F*(1.87, 13.06) = 2.69; *p* = 0.108; *q* = 0.108; η_p_^2^ = 0.277	*F*(1.00, 7.00) = 51.77; *p* < 0.001; *q* < 0.001; η_p_^2^ = 0.881	*F*(2.18, 15.28) = 2.79; *p* = 0.089; *q* = 0.149; η_p_^2^ = 0.285
Water *E* (mmol H_2_O m^−2^ s^−1^ min)	*F*(1.97, 13.78) = 3.02; *p* = 0.082; *q* = 0.103; η_p_^2^ = 0.302	*F*(1.00, 7.00) = 27.21; *p* = 0.001; *q* = 0.003; η_p_^2^ = 0.795	*F*(2.21, 15.47) = 2.38; *p* = 0.121; *q* = 0.152; η_p_^2^ = 0.254
Water *i*WUE (µmol CO_2_ mol^−1^ H_2_O min)	*F*(1.48, 10.34) = 52.64; *p* < 0.001; *q* < 0.001; η_p_^2^ = 0.883	*F*(1.00, 7.00) = 2.12; *p* = 0.188; *q* = 0.235; η_p_^2^ = 0.233	*F*(1.14, 7.99) = 6.20; *p* = 0.035; *q* = 0.087; η_p_^2^ = 0.470
Microclimate VPD_leaf_ (kPa min)	*F*(1.99, 13.94) = 22.35; *p* < 0.001; *q* < 0.001; η_p_^2^ = 0.761	*F*(1.00, 7.00) = 2.15; *p* = 0.186; *q* = 0.186; η_p_^2^ = 0.235	*F*(1.76, 12.35) = 1.33; *p* = 0.295; *q* = 0.295; η_p_^2^ = 0.160
Microclimate *T*_leaf_ (°C min)	*F*(2.71, 18.97) = 49.09; *p* < 0.001; *q* < 0.001; η_p_^2^ = 0.875	*F*(1.00, 7.00) = 3.62; *p* = 0.099; *q* = 0.186; η_p_^2^ = 0.341	*F*(2.30, 16.08) = 1.41; *p* = 0.275; *q* = 0.295; η_p_^2^ = 0.167
PSII *F*_v_′/*F*_m_′ (dimensionless min)	*F*(2.03, 14.21) = 6.50; *p* = 0.010; *q* = 0.010; η_p_^2^ = 0.481	*F*(1.00, 7.00) = 0.64; *p* = 0.449; *q* = 0.449; η_p_^2^ = 0.084	*F*(1.71, 11.95) = 0.86; *p* = 0.432; *q* = 0.655; η_p_^2^ = 0.109
PSII Φ_PSII_ (dimensionless min)	*F*(1.28, 8.98) = 20.03; *p* = 0.001; *q* = 0.002; η_p_^2^ = 0.741	*F*(1.00, 7.00) = 2.95; *p* = 0.129; *q* = 0.210; η_p_^2^ = 0.297	*F*(1.17, 8.17) = 0.58; *p* = 0.492; *q* = 0.655; η_p_^2^ = 0.077
PSII ETR (µmol e^−^ m^−2^ s^−1^ min)	*F*(1.57, 11.02) = 22.97; *p* < 0.001; *q* < 0.001; η_p_^2^ = 0.766	*F*(1.00, 7.00) = 2.50; *p* = 0.158; *q* = 0.210; η_p_^2^ = 0.263	*F*(1.19, 8.30) = 0.09; *p* = 0.817; *q* = 0.817; η_p_^2^ = 0.012
Nonphotochemical quenching NPQ (dimensionless min)	*F*(1.17, 8.22) = 20.56; *p* = 0.001; *q* = 0.003; η_p_^2^ = 0.746	*F*(1.00, 7.00) = 5.90; *p* = 0.045; *q* = 0.182; η_p_^2^ = 0.457	*F*(1.16, 8.11) = 0.09; *p* = 0.812; *q* = 0.885; η_p_^2^ = 0.012
Nonphotochemical quenching *q*_N_ (dimensionless min)	*F*(1.34, 9.37) = 27.63; *p* < 0.001; *q* = 0.001; η_p_^2^ = 0.798	*F*(1.00, 7.00) = 0.01; *p* = 0.911; *q* = 0.911; η_p_^2^ = 0.002	*F*(1.10, 7.72) = 0.03; *p* = 0.885; *q* = 0.885; η_p_^2^ = 0.004
Reaction-centre openness *q*_P_ (dimensionless min)	*F*(1.32, 9.27) = 10.52; *p* = 0.007; *q* = 0.007; η_p_^2^ = 0.600	*F*(1.00, 7.00) = 2.86; *p* = 0.135; *q* = 0.269; η_p_^2^ = 0.290	*F*(1.14, 8.00) = 1.96; *p* = 0.202; *q* = 0.431; η_p_^2^ = 0.218
Reaction-centre openness *q*_L_ (dimensionless min)	*F*(1.32, 9.24) = 11.78; *p* = 0.005; *q* = 0.007; η_p_^2^ = 0.627	*F*(1.00, 7.00) = 1.81; *p* = 0.221; *q* = 0.294; η_p_^2^ = 0.205	*F*(1.24, 8.70) = 1.82; *p* = 0.216; *q* = 0.431; η_p_^2^ = 0.206

**Table 4:** Experimental design and effective gas exchange chamber conditions for rapid response curves and low–high–low kinetics. The spectral measurement order was randomised; the spectra are shown in a common presentation order. The two kinetic *C*_a_ concentrations were measured in separate sessions. Values in parentheses are observed ranges; kinetic ranges refer to complete-series means. Protocol-specific flow targets were maintained by automatic mass-flow control and were not spectral treatments.

Protocol	Design	Programmed Sequence	Reference CO_2_ and Chamber Flow	Effective Leaf Microclimate	Endpoints
Stepwise *A*-PPFD	8 plants × 4 spectra (32 curves)	PPFD 1000, 800, 600, 400, 250, 150, 100, 60, 30, 0.05	*C*_a_ 397.4 (388.9–401.0); flow 1200 µmol s^−1^	*T*_leaf_ 25.58 (24.66–27.69) °C; RH 65.20 (63.32–66.51)%; VPD_leaf_ 1.066 (0.953–1.267) kPa	AUC_norm_; light-response descriptors
Stepwise *A*-*C*_i_	8 plants × 4 spectra (32 curves)	*C*_a_ 400, 300, 200, 100, 50, 700, 1000, 1500, 2000	PPFD approximately 1000; flow predominantly 800 µmol s^−1^	*T*_leaf_ 26.89 (26.34–28.19) °C; RH 65.02 (64.63–67.20)%; VPD_leaf_ 1.190 (1.105–1.336) kPa	AUC_norm_; *A*_max,CO2_; FvCB curve descriptors
Kinetics, *C*_a_ 400	8 plants × 4 spectra (32 series)	PPFD 50 (15 min), 1000 (40 min), 50 (20 min)	*C*_a_ 396.66 (391.39–399.54); flow 925 ± 188 µmol s^−1^	*T*_leaf_ 26.57 (25.65–27.63) °C; RH 65.12 (64.93–65.26)%; VPD_leaf_ 1.157 (1.066–1.242) kPa	AUC_bc_; *t*_90_; RT_stab_; transition costs
Kinetics, *C*_a_ 2000	8 plants × 4 spectra (32 series)	PPFD 50 (15 min), 1000 (40 min), 50 (20 min)	*C*_a_ 1993.06 (1980.74–1998.06); flow 756 ± 17 µmol s^−1^	*T*_leaf_ 27.33 (26.65–28.42) °C; RH 65.04 (62.10–65.29)%; VPD_leaf_ 1.220 (1.152–1.336) kPa	AUC_bc_; *t*_90_; RT_stab_; transition costs

## Data Availability

The reported results and supporting analyses are included in the article and Supplementary Materials.

## Supplementary Materials

The following supporting information can be downloaded at: https://www.mdpi.com/article/10.3390/plants15203194/s1, Sections S1–S4, Tables S1–S4 and Figure S1 provide the primary response curve PCA specifications, eigenvalues, loadings, passive-variable projections, scores and sensitivity analyses. Section S5 and Table S5 provide the complete fitting methods, parameter uncertainty and sensitivity. Section S6 and Tables S6 and S7 describe analyser stability classifications and low-light states. Section S7 and Table S8 assess the operational high-*C*_i_ TPU proxy across respiration offsets and *C*_i_ thresholds. Section S8 and Figures S2–S4 present individual observed and fitted curves with residuals. Section S9 and Tables S9–S11 provide individual parameter estimates, conditional intervals and modelled electron transport at the measurement irradiance. Section S10 and Table S12 compare the primary 16-descriptor PCA with the 15-descriptor analysis. Section S11 and Figure S5 report the effects of fitting assumptions and upper-bound estimates on group summaries and PCA. Section S12 reports stability classifications by spectrum and acquisition step, curve coverage and observed-response sensitivity.

## Abbreviations

The following abbreviations and symbols are used in this manuscript.

**Abbreviation or Symbol**	**Definition**
*A*	Net CO_2_ assimilation rate
*A*-*C*_i_; *A*-PPFD	Assimilation responses to intercellular CO_2_ and photosynthetic photon flux density
*A*_c_; *A*_j_; *A*_p_	Rubisco-, electron-transport- and TPU-limited assimilation branches of the FvCB model
*A*_p,max_; *A*_max_	Gross and net assimilation asymptotes of the fitted light-response curve
*A*_max,CO2_	Empirical CO_2_-response assimilation plateau
ANOVA	Analysis of variance
AUC_norm_; AUC_bc_	Domain-normalised and signed baseline-corrected areas under the curve
*C*_a_; *C*_i_; *C*_c_	Reference, intercellular and model-derived chloroplastic CO_2_ mole fractions
*C*_i,90_	Intercellular CO_2_ mole fraction reaching 90% of the modelled CO_2_-response amplitude
*C*_def_; *W*_exc_	Integrated induction carbon deficit and postillumination excess water loss
CE	Empirical *A*-*C*_i_ slope at the fitted zero crossing Γ_app_
*E*	Transpiration rate
ETR	Apparent PSII electron-transport rate calculated from fluorescence
*F*_o_; *F*_m_; *F*_s_	Dark-adapted minimum fluorescence, dark-adapted maximum fluorescence and steady fluorescence in the light
*F*_o_′; *F*_m_′	Minimum and maximum light-adapted fluorescence
*F*_v_; *F*_v_/*F*_m_; *F*_v_′/*F*_m_′	Variable fluorescence, maximum dark-adapted PSII quantum efficiency and light-adapted potential PSII efficiency
FvCB	Farquhar–von Caemmerer–Berry photosynthesis model
GG; HC3	Greenhouse‒Geisser correction and type 3 heteroscedasticity-consistent covariance
*g*_m_; *g*_sw_; *g*_sc_	Mesophyll conductance to CO_2_ and stomatal conductance to water vapour and CO_2_
IRGA	Infrared gas analyser
iWUE; WUE	Intrinsic and instantaneous water-use efficiencies
*J*; *J*_max_	Model electron transport at the specified irradiance and maximum electron-transport parameter of its light response
*K*_c_; *K*_o_; *O*	Rubisco Michaelis‒Menten constants for CO_2_ and O_2_, and O_2_ concentration
*L*; τ	Response lag and first-order time constant
LCP; LSP; PPFD_75_	Fitted light-compensation point and operational light-saturation point at 75% response amplitude
LED	Light-emitting diode
MAD; SNR_amp_	Median absolute deviation and amplitude signal-to-noise ratio
NIPALS; PCA; PC	Nonlinear iterative partial least squares, principal component analysis and principal component
NPQ; *q*_N_	Nonphotochemical quenching and nonphotochemical quenching coefficient
PCE	Plant, Cell and Environment
PPFD; *Q*	Photosynthetic photon flux density
*P*_max_	Gross light-response assimilation asymptote, equivalent to *A*_p,max_
PSII	Photosystem II
*q*_P_; *q*_L_	Photochemical quenching coefficient and lake-model reaction-centre openness coefficient
*Q*_R_; *Q*_B_; *f*_R_; *f*_B_	Red and blue photon fluxes and their fractions of total programmed photon output
R100B0; R90B10; R50B50; R0B100	Red:blue photon recipes of 100:0, 90:10, 50:50 and 0:100
*R*_d_	Generic model respiration term, distinguished by its source or fitting role as stated
*R*_d,light_	Respiration term of the fitted nonrectangular light-response hyperbola
*R*_d,CO2_	Paired Yin light-derived respiration estimate used as the fixed respiration parameter in the finite-*g*_m_ CO_2_ model
RH; VPD_leaf_; *T*_leaf_	Relative humidity, leaf-to-air vapour-pressure deficit and leaf temperature
RT_stab_	Sustained stabilisation time as a percentage of the observed phase duration
*t*_10_; *t*_50_; *t*_63.2_; *t*_90_	Times to 10, 50, 63.2 and 90% of a transition amplitude
Rubisco	Ribulose-1,5-bisphosphate carboxylase/oxygenase
SEM; CI	Standard error of the mean and confidence interval
TPU	Triose-phosphate utilisation
TPU_op_	Operational high-*C*_i_ TPU proxy calculated from offset-corrected assimilation in triose-phosphate equivalents
*V*_cmax_	Maximum Rubisco carboxylation parameter in the FvCB model
*α*; *θ*	Initial apparent quantum yield and curvature parameter of the light-response curve
*α*_J_; *θ*_J_	Assumed initial slope and curvature of the model electron-transport light response
*α*_leaf_	Assumed leaf absorptance
Γ_app_; Γ_CO2_; Γ*	Empirical net zero crossing, model-derived net compensation point and photorespiratory compensation point without day respiration
Φ_PSII_; Φ_CO2_	Operating PSII quantum yield and apparent quantum yield of CO_2_ assimilation
η_p_^2^; *p*; *q*	Partial eta-squared, nominal *p* value and false-discovery-rate-adjusted *q* value
