# Supplementary material for: Integrating Rapid Light- and CO2-Response Curves with Low–High–Low Irradiance Kinetics Under Acute Red:Blue Spectra in Leaf Chicory
Source: Plants (Basel). 2026 Oct 18;15(20):3194. doi: 10.3390/plants15203194 (PMC13709034; doi:10.3390/plants15203194)
Supplement: Supplementary file 1 [file plants-15-03194-s001.zip › plants-4609598-supplementary.pdf]

Supplementary Materials

Integrating Rapid Light- and CO_2-Response Curves with Low–High–Low Irradiance Kinetics under Acute Red:Blue Spectra in Leaf Chicory

Marina Berg Von Linde[1], Carla Micaela Ripke Ferreira[1], Ellen Bruna Tacone[1], Ana Clara Verginio[1], Elora Berlato Cazela[1], Bruno Yoshikatsu Suguiura[1], João Paulo Alves da Silva[1], Werner Camargos Antunes[1], José A. M. Demattê[2], Marcos Rafael Nanni[1] and Renan Falcioni[1,*]

[1] Graduate Program in Agronomy, State University of Maringá, Avenida Colombo, 5790, Maringá 87020-900, Paraná, Brazil; pg407654@uem.br (M.B.V.L.); pg407664@uem.br (C.M.R.F.); pg407665@uem.br (E.B.T.); pg56666@uem.br (A.C.V.); pg407651@uem.br (E.B.C.); pg407650@uem.br (B.Y.S.); ra125202@uem.br (J.P.A.d.S.); wcantunes@uem.br (W.C.A.); mrnanni@uem.br (M.R.N.)

[2] Department of Soil Science, Luiz de Queiroz College of Agriculture, University of São Paulo, Avenida Pádua Dias, 11, Piracicaba 13418-260, São Paulo, Brazil; jamdemat@usp.br

* Correspondence: rfalcioni2@uem.br or renanfalcioni@gmail.com; Tel.: +55-44-3011-1359

Contents

Section S1. Response curve PCA data structure
Section S2. NIPALS PCA and component retention
Section S3. Supplementary variable projection
Section S4. Sensitivity of the retained PCA structure
Table S1. Eigenvalues and explained variance
Table S2. Active-variable correlation loadings
Table S3. Supplementary-variable correlations
Table S4. Curve-set scores for PC1–PC4
Figure S1. Response curve PCA correlation loadings
Section S5. Response curve fitting and parameter uncertainty
Section S6. Analyser stability and low-light states
Section S7. Operational high-C_i TPU proxy and PCA sensitivity
Tables S5–S8. Parameter intervals and supporting sensitivity analyses
Section S8 and Figures S2–S4. Individual curves and residuals
Section S9 and Tables S9–S11. Individual parameter estimates and uncertainty
Section S10 and Table S12. Comparison of the 16-descriptor and 15-descriptor PCA
Section S11, Tables S13–S14 and Figure S5. Fitted-coefficient and PCA influence analyses
Section S12 and Tables S15–S17. Stability classifications, coverage and observed-response sensitivity

Section S1. Response curve PCA data structure

Response curve principal component analysis was conducted at the curve-set level using 32 plant × spectrum observations (8 plants × 4 acute spectra). The active matrix contained the 15 physiological descriptors displayed in Figure 3 plus the operational high-C_i triose-phosphate utilisation proxy (TPU_{op}), yielding 16 active variables. Thirty-one curve sets had complete values for all 16 variables. The g_m cell for R100B0 plant 3 was left missing because its estimate reached the upper bound. No value was imputed.

Section S2. NIPALS PCA and component retention

Active variables were centred and scaled to unit variance using their available observations. PCA was performed by nonlinear iterative partial least squares (NIPALS), with least-squares updates and matrix deflation calculated only from observed cells. Thus, the component scores for R100B0 plant 3 were calculated from its 15 observed active variables without estimating or replacing the missing g_m value. Explained variance was calculated from the sequential reduction in observed standardised sums of squares and expressed on the 16-variable scale. Four components had eigenvalues of at least 1 and were retained; together, PC1–PC4 explained 90.08% of the response curve variance. The component signs are arbitrary.

Section S3. Supplementary variable projection

The carboxylation efficiency (CE), normalised *A*-PPFD area under the curve, and normalised A-C_i area under the curve were treated as passive supplementary variables. They were projected after PCA fitting by calculating Pearson correlations with the final NIPALS component scores. These variables did not participate in scaling, component extraction, score calculation, eigenvalue estimation, or component retention.

Section S4. Sensitivity of the retained PCA structure

The four-component structure was consistent with the complementary treatments of the single missing cell. Relative to a complete-case analysis of 31 curves, the canonical correlations between the first four NIPALS score subspaces were 0.99998, 0.99995, 0.99987, and 0.99673. Winsorising active variables at the 2.5th and 97.5th percentiles changed the retained four-component loading subspace by 2.18°. Among the 31-curve sensitivities, the largest change was 7.46° for the set comprising R0B100 plants 1–6 and 8 and all eight plants under the other spectra; R0B100 plant 7 had the largest V_{cmax} and J_{max} estimates. The four retained components should therefore be interpreted jointly, without assigning excessive importance to any single component. The complementary analysis of the 15 Figure 3 descriptors retained four components, which explained 89.78% of the variance, compared with 90.08% in the complete descriptor set. The canonical correlations between the retained score subspaces were 1.00000, 0.99999, 0.99975 and 0.98995. The primary PCA is retained as a joint summary of the reported curve descriptors; the 15-descriptor analysis is presented in Sections S7 and S10.

Table S1. Eigenvalues and explained variance for the NIPALS PCA of 32 plant × spectrum curve sets. The active matrix contained the 15 Figure 3 descriptors plus operational TPU_{op}. Components with eigenvalues of at least 1 were retained.

Component	Eigenvalue	Variance (%)	Cumulative (%)	Retained
PC1	7.4248	46.4053	46.4053	Yes
PC2	2.9898	18.6865	65.0918	Yes
PC3	2.5359	15.8495	80.9413	Yes
PC4	1.4623	9.1396	90.0809	Yes
PC5	0.5719	3.5742	93.6551	No
PC6	0.3378	2.1112	95.7663	No
PC7	0.2462	1.5388	97.3051	No

Component	Eigenvalue	Variance (%)	Cumulative (%)	Retained
PC8	0.1697	1.0603	98.3654	No
PC9	0.1041	0.6504	99.0158	No
PC10	0.0654	0.4088	99.4246	No
PC11	0.0562	0.3510	99.7756	No
PC12	0.0260	0.1625	99.9381	No
PC13	0.0065	0.0409	99.9790	No
PC14	0.0020	0.0124	99.9914	No
PC15	0.0008	0.0051	99.9965	No
PC16	0.0004	0.0026	99.9991	No

The small difference between the final cumulative value and 100% reflects the residual observed-cell sum of squares after NIPALS deflation and numerical precision.

Table S2. Correlation loadings of the 16 active response curve variables on the four retained NIPALS components. The values are Pearson correlations between standardised active variables and component scores.

Active variable	PC1	PC2	PC3	PC4
$A_{p,max}$	0.9346	−0.0503	−0.1542	0.2382
A_{max}	0.9185	−0.0141	−0.1751	0.2746
α	0.7656	−0.1803	0.3768	−0.3169
$R_{d,light}$	0.7772	−0.4873	0.1684	−0.3117
Light-compensation point	0.6423	−0.6032	0.0172	−0.2995
PPFD at 75% modelled saturation	0.7204	0.0904	−0.2925	0.4380
g_{sw} at PPFD 400	0.8895	−0.0264	−0.1721	0.3056
g_m	0.5804	0.7273	−0.2203	−0.1492
C_c	0.5509	0.5065	−0.5736	−0.0394
$A_{max,CO2}$	0.4913	0.6513	0.3750	−0.4046
$R_{d,CO2}$	0.9194	−0.3195	0.0384	−0.1700
V_{cmax}	0.3880	−0.0310	0.7659	0.3946
J_{max}	0.1189	−0.2562	0.8396	0.3283
Γ_{CO2}	0.4661	−0.6487	−0.5110	−0.1802
$C_{i,90}$	−0.6034	−0.4050	−0.1295	−0.2902
Operational TPU_{op}	0.5642	0.5998	0.3492	−0.4140

The correlation loadings for g_m used 31 available observations; the component scores and all the other active-variable correlations used 32 curve sets. The component signs follow those in Figure 6 and Figure S1 but are mathematically arbitrary.

Table S3. Correlations of the passive supplementary variables with the four retained response curve PCA scores. Supplementary variables were projected after fitting and did not define the PCA axes.

Supplementary variable	PC1	PC2	PC3	PC4
A-PPFD AUC_{norm}	0.9137	0.0330	−0.0910	0.1835
A-C_i AUC_{norm}	0.5507	0.6566	0.3578	−0.3413
CE	0.6855	0.6618	0.2022	−0.1618

Table S4. Curve-set scores for the four retained response curve components. Each row represents one plant × spectrum curve set.

Spectrum	Plant	PC1	PC2	PC3	PC4
R100B0	1	0.1456	1.6116	−0.3891	0.6546
R100B0	2	−4.0163	0.4487	2.1687	1.0289
R100B0	3	−3.9963	−1.4069	−1.6152	−1.4515
R100B0	4	0.3441	2.3008	0.5713	0.2749
R100B0	5	−1.7969	0.8853	0.9040	−1.5740
R100B0	6	0.8032	0.3826	1.5728	−2.7122
R100B0	7	2.3468	−0.0186	0.2044	−0.2323
R100B0	8	4.6446	−0.9947	−0.4096	−0.3214
R90B10	1	1.4096	1.3329	0.1611	1.4526
R90B10	2	−3.4131	0.7096	0.6601	0.3046
R90B10	3	−2.3390	−2.3129	−1.0800	−0.7495
R90B10	4	1.2248	2.6531	0.8441	−0.1652
R90B10	5	0.1027	2.2742	0.1811	−0.4480
R90B10	6	1.3753	0.5398	1.4574	−2.4884
R90B10	7	2.7273	−0.3277	0.0439	−0.5963
R90B10	8	5.6986	−0.9975	−0.1745	0.2716
R50B50	1	0.3505	1.7182	−1.4161	1.4720
R50B50	2	−4.1733	0.0894	0.9367	0.8195
R50B50	3	−2.1669	−2.0501	−1.2973	−0.1547
R50B50	4	−0.7615	2.2581	0.0505	−0.7250
R50B50	5	2.0555	1.5234	−0.6487	1.5031
R50B50	6	0.8668	0.0191	0.8034	−2.2055
R50B50	7	1.1094	−2.0173	4.0896	1.4721
R50B50	8	4.7523	−1.5955	−1.3217	0.1425
R0B100	1	−1.7388	1.5398	−1.9971	1.2383
R0B100	2	−5.3080	−1.5148	−0.3616	0.1150
R0B100	3	−3.0754	−3.2460	−2.0571	0.0565
R0B100	4	−0.8895	1.6971	−1.1342	0.4619

Spectrum	Plant	PC1	PC2	PC3	PC4
R0B100	5	0.0374	0.9039	−2.1578	1.2817
R0B100	6	0.0112	−0.9219	−0.5172	−1.2458
R0B100	7	0.0487	−3.0927	4.3393	1.9812
R0B100	8	3.4328	−3.0206	−2.2734	0.6315

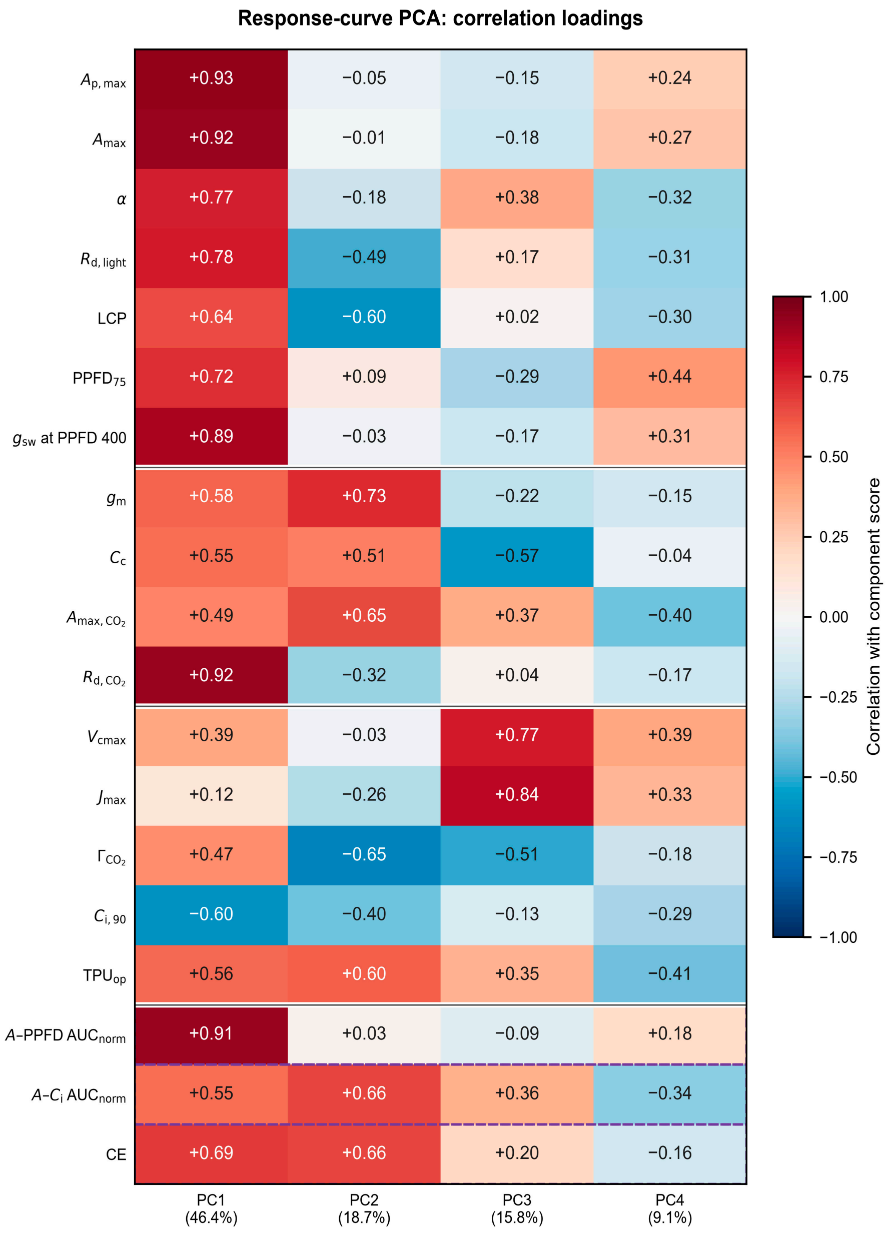

Figure S1. Response curve PCA correlation loadings for physiological variables. Cell colours and printed values show Pearson correlations between each variable and the component scores for PC1–PC4; red and blue denote positive and negative correlations, respectively. The percentages beneath the component labels indicate the variance explained by each component. The first 16 rows are active variables, and the final three rows are supplementary curve summaries projected after fitting. Together, PC1–PC4 explained 90.08% of the response curve variance. The component signs are

arbitrary. The fitted descriptors share observations and model assumptions; the loadings describe covariance rather than independent biochemical mechanisms.

Section S5. Response curve models, parameter interpretation and sensitivity

S5.1. Model purpose and observations

The empirical and FvCB models describe complementary aspects of the photosynthetic response. The empirical models summarise the magnitude, initial slope and saturation pattern of each curve. The FvCB framework provides a common C_3 representation that incorporates mesophyll CO_2 transfer, Rubisco carboxylation, electron transport and triose-phosphate utilisation. Its fitted quantities are retained as secondary descriptive summaries under the same parameterisation. Their nominal comparisons do not establish independently identified biochemical capacities; the spectral interpretation relies on complete observed responses and empirical descriptors. Branch coverage and the sensitivity analyses in Sections S5.7 and S11 determine the scope of parameter interpretation.

The models used 32 plant × spectrum A–PPFD curves with ten observations per curve and 32 corresponding A–C_i curves with nine observations per curve. The spectral measurement order was randomised. The within-curve steps were physiological observations from one enclosed leaf area; the plant was the biological unit. Instrument stability classifications were retained under a flagging policy: A–C_i included 63 observations classified as 2/2, 156 as 1/2 and 69 as 0/2; the corresponding light curve counts were 242, 56 and 22, respectively. The fitting procedure required finite A and response-axis values and, for FvCB, positive C_i. All ten light and nine CO_2 observations remained available in each respective curve. The models represent responses obtained by the specified rapid protocol; instrument classifications alone do not establish physiological equilibrium. Section S12 presents the classifications by spectrum and acquisition step and compares the complete profiles with classification-defined subsets.

S5.2. Empirical response models

The nonrectangular hyperbola was used for the light curves.

$$A(Q) = \frac{\alpha Q + P_{\max} - \sqrt{(\alpha Q + P_{\max})^2 - 4\theta\alpha Q P_{\max}}}{2\theta} - R_{d,light}$$

The bounds were 0.00001–0.5 for α, 0.05–300 μmol CO_2 m^{-2} s^{-1} for $P_{\max}$, 0.01–0.999 for θ and 0–30 μmol CO_2 m^{-2} s^{-1} for $R_{d,light}$. $A_{p,\max} = P_{\max}$ and $A_{\max} = P_{\max} - R_{d,light}$. LCP is the root $A(Q) = 0$. $PPFD_{75}$ completes 75% of the modelled response amplitude above $A(Q = 0)$; thus, its net assimilation target is 0.75Pmax − $R_{d,light}$. Stomatal conductance at PPFD 400 was interpolated from the measured g_{sw}. $R_{d,light}$ is the apparent offset of the rapid light-response curve and can include transient postillumination CO_2 release.

The empirical CO_2 curve was

$$A = A_{\max,CO2}\left[1 - \exp\left\{-\frac{CE(C_i - \Gamma_{app})}{A_{\max,CO2}}\right\}\right]$$

Bounds were 0.05–500 μmol CO_2 m^{-2} s^{-1} for $A_{\max,CO2}$, 0.00001–5 mol m^{-2} s^{-1} for CE and −500 to 1000 μmol mol^{-1} for Γ_{app}. CE is dA/dCi at $C_i = \Gamma_{app}$, with units (μmol CO_2 m^{-2} s^{-1})/(μmol CO_2 mol^{-1}), which is equivalent to mol m^{-2} s^{-1}. Γ_{app} is the fitted empirical zero crossing; it was below the lowest measured C_i in all 32 curves and is therefore extrapolated. It is distinct from Γ^*, the compensation point without respiration.

Initial CO_2 estimates came from a linear regression of the three lowest-C_i observations. CE_0 was the slope clipped to 0.001–1. $\Gamma_{app,0}$ was −intercept/slope for a positive slope; otherwise, it was 40 μmol mol^{-1} and was clipped to −200 to 300. The initial $A_{max,CO2}$ was the maximum observed A clipped to 0.5–200. Light-model initial values used the slope of the three lowest-PPFD observations, clipped to 0.001–0.20; the negative median of the near-dark A, clipped to 0.01–10; $P_{max,0}$ = maximum A + initial respiration, clipped to 0.2–200; and $\theta_0 = 0.70$.

S5.3. Respiration used in the CO_2 model

$R_{d,CO2}$ denotes the paired Yin light-derived respiration estimate used as a fixed offset in the finite-g_m CO_2 model. It was obtained by ordinary least-squares regression $A = b_0 + b_1(\Phi_{PSII} \times \mathrm{PPFD}/4)$, with $R_{d,CO2} = -b_0$, using finite observations with $0 < \mathrm{PPFD} \leq 200$ μmol photons m^{-2} s^{-1}. Each of the 32 regressions contained four observations at approximately 30, 60, 100 and 150 μmol photons m^{-2} s^{-1}; the observed range was 29.9624–150.0216. Eligible regressions required at least three points, $b_0 \leq 0$ and $b_1 > 0$. All the fitted offsets ranged from 0–20 μmol CO_2 m^{-2} s^{-1}. Each CO_2 curve used its own plant × spectrum estimate.

This offset differs from that of $R_{d,light}$ fitted by the light hyperbola. It was treated as the reference-temperature respiration parameter of the corresponding A–C_i curve and scaled to its individual observations. No additional correction was applied between the source light-curve mean temperature and the A–C_i reference temperature. The respiration estimate is thus conditional on the light-response regression and rapid acquisition conditions.

S5.4. FvCB formulation and derived quantities

The finite-g_m model fit V_{cmax}, J_{max}, TPU and g_m, with respective bounds of 1–600 μmol CO_2 m^{-2} s^{-1}, 1–1000 μmol e$^-$ m^{-2} s^{-1}, 0.05–100 μmol triose-P m^{-2} s^{-1} and 0.005–5 mol CO_2 m^{-2} s$^{-1, \text{respectively}}$. $R_{d,CO2}$ was fixed. J_{max} was fitted directly and converted to J at each measured PPFD Q by

$$J(Q) = \frac{\alpha_J Q + J_{\max} - \sqrt{\left(\alpha_J Q + J_{\max}\right)^2 - 4\theta_J \alpha_J Q J_{\max}}}{2\theta_J}$$

with $\alpha_J = 0.30$ and $\theta_J = 0.70$. These coefficients were fixed across spectra and were not separately calibrated for each spectrum. This modelled J is distinct from the apparent fluorescence-derived ETR. The TPU expression used $\alpha_g = 0$, yielding a net TPU-limited rate of 3TPU − $R_{d,CO2}$.

Finite conductance A_c and A_j were calculated analytically. For capacity ξ, denominator constant κ, compensation point Γ^*, respiration R and g_m, define $B = R - g_m(C_i + \kappa) - \xi$ and $D = g_m[\xi(C_i - \Gamma^*) - R(C_i + \kappa)]$. The smaller quadratic root is $A = [-B - (B^2 - 4D)^{1/2}]/2$. For A_c, $\xi = V_{cmax}$ and $\kappa = K_c(1 + O/K_o)$; for A_j, $\xi = J/4$ and $\kappa = 2\Gamma^*$. These analytical branch rates are already net rates. The assimilation prediction is the minimum with 3TPU − R, with no further subtraction of respiration. Very small negative discriminants were clipped to zero; a discriminant below -10^{-8} returned a nonfinite branch rate.

At each point, $C_c = C_i - A_{model}/g_m$, using fitted net A and temperature-adjusted g_m. The ambient descriptors used the measured C_a nearest 399.01 μmol mol^{-1}, without interpolation to the nominal C_a 400. The R100B0 mean g_m summarises the seven non-boundary estimates ($n = 7$); plant 3's upper-bound estimate is reported individually, and its associated C_c remains in the eight-plant C_c summary. Γ_{CO2} is the model-derived net compensation point $A_{model}(C_i) = 0$ at the mean measured temperature, PPFD and pressure and includes respiration.

The separate apparent-C_i FvCB-J_{max} solution set $C_c = C_i$ and fitted V_{cmax}, J_{max}, TPU and respiration freely, with bounds 1–600, 1–1000, 0.05–100 and 0–20 in their respective units. It supplied $C_{i,90}$, the first C_i completing 90% of the modelled response amplitude above $A(C_i = 0)$. Its plateau was the maximum predicted A on a 2500-point grid extending to the larger of 5000 μmol mol^{-1} and three times the maximum observed C_i; root crossings were refined with Brent's method.

TPU_{op} = median[$(A + R_{d,CO2})/3$] for measured $C_i \geq 800$ μmol mol^{-1}. This operational high-C_i proxy is calculated from observed assimilation and the paired Yin offset; it is distinct from the TPU fitted jointly by the FvCB. The one-third scaling expresses assimilation on the TPU stoichiometric scale. TPU_{op} does not independently establish a biochemical TPU limitation or directly measure end-product synthesis.

S5.5. Temperature and kinetic constants

The models used measured temperature and pressure at every A–C_i observation. The pressure ranged from 95.5081 to 95.8629 kPa; the curve-mean reference temperatures ranged from 26.4143 to 27.4829 °C. The reported capacities refer to the mean temperature of each curve. The pointwise scaling used $f(T, T_{ref}, E_a) = \exp[(E_a/R_{gas})(1/T_{ref} - 1/T)]$, with absolute temperatures in kelvin and $R_{gas} = 8.31446261815324$ J mol^{-1} K^{-1}.

The finite-g_m model used the tobacco-based Bernacchi (2002)/Sharkey (2007) C_c constants: $K_{c,25} = 27.238$ Pa, $K_{o,25} = 16.582$ kPa and $\Gamma^*25 = 3.743$ Pa, with activation energies of 80.990, 23.720 and 24.460 kJ mol$^{-1, \text{ respectively}}$. O_2 was 21% (210 mmol mol^{-1}). K_c and Γ^* in Pa were converted to μmol mol^{-1} as 1000 × partial pressure/atmospheric pressure in kPa; K_o in kPa was converted to mmol mol^{-1} as 1000 × K_o/atmospheric pressure. Γ^* was additionally scaled by the O_2 partial pressure relative to 21.0 kPa. The activation energies for V_{cmax}, J_{max}, TPU, respiration and g_m were 65.33, 43.90, 53.10, 46.39 and 49.60 kJ mol$^{-1, \text{ respectively}}$.

The apparent-C_i model used Bernacchi (2001): $K_{c,25} = 404.9$ μmol mol^{-1}, $K_{o,25} = 278.4$ mmol mol^{-1} and $\Gamma^*25 = 42.75$ μmol mol^{-1}, with activation energies of 79.43, 36.38 and 37.83 kJ mol$^{-1, \text{ respectively}}$. K_c and K_o were multiplied by 100.0 kPa/measured pressure, and Γ^* was scaled by O_2 relative to 210 mmol mol^{-1}. The activation energies for V_{cmax}, J_{max}, TPU and respiration were 65.33, 43.54, 53.10 and 46.39 kJ mol^{-1}, respectively. The transfer of these kinetic constants to chicory was an assumption, not a species-specific calibration.

S5.6. Optimisation and uncertainty

Bounded fits used scipy.optimize.least_squares with the trust-region-reflective algorithm, soft_l1 loss, x_scale = jac and a maximum of 5000 function evaluations. The loss scale was max[median{|A − median(A)|}, 0.1] μmol CO_2 m^{-2} s^{-1}. Five initial vectors were used: the algorithmic starting estimate and four multiplicative exp[Normal(0,0.35)] perturbations. Starting vectors were clipped inside the bounds with a margin of max[10^{-9} × bound span, 10^{-12}]. The converged solution with the lowest unweighted residual sum of squares was retained. No parameter bootstrap was used for these fits.

R_{d0} = max(0.5, −minimum A), clipped to 0.05–8, is used for finite-g_m initialisation. The initial V_{cmax} was the median positive one-point inversion at the three lowest C_i observations using the apparent C_i constants and R_{d0}, clipped to 5–300. J_0 = 4[75th percentile(A) + R_{d0}], clipped to 10–500; $J_{max,0}$ = max(J_0,50). TPU_0 = [maximum A + R_{d0}]/3, clipped to 0.5–50, and g_{m0} = 0.20. R_{d0} was used only to generate starting values; the paired Yin offset was fixed during the finite-g_m fit.

Local covariance was approximated by pinv($Jrob^T J_{rob}$) × RSS/(n − k), where J_{rob} is the optimised Jacobian modified for robust loss and k is the number of free parameters. Intervals were estimated ± $t_{0.975,n-k}$ × SE. They are conditional on the fixed offset, kinetic constants, optical coefficients and curve protocol. An inactive Jacobian direction or bound-

constrained parameter can prevent an informative local interval; such cases are indicated as unavailable, including zero-width intervals arising from the pseudoinverse.

Jacobian columns were normalised before the condition number and singular-value rank were assessed, with the rank threshold max[10^{-5} × largest singular value, machine epsilon]. Values within the first or last 1% of a permitted range were flagged as near a bound. Global numerical identification requires $n \geq k + 2$, a finite normalised condition number below 10^{10} and no bound flag. Physiological branch coverage was assessed separately. The group confidence intervals are presented as the mean ± $t_{0.975,n-1}$ × between-plant SEM; the paired intervals represent available within-plant differences. These intervals describe the variation among curve-level estimates and do not incorporate all within-curve model uncertainty or establish equivalence.

S5.7. Branch coverage and sensitivity results

Thirty of the 32 finite-g_m solutions assigned no observation to A_c; one assigned a point to A_c at a numerical tie with A_j near $C_c = \Gamma^*$, and the remaining solution had several A_c points with g_m at its upper bound. V_{cmax} had a numerically inactive Jacobian column in 28 solutions (column norm below 10^{-7}; 27 were exactly zero); two J_{max} estimates reached the upper bound, and one curve met the global numerical identification criterion. Parameter-specific sensitivity and physiological coverage therefore complement the whole-model criterion. These findings explain why individual FvCB quantities can have less resolving power than the complete empirical response: branch coverage and coupling among parameters affect which capacities the observations constrain. The means and nominal plant-blocked comparisons are retained for descriptive completeness, but they are not used as tests of identified biochemical capacities. Absence of an A_c-limited branch particularly weakens V_{cmax} estimation, while the observed response profile and its empirical descriptors remain available for comparison. Section S11 examines the consequences for J_{max} group summaries and multivariate interpretation.

There were 33 positive-A observations at C_i below the apparent-C_i Γ^* in 30 curves and 31 observations below the corresponding C_c-set Γ^*. The mean observed-minus-fitted residuals at C_a concentrations of 100 and 50 μmol mol^{-1} were +2.35 and +2.76 μmol CO_2 m^{-2} s$^{-1, \text{respectively}}$. This low-$C_i$ mismatch indicates that the rapid observations and the assumed equilibrium kinetic representation are not fully concordant; the available data do not isolate its cause. Relative to the nominal three-start sensitivity fit, increasing the J_{max} upper bound from 1000 to 2000 changed individual estimates by up to 1000, while the greatest change in the RMSE was 0.00484 μmol CO_2 m^{-2} s^{-1}. Relative to that same control, changing α_J from 0.30 to 0.20 changed J_{max} by up to 866, with a maximum change in the RMSE of 0.0409 μmol CO_2 m^{-2} s^{-1}. Additional initial vectors and respiration-offset sensitivities likewise showed parameter coupling. These results inform interpretation of the fitted capacities without changing the observed response profiles or their reported plant-level contrasts. Individual observed and fitted curves with residuals are presented in Section S8; individual parameter estimates and conditional intervals are presented in Section S9.

Table S5. Between-plant 95% confidence intervals for fitted FvCB descriptors. Units are mol CO_2 m^{-2} s^{-1} for g_m, μmol CO_2 mol^{-1} for C_c, μmol CO_2 m^{-2} s^{-1} for V_{cmax} and μmol e$^-$ m^{-2} s^{-1} for J_{max}. Intervals describe dispersion among curve-level estimates under the stated model and do not include within-curve fitting uncertainty.

Descriptor	Spectrum	*n*	Mean	95% CI
g_m	R100B0	7	0.351	0.236 to 0.466
g_m	R90B10	8	0.408	0.253 to 0.563
g_m	R50B50	8	0.346	0.184 to 0.509

Descriptor	Spectrum	*n*	Mean	95% CI
g_m	R0B100	8	0.265	0.098 to 0.432
C_c	R100B0	8	168.097	135.624 to 200.570
C_c	R90B10	8	185.072	146.045 to 224.100
C_c	R50B50	8	173.906	119.310 to 228.502
C_c	R0B100	8	181.140	114.021 to 248.260
V_{cmax}	R100B0	8	235.674	161.422 to 309.925
V_{cmax}	R90B10	8	272.673	201.645 to 343.702
V_{cmax}	R50B50	8	259.387	187.993 to 330.782
V_{cmax}	R0B100	8	226.008	127.377 to 324.640
J_{max}	R100B0	8	205.602	139.635 to 271.569
J_{max}	R90B10	8	182.875	146.835 to 218.915
J_{max}	R50B50	8	264.642	13.702 to 515.582
J_{max}	R0B100	8	219.498	−45.143 to 484.139

Intervals use the sample mean ± the two-sided 95% Student's t critical value × SEM, with $n - 1$ degrees of freedom. A negative lower endpoint for a constrained positive parameter reflects the breadth of between-plant dispersion. The local within-curve intervals are presented in Tables S9–S10. Intervals that cannot be estimated meaningfully for inactive coefficients are identified, and the effects of parameter coupling and bounds are explained.

Section S6. Analyser stability and low-light states

Measurements spanned 10 d, with the response curves acquired before the kinetic measurements. The response data comprise 320 light-response and 288 CO_2-response observations from 32 plant × spectrum curves per protocol and 64 plant × spectrum × C_a kinetic trajectories. The spectral measurement order was randomised. The complete curve or trajectory, rather than an individual sequential record, was the unit contributed by each plant under a treatment condition.

Table S6. Instrument stability classifications retained with rapid response curve observations. All observations and their recorded stability classifications were retained. *A* classification of 2/2 indicates that both configured criteria were met; 1/2 and 0/2 indicate one or neither criterion, respectively. These analyser classifications are distinct from those of full physiological equilibration.

Protocol	Records	State 2/2	State 1/2	State 0/2
A-PPFD	320	242	56	22
A-C_i	288	63	156	69

The averaging setting was 4 s. This is distinct from the stability evaluation window, which was not specified in the exported settings. The step durations and differential-signal criteria are given in Section 4.3. Complete curves were compared under the common rapid program, retaining the recorded classifications under the stated flagging policy. Extended holds would compare rapid responses with fully equilibrated curves, whereas return-to-ambient or reverse sequences would assess dependence on the preceding CO_2 history. These additional protocols address equilibration or history dependence; the present analyses describe responses under the acquisition program actually applied.

Low-light states were characterised from the initial 15 min and final 20 min phases of each 75 min trajectory, separated by 40 min of high light. As in the main analysis, the final 20% of each phase, with at least three observations, defined the tail. Range and

absolute ordinary-least-squares slope were scaled to the induction amplitude; these are descriptive summaries of the recorded trajectories.

Table S7. Initial low-light tail variation and final-minus-initial low-light states. The values are medians across the 32 plant-level trajectories at each C_a. The tail range and slope are expressed relative to the induction amplitude of the same trajectory.

C_a (μmol mol^{-1})	Variable	Tail range (% amplitude)	Absolute slope (% amplitude min^{-1})	Final minus initial (response units)
400	A	0.398	0.131	−0.108 μmol CO_2 m^{-2} s^{-1}
400	g_{sw}	3.193	1.156	+0.00362 mol H_2O m^{-2} s^{-1}
400	E	3.024	1.069	+0.0350 mmol H_2O m^{-2} s^{-1}
2000	A	1.227	0.310	−0.1104 μmol CO_2 m^{-2} s^{-1}
2000	g_{sw}	7.900	3.426	+0.00971 mol H_2O m^{-2} s^{-1}
2000	E	5.846	2.278	+0.1221 mmol H_2O m^{-2} s^{-1}

The initial assimilation-tail ranges were less than 10% of the induction amplitude in all 64 trajectories. The median ranges were 0.40% and 1.23% at C_a 400 and 2000, respectively. The water-flux variables showed greater relative variation, particularly where their induction amplitudes were small. These results describe the observed starting states and the variable-dependent relaxation after the high-light phase at the same reference C_a.

Section S7. Operational high-C_i TPU proxy and PCA sensitivity

The operational proxy TPU_{op} = median[(A + $R_{d,CO2}$)/3] for measured $C_i \geq 800$ μmol mol^{-1} expresses the respiration-corrected high-C_i assimilation flux in triose-phosphate equivalents. It preserves the original values and scaling. $R_{d,CO2}$ is the paired Yin light-derived estimate fixed in the finite-g_m fit, which is distinct from $R_{d,light}$ fitted by the nonrectangular hyperbola. The proxy does not independently identify end-product utilisation capacity or TPU limitation [48,77].

We varied the C_i threshold (600, 800 and 1000 μmol mol^{-1}) and respiration offset (zero, $R_{d,\ light}$ and $R_{d,\ CO2}$), retaining one estimate per plant and spectrum. Two or three observations entered each median at thresholds 600 and 800 and one or two at 1000. R0B100 remained the lowest, and R50B50 remained intermediate in all nine calculations. For R100B0 versus R90B10, the zero-offset calculation at threshold 1000 gave a Holm-adjusted p = 0.0440; the other eight comparisons did not reach 0.05. Thus, the broad spectral ranking was stable, whereas the red-rich pair was more sensitive to the calculation. The correlations with empirical $A_{max,CO2}$ ranged from 0.9947–0.9993; the proxy and plateau are related summaries of the same high-C_i assimilation response.

Table S8. Sensitivity of the operational TPU proxy to the C_i threshold and respiration offset. Means are in μmol m^{-2} s^{-1} of triose-phosphate equivalents, with eight plant-level estimates per spectrum.

C_i threshold (μmol mol^{-1})	Offset	R100B0	R90B10	R50B50	R0B100
600	$R_{d,CO2}$	8.518	8.526	7.530	5.582
600	$R_{d,light}$	8.610	8.584	7.615	5.695
600	Zero	8.166	8.098	7.148	5.258
800	$R_{d,CO2}$	8.532	8.509	7.487	5.576
800	$R_{d,light}$	8.624	8.567	7.572	5.689
800	Zero	8.180	8.081	7.104	5.252
1000	$R_{d,CO2}$	8.527	8.493	7.437	5.440

C_i threshold (μmol mol^{-1})	Offset	R100B0	R90B10	R50B50	R0B100
1000	$R_{d,light}$	8.619	8.551	7.522	5.553
1000	Zero	8.175	8.065	7.055	5.115

The complementary PCA of the 15 Figure 3 descriptors retained four components that explained 89.78% of the variance, compared with 90.08% in the 16-descriptor analysis. The canonical correlations between their four-component score subspaces were 1.00000, 0.99999, 0.99975 and 0.98995. This close agreement indicates that the broad multivariate structure is not determined by the inclusion of the proxy. The primary PCA is retained because its purpose is to integrate the reported light response, CO_2 response and fitted physiological descriptors; shared observations, algebraic relationships and fitting assumptions characterise the interpretation of the axes. The variance explained and the agreement between the retained score subspaces are summarised in Section S10 and Table S12.

Section S8. Individual response curves and residuals

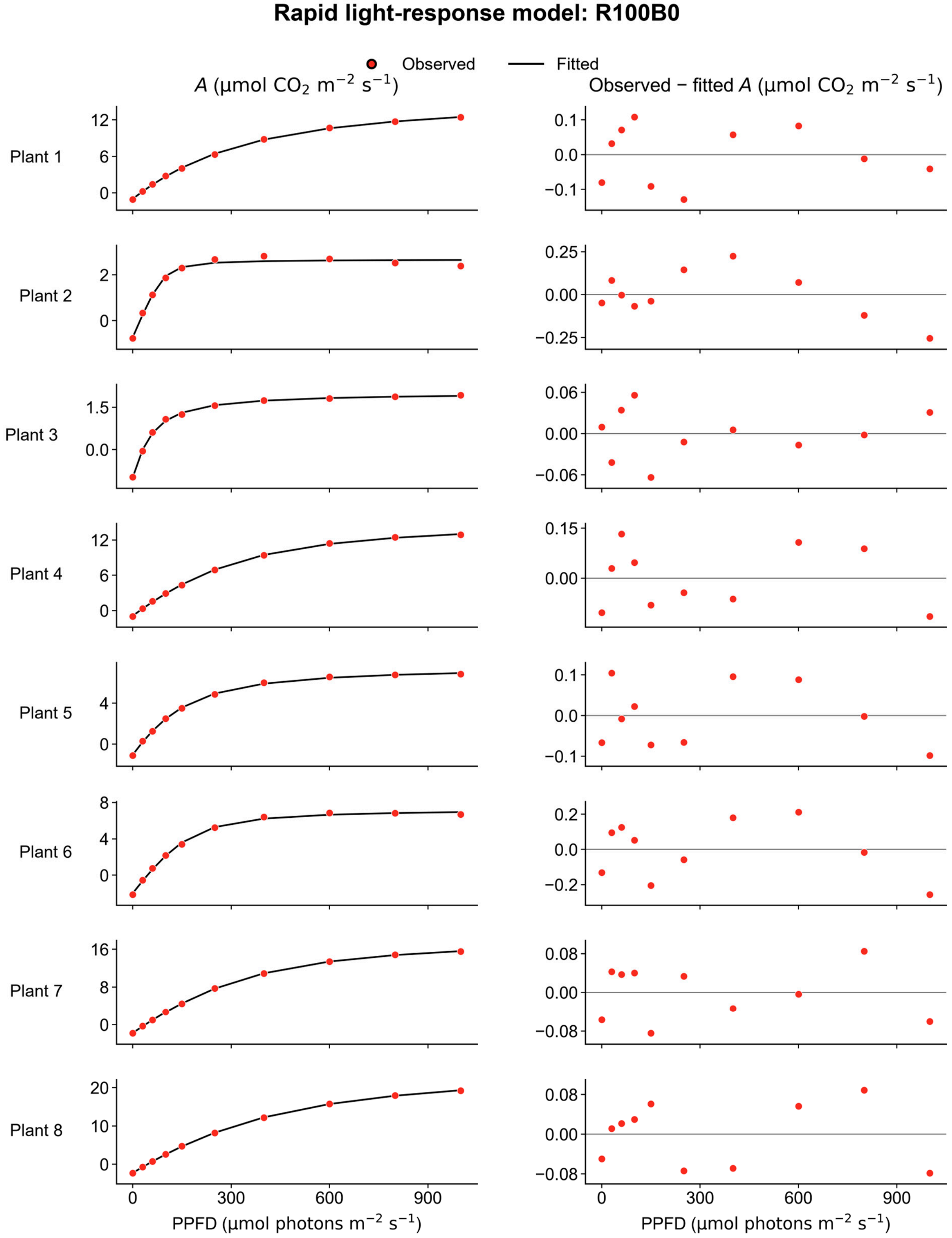

Figure S2. Individual curves fitted with the nonrectangular light-response hyperbola: R100B0, plants 1–8. Left: observed net assimilation (coloured points) and fitted values (black lines); right: observed-minus-fit residuals. Each plant has ten retained observations. The four spectral pages show all 32 curves. Axis ranges vary between plants.

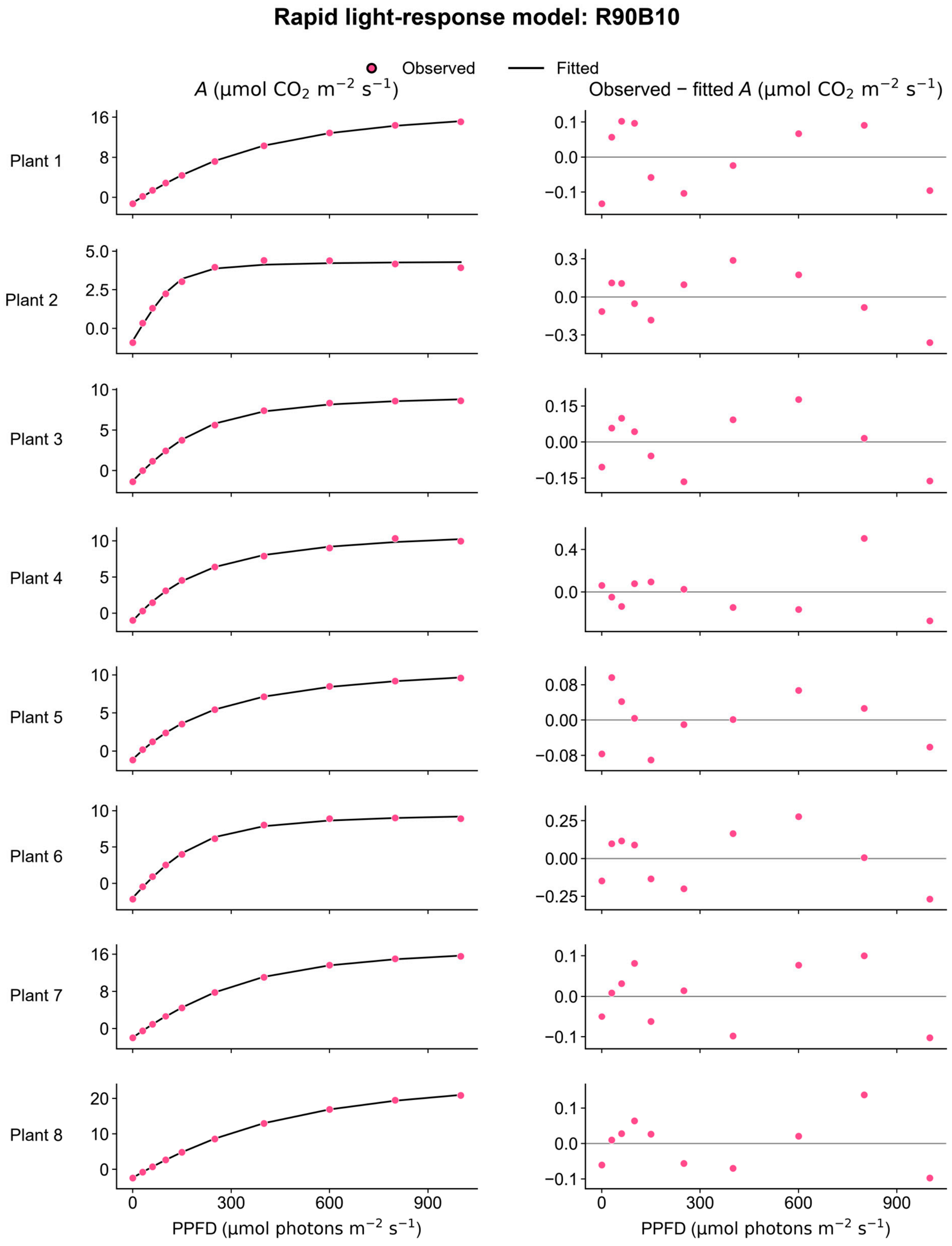

Figure S2. (Continued.) Individual curves fitted with the nonrectangular light-response hyperbola: R90B10, plants 1–8. Left: observed net assimilation (coloured points) and fitted values (black lines); right: observed-minus-fit residuals. Each plant has ten retained observations. The four spectral pages show all 32 curves. Axis ranges vary between plants.

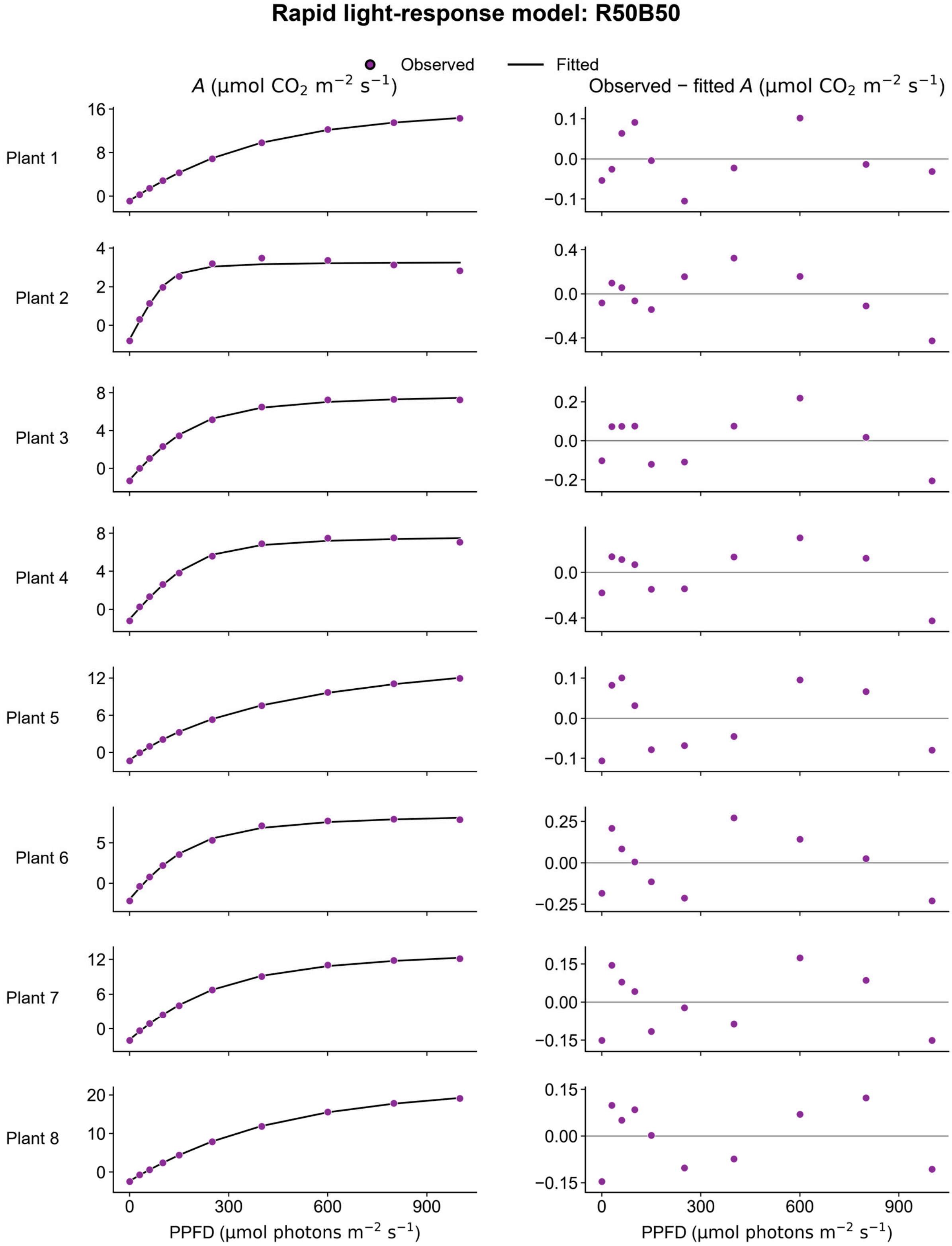

Figure S2. (Continued.) Individual curves fitted with the nonrectangular light-response hyperbola: R50B50, plants 1–8. Left: observed net assimilation (coloured points) and fitted values (black lines); right: observed-minus-fit residuals. Each plant has ten retained observations. The four spectral pages show all 32 curves. Axis ranges vary between plants.

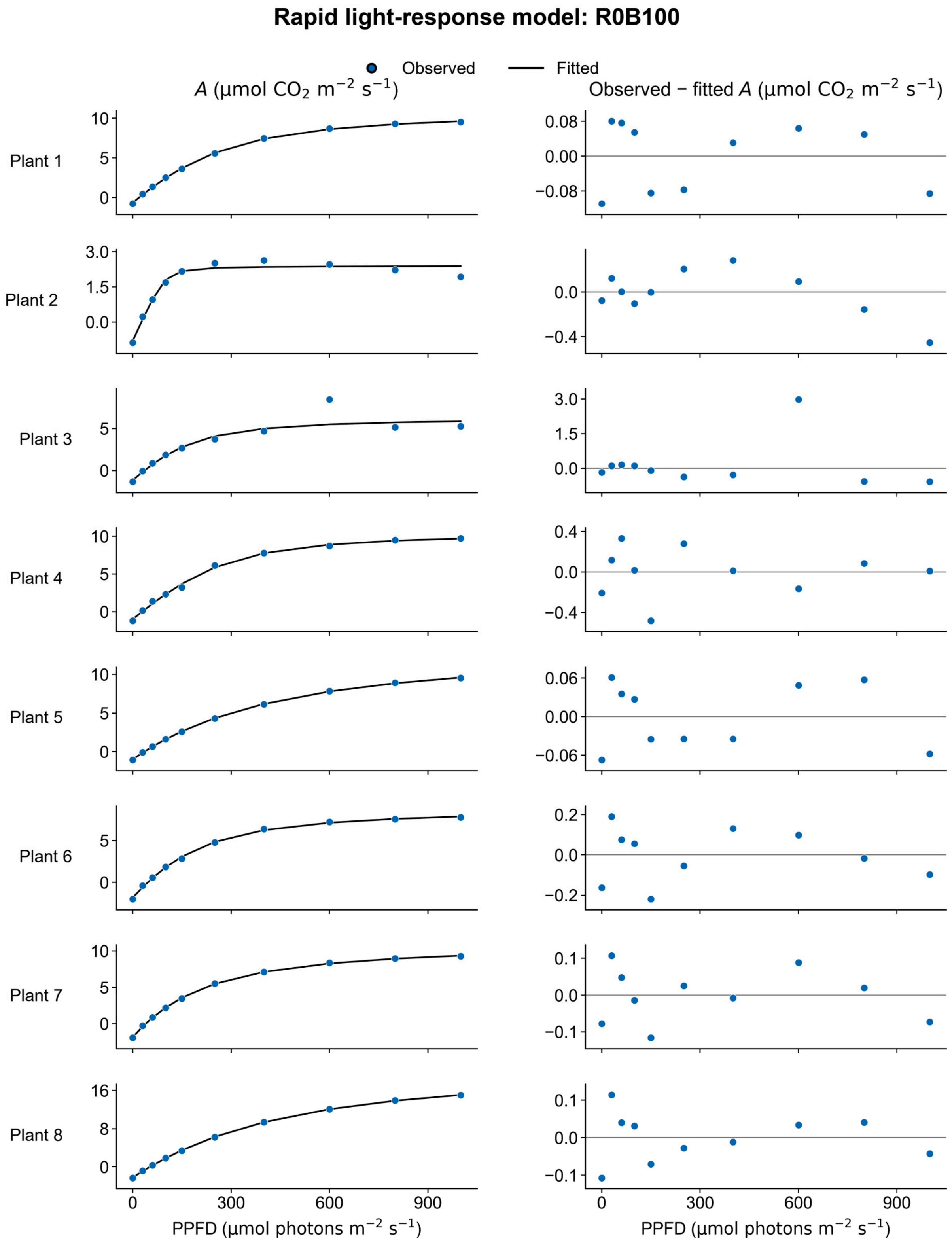

Figure S2. (Continued.) Individual curves fitted with the nonrectangular light-response hyperbola: R0B100, plants 1–8. Left: observed net assimilation (coloured points) and fitted values (black lines); right: observed-minus-fit residuals. Each plant has ten retained observations. The four spectral pages show all 32 curves. Axis ranges vary between plants.

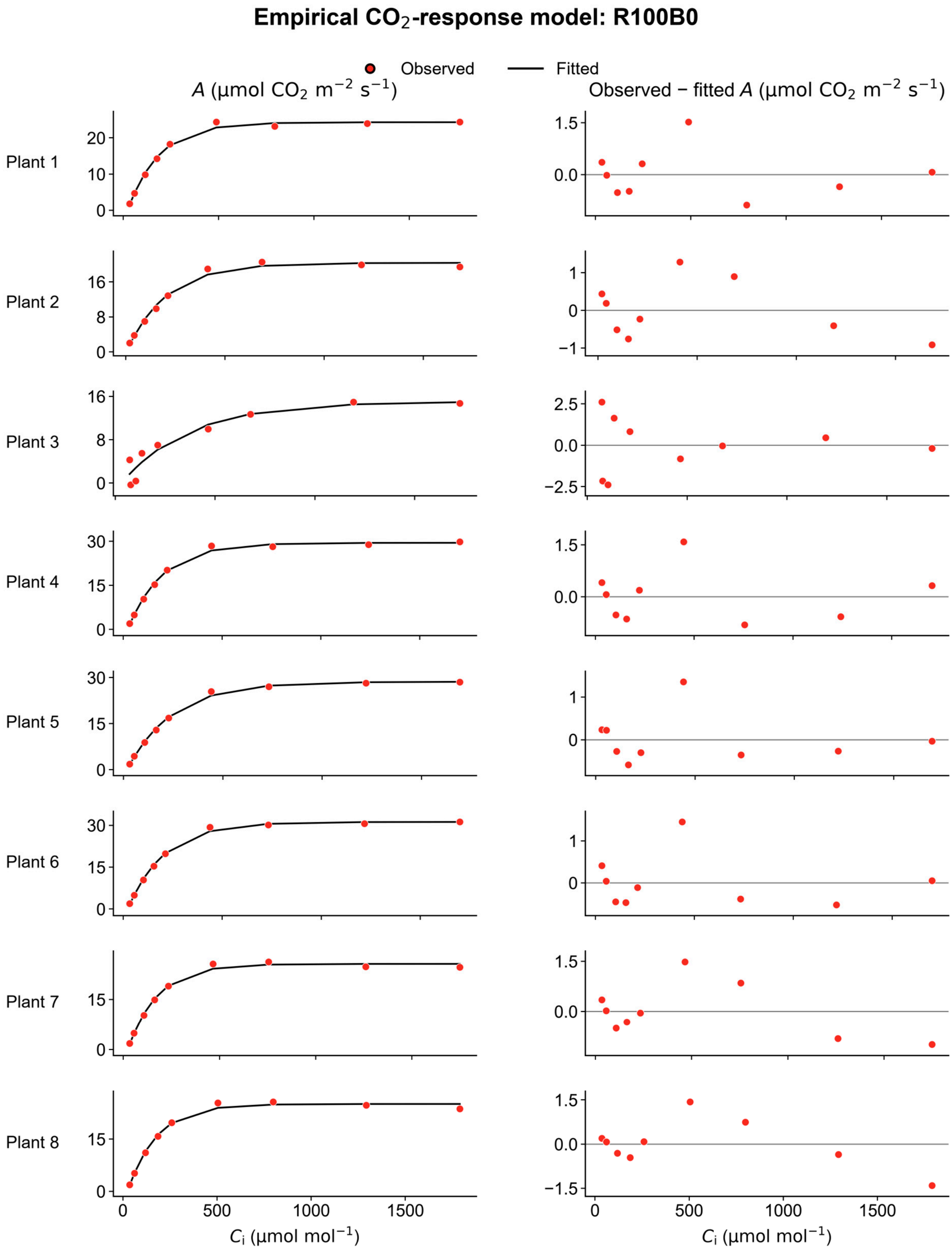

Figure S3. Individual curves fitted with the empirical CO_2-response exponential: R100B0, plants 1–8. Left: observed net assimilation (coloured points) and fitted values (black lines); right: observed-minus-fit residuals. Each plant has nine retained observations. The four spectral pages show all 32 curves. Axis ranges vary between plants.

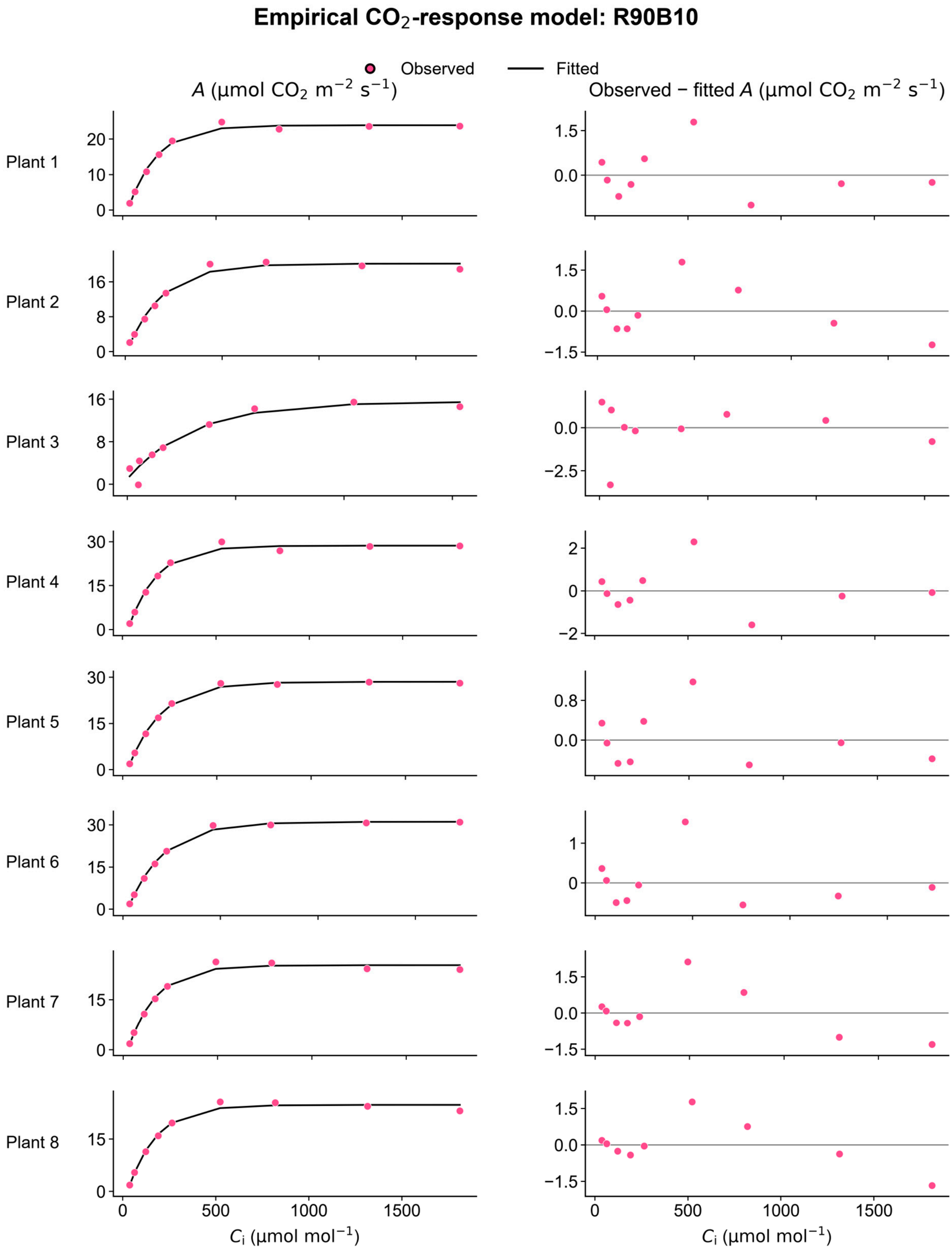

Figure S3. (Continued.) Individual curves fitted with the empirical CO_2-response exponential: R90B10, plants 1–8. Left: observed net assimilation (coloured points) and fitted values (black lines); right: observed-minus-fit residuals. Each plant has nine retained observations. The four spectral pages show all 32 curves. Axis ranges vary between plants.

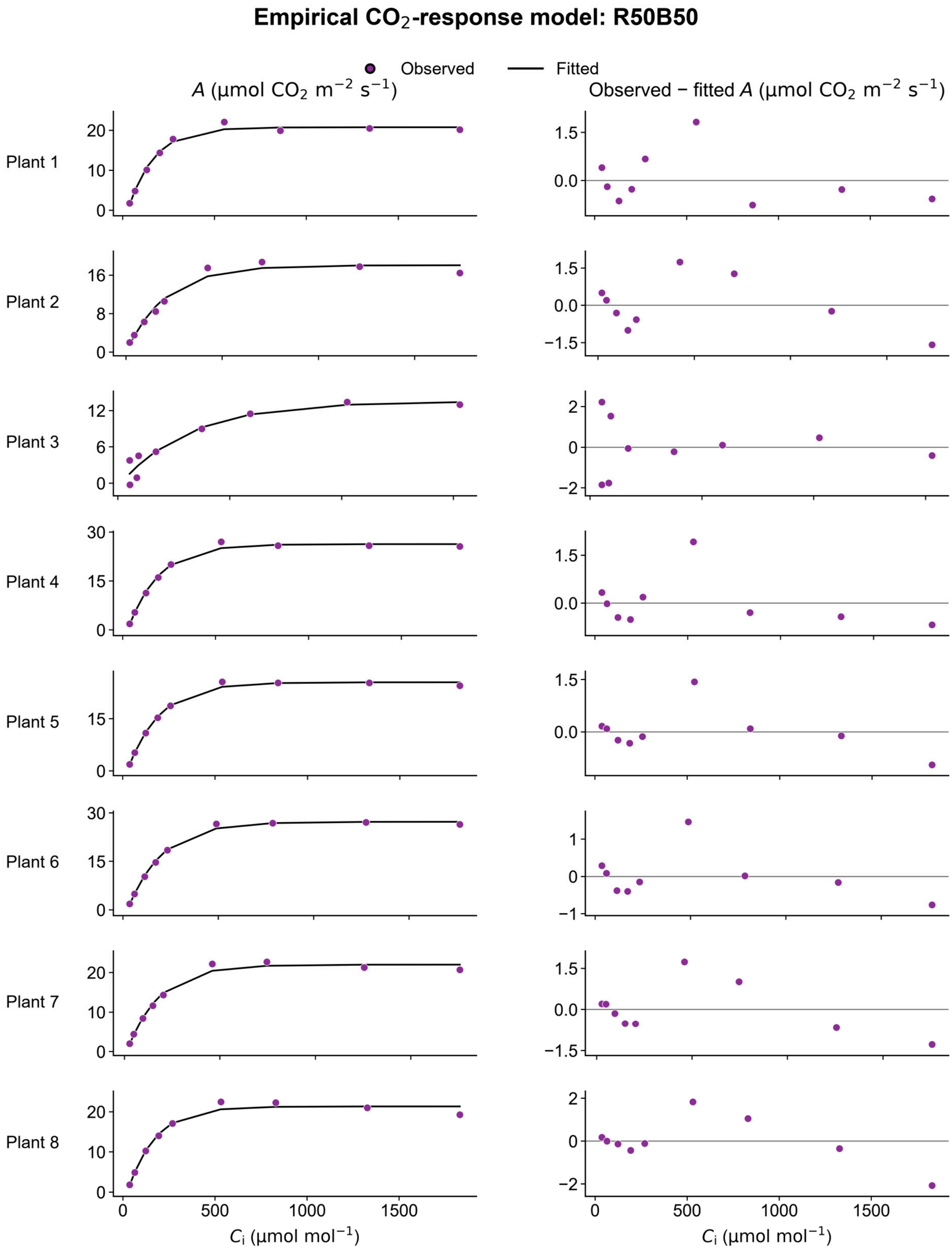

Figure S3. (Continued.) Individual curves fitted with the empirical CO_2-response exponential: R50B50, plants 1–8. Left: observed net assimilation (coloured points) and fitted values (black lines); right: observed-minus-fit residuals. Each plant has nine retained observations. The four spectral pages show all 32 curves. Axis ranges vary between plants.

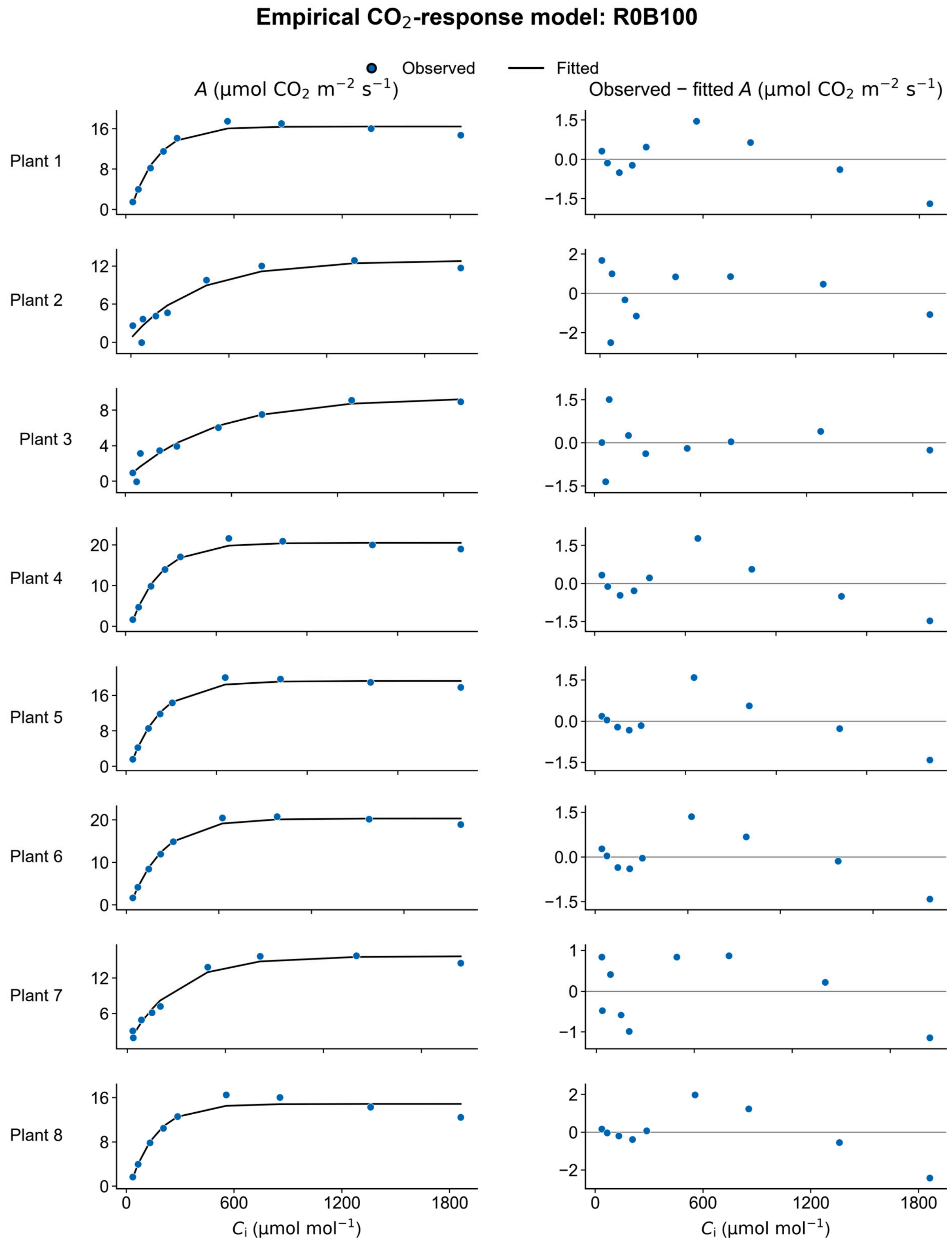

Figure S3. (Continued.) Individual curves fitted with the empirical CO_2-response exponential: R0B100, plants 1–8. Left: observed net assimilation (coloured points) and fitted values (black lines); right: observed-minus-fit residuals. Each plant has nine retained observations. The four spectral pages show all 32 curves. Axis ranges vary between plants.

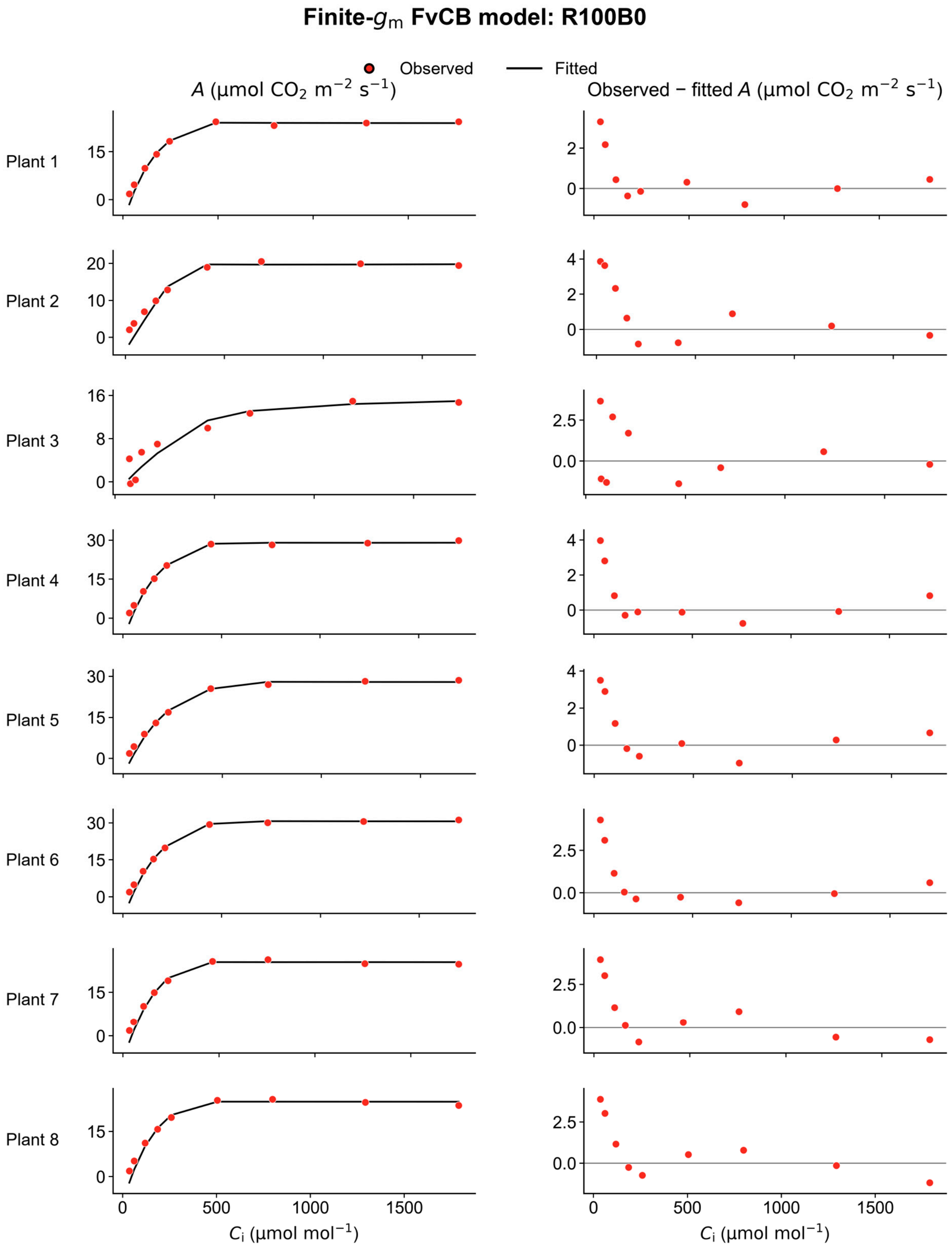

Figure S4. Individual curves fitted with the finite-g_m FvCB model with paired Yin $R_{d,CO2}$: R100B0, plants 1–8. Left: observed net assimilation (coloured points) and fitted values (black lines); right: observed-minus-fit residuals. Each plant has nine retained observations. The four spectral pages show all 32 curves. Axis ranges vary between plants.

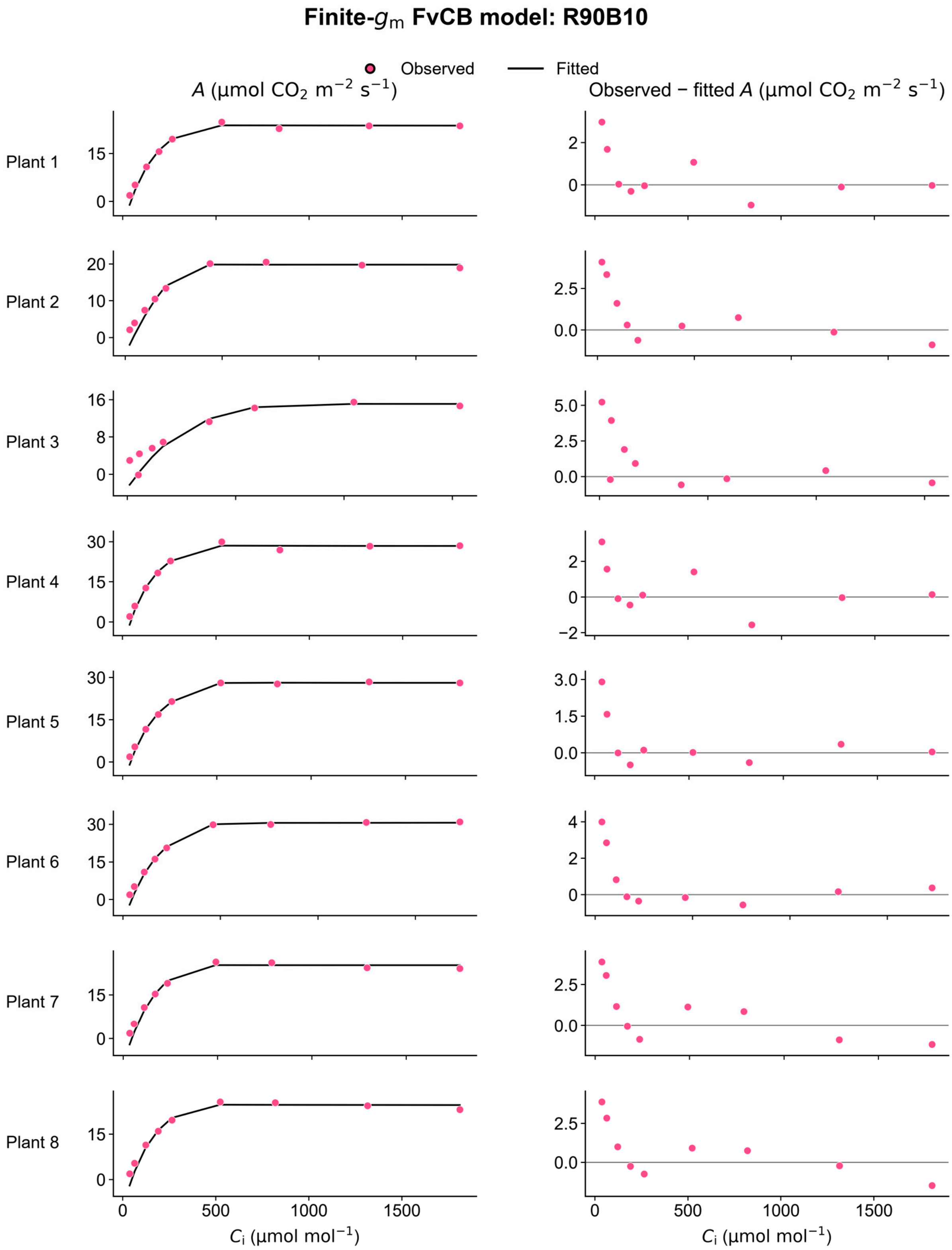

Figure S4. (Continued.) Individual curves fitted with the finite-g_m FvCB model with paired Yin $R_{d,CO2}$: R90B10, plants 1–8. Left: observed net assimilation (coloured points) and fitted values (black lines); right: observed-minus-fit residuals. Each plant has nine retained observations. The four spectral pages show all 32 curves. Axis ranges vary between plants.

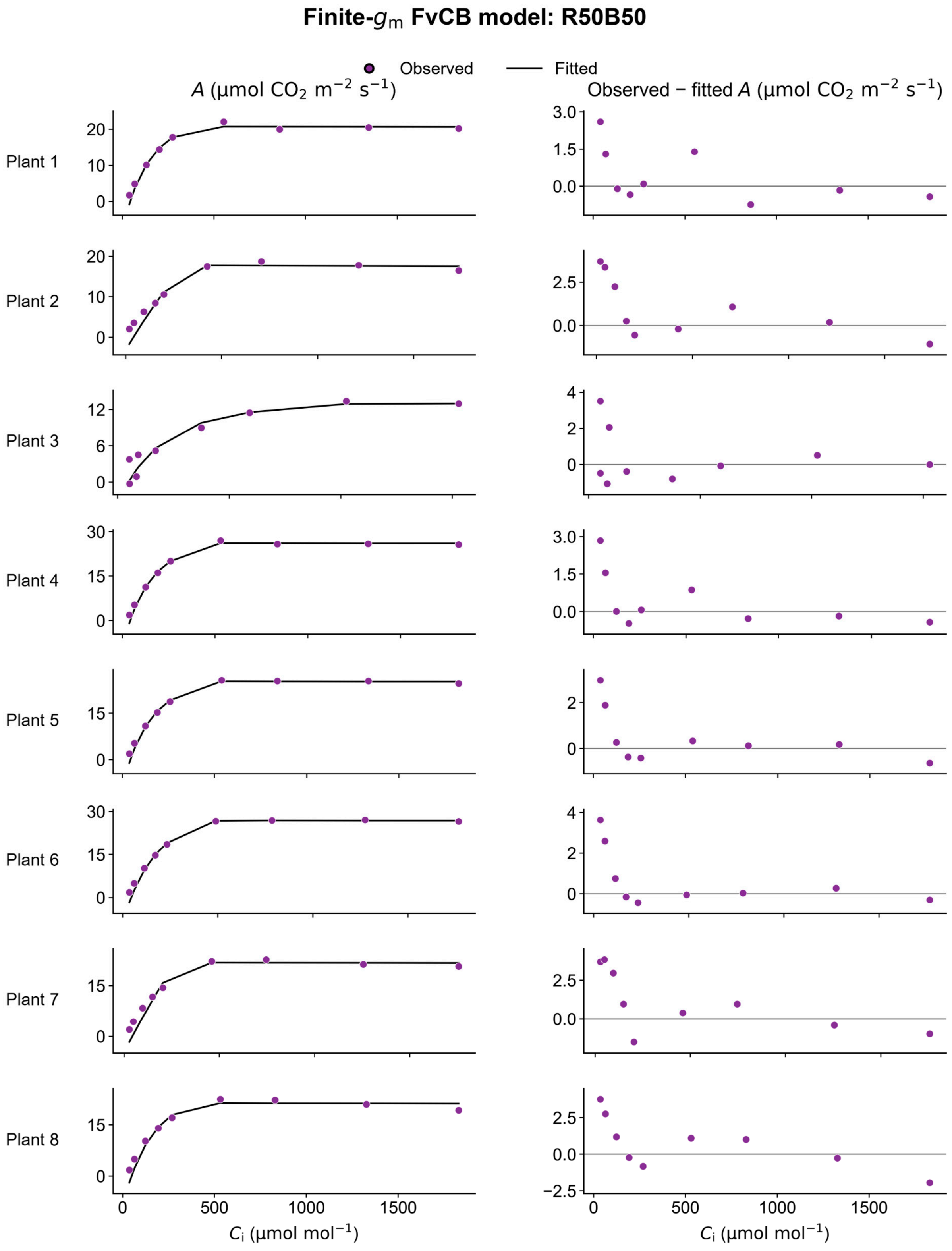

Figure S4. (Continued.) Individual curves fitted with the finite-g_m FvCB model with paired Yin $R_{d,CO2}$: R50B50, plants 1–8. Left: observed net assimilation (coloured points) and fitted values (black lines); right: observed-minus-fit residuals. Each plant has nine retained observations. The four spectral pages show all 32 curves. Axis ranges vary between plants.

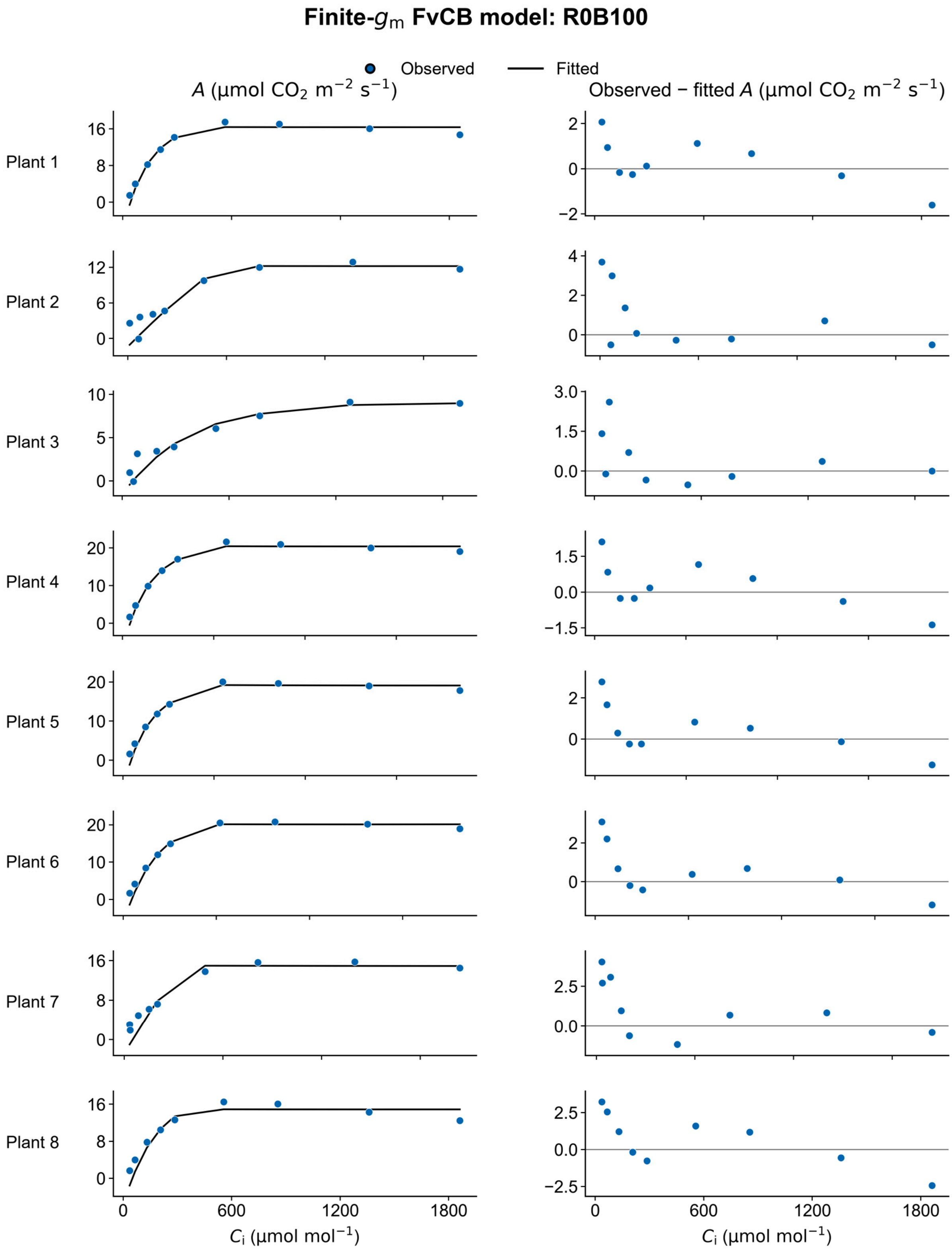

Figure S4. (Continued.) Individual curves fitted with the finite-g_m FvCB model with paired Yin $R_{d,CO2}$: R0B100, plants 1–8. Left: observed net assimilation (coloured points) and fitted values (black lines); right: observed-minus-fit residuals. Each plant has nine retained observations. The four spectral pages show all 32 curves. Axis ranges vary between plants.

Section S9. Individual parameter estimates and conditional uncertainty

Tables S9–S11 distinguish fitted parameters from quantities derived at the measurement irradiance. Each row represents one plant × spectrum curve. Intervals are conditional on the stated model, fixed coefficients and local approximation; they do not include uncertainty in those assumptions. The between-plant confidence intervals are reported separately in Table S5.

Table S9. Individual empirical A–C_i estimates and approximate local 95% intervals.

Spectrum	Plant	$A_{max,CO2}$ Estimate [95% interval]	CE Estimate [95% interval]	Γ_{app} Estimate [95% interval]	RMSE	R^2
R100B0	1	24.32 [23.19, 25.44]	0.1471 [0.1205, 0.1737]	23.31 [10.61, 36.00]	0.660	0.994
R100B0	2	20.35 [19.00, 21.70]	0.1006 [0.0763, 0.1249]	2.17 [−18.13, 22.47]	0.714	0.989
R100B0	3	15.02 [11.07, 18.97]	0.0550 [0.0086, 0.1013]	25.92 [−51.75, 103.58]	1.546	0.920
R100B0	4	29.50 [28.26, 30.74]	0.1686 [0.1415, 0.1957]	22.44 [11.13, 33.75]	0.707	0.995
R100B0	5	28.64 [27.59, 29.69]	0.1253 [0.1091, 0.1414]	17.35 [6.01, 28.70]	0.541	0.997
R100B0	6	31.20 [30.14, 32.26]	0.1685 [0.1470, 0.1901]	22.77 [13.48, 32.07]	0.590	0.997
R100B0	7	25.65 [24.38, 26.93]	0.1667 [0.1342, 0.1992]	23.97 [11.42, 36.52]	0.750	0.993
R100B0	8	25.06 [23.80, 26.32]	0.1630 [0.1304, 0.1955]	25.28 [12.05, 38.50]	0.749	0.992
R90B10	1	23.90 [22.61, 25.20]	0.1580 [0.1239, 0.1922]	27.48 [13.14, 41.82]	0.779	0.991
R90B10	2	20.17 [18.62, 21.72]	0.1120 [0.0790, 0.1451]	7.97 [−14.66, 30.61]	0.862	0.984
R90B10	3	15.54 [12.23, 18.86]	0.0512 [0.0161, 0.0864]	−20.37 [−101.48, 60.74]	1.322	0.939
R90B10	4	28.63 [26.98, 30.29]	0.1916 [0.1476, 0.2355]	29.07 [14.20, 43.95]	0.996	0.990
R90B10	5	28.52 [27.64, 29.40]	0.1655 [0.1455, 0.1856]	27.30 [18.50, 36.10]	0.522	0.997
R90B10	6	31.10 [30.02, 32.18]	0.1714 [0.1488, 0.1940]	24.36 [14.76, 33.96]	0.612	0.997
R90B10	7	25.29 [23.66, 26.91]	0.1701 [0.1267, 0.2135]	24.96 [8.77, 41.15]	0.968	0.988
R90B10	8	24.79 [23.32, 26.26]	0.1638 [0.1250, 0.2026]	26.21 [10.58, 41.84]	0.877	0.989

Table S9. (Continued.) Individual empirical A–C_i estimates and approximate local 95% intervals.

Spectrum	Plant	$A_{max,CO2}$ Estimate [95% interval]	CE Estimate [95% interval]	Γ_{app} Estimate [95% interval]	RMSE	R^2
R50B50	1	20.76 [19.47, 22.04]	0.1479 [0.1106, 0.1852]	28.72 [13.01, 44.43]	0.779	0.988
R50B50	2	18.05 [16.15, 19.94]	0.0880 [0.0536, 0.1223]	1.46 [−31.75, 34.67]	0.998	0.974
R50B50	3	13.51 [10.17, 16.84]	0.0430 [0.0098, 0.0762]†	13.79 [−67.94, 95.52]	1.259	0.932
R50B50	4	26.30 [25.05, 27.55]	0.1604 [0.1303, 0.1905]	27.34 [14.10, 40.57]	0.746	0.993
R50B50	5	25.53 [24.53, 26.53]	0.1479 [0.1247, 0.1711]	25.20 [13.53, 36.87]	0.596	0.995
R50B50	6	27.26 [26.23, 28.30]	0.1512 [0.1290, 0.1734]	23.83 [12.95, 34.71]	0.594	0.996
R50B50	7	21.99 [20.47, 23.51]	0.1305 [0.0943, 0.1668]	12.74 [−6.82, 32.31]	0.868	0.987
R50B50	8	21.32 [19.61, 23.02]	0.1430 [0.0973, 0.1888]	24.58 [3.45, 45.72]	1.005	0.980
R0B100	1	16.44 [15.00, 17.88]	0.1146 [0.0753, 0.1538]	28.01 [6.02, 49.99]	0.830	0.977
R0B100	2	12.87 [9.67, 16.07]	0.0381 [0.0085, 0.0677]†	−15.97 [−112.09, 80.16]	1.264	0.922
R0B100	3	9.42 [7.20, 11.63]	0.0230 [0.0094, 0.0365]†	−8.98 [−94.48, 76.52]	0.714	0.948
R0B100	4	20.52 [19.08, 21.96]	0.1292 [0.0936, 0.1648]	28.75 [9.43, 48.06]	0.842	0.985
R0B100	5	19.24 [17.98, 20.51]	0.1170 [0.0861, 0.1480]	23.98 [4.80, 43.17]	0.754	0.987
R0B100	6	20.33 [19.09, 21.57]	0.1167 [0.0889, 0.1444]	22.26 [4.42, 40.11]	0.719	0.989
R0B100	7	15.66 [14.10, 17.22]	0.0661 [0.0402, 0.0920]	−8.52 [−44.25, 27.22]	0.761	0.980
R0B100	8	14.87 [12.82, 16.91]	0.1046 [0.0479, 0.1613]	21.12 [−14.47, 56.71]	1.146	0.946

Each row represents one complete plant × spectrum curve with nine observations. $A_{max, CO2}$ and RMSE are in μmol CO_2 m^{-2} s^{-1}; CE is in mol m^{-2} s^{-1}; and Γ_{app} is in μmol CO_2 mol^{-1}.

Intervals are approximate local Jacobian intervals under the robust nonlinear fit, as described in Section S5. They quantify conditional fitting uncertainty and do not include uncertainty in the acquisition protocol. They are not adjusted for multiple comparisons.

The implemented bounds for Γ_{app} ranged from −500 to 1000 μmol mol^{-1}. Γ_{app} is below the minimum measured C_i in all 32 curves and is an extrapolated zero crossing.

† The estimate lies within the first or last 1% of its permitted parameter range; the local unconstrained approximation requires particular caution near a constraint. Intervals are not truncated to the fitting bounds.

Table S10. Individual finite-g_m FvCB estimates, approximate local 95% intervals and observed limitation branches.

Spectrum	Plant	V_{cmax} Estimate [95% interval]	J_{max} Estimate [95% interval]	Fitted TPU Estimate [95% interval]	g_m Estimate [95% interval]	Branch counts $A_c/A_j/A_p$
R100B0	1	211.85 [−]‡	166.16 [40.99, 291.34]	8.309 [7.512, 9.106]	0.4034 [−0.8421, 1.6489]	0/5/4
R100B0	2	351.37 [−]‡	356.65 [−8708.11, 9421.40]	6.620 [5.455, 7.784]	0.1063 [−0.3619, 0.5744]	0/5/4
R100B0	3	39.51 [−48.16, 127.18]	72.02 [47.01, 97.03]	5.284 [−]‡	5.0000 [−1992.2092, 2002.2092]†	6/3/0
R100B0	4	264.12 [−]‡	212.09 [130.06, 294.13]	9.983 [8.854, 11.112]	0.4501 [−0.3359, 1.2362]	0/6/3
R100B0	5	234.63 [−]‡	186.94 [104.58, 269.30]	9.588 [8.500, 10.676]	0.2595 [−0.0842, 0.6031]	0/6/3
R100B0	6	261.78 [−]‡	236.33 [130.95, 341.70]	10.801 [9.574, 12.029]	0.4282 [−0.2966, 1.1530]	0/6/3
R100B0	7	261.82 [−]‡	211.13 [−37.01, 459.28]	9.016 [7.986, 10.046]	0.3901 [−0.9267, 1.7070]	0/5/4
R100B0	8	260.32 [−]‡	203.49 [11.48, 395.51]	9.076 [8.039, 10.113]	0.4181 [−1.1189, 1.9550]	0/5/4
R90B10	1	390.09 [−]‡	170.07 [72.92, 267.22]	8.283 [7.553, 9.013]	0.4390 [−0.7742, 1.6521]	1/4/4
R90B10	2	226.21 [−]‡	170.04 [−394.43, 734.51]	6.736 [5.638, 7.834]	0.1695 [−0.4469, 0.7859]	0/5/4
R90B10	3	133.53 [−]‡	89.46 [23.09, 155.82]	5.435 [3.511, 7.358]	0.1000 [−0.2109, 0.4109]	0/7/2
R90B10	4	327.65 [−]‡	207.12 [89.02, 325.21]	9.877 [9.069, 10.684]	0.6696 [−1.5072, 2.8464]	0/5/4
R90B10	5	227.16 [−]‡	190.81 [148.51, 233.10]	9.690 [8.936, 10.444]	0.5103 [−0.2312, 1.2517]	0/6/3
R90B10	6	259.24 [−]‡	233.75 [144.12, 323.38]	10.732 [9.614, 11.849]	0.4528 [−0.2825, 1.1880]	0/6/3
R90B10	7	248.15 [−]‡	206.60 [−28.21, 441.41]	9.026 [7.946, 10.105]	0.4314 [−1.2538, 2.1165]	0/5/4
R90B10	8	369.35 [−]‡	195.17 [31.95, 358.39]	9.052 [8.008, 10.097]	0.4885 [−1.5700, 2.5470]	0/5/4

Each fit used nine observed A–C_i points and four free parameters. The paired Yin-derived $R_{d,CO2}$ offset was fixed. Capacities and g_m refer to the mean measured temperature of each curve. V_{cmax} is in μmol CO_2 m^{-2} s^{-1}; J_{max} is in μmol e^- m^{-2} s^{-1}; fitted TPU is in μmol triose-P m^{-2} s^{-1}; g_m is in mol CO_2 m^{-2} s^{-1}.

The displayed intervals are approximate local Jacobian intervals conditional on $R_{d,CO2}$, the transferred kinetic constants, the assumed J–PPFD relationship and the rapid acquisition protocol. They describe the local numerical approximation and are not independent validations of biochemical or diffusional capacity. Negative lower limits and very broad intervals express the breadth of that approximation; they do not make negative parameters admissible in the bounded fit.

Table S10. (Continued.) Individual finite-g_m FvCB estimates, approximate local 95% intervals and observed limitation branches.

Spectrum	Plant	V_{cmax} Estimate [95% interval]	J_{max} Estimate [95% interval]	Fitted TPU Estimate [95% interval]	g_m Estimate [95% interval]	Branch counts $A_c/A_j/A_p$
R50B50	1	202.68 [—]‡	142.17 [73.52, 210.81]	7.202 [6.531, 7.874]	0.4974 [−1.2199, 2.2147]	0/5/4
R50B50	2	265.54 [—]‡	214.37 [−2914.01, 3342.75]	5.972 [4.861, 7.083]	0.1006 [−0.4247, 0.6259]	0/5/4
R50B50	3	161.20 [—]‡	69.03 [46.48, 91.57]	4.734 [3.024, 6.445]	0.1288 [−0.2542, 0.5119]	0/8/1
R50B50	4	219.14 [—]‡	171.08 [86.94, 255.22]	9.011 [8.347, 9.675]	0.5643 [−1.1222, 2.2507]	0/5/4
R50B50	5	312.03 [—]‡	165.77 [73.01, 258.52]	8.819 [8.010, 9.628]	0.5111 [−1.1333, 2.1556]	0/5/4
R50B50	6	233.20 [—]‡	190.86 [127.47, 254.25]	9.476 [8.460, 10.493]	0.4154 [−0.3053, 1.1361]	0/6/3
R50B50	7	440.33 [—]‡	1000.00 [-8.76×10^4, 8.96×10^4]†	7.788 [6.525, 9.050]	0.1304 [−0.4760, 0.7367]	0/5/4
R50B50	8	240.98 [—]‡	163.87 [16.05, 311.69]	7.861 [6.768, 8.955]	0.4113 [−1.5786, 2.4012]	0/5/4
R0B100	1	139.90 [—]‡	103.20 [53.11, 153.30]	5.635 [4.987, 6.284]	0.4367 [−1.4940, 2.3675]	0/5/4
R0B100	2	166.42 [—]‡	109.31 [−567.05, 785.67]	4.183 [3.049, 5.316]	0.0398 [−0.0774, 0.1571]†	0/6/3
R0B100	3	200.27 [—]‡	45.96 [29.39, 62.53]	3.246 [2.043, 4.449]	0.0448 [−0.0418, 0.1314]†	0/8/1
R0B100	4	212.24 [—]‡	124.20 [77.22, 171.18]	7.015 [6.414, 7.616]	0.4946 [−1.1409, 2.1300]	0/5/4
R0B100	5	170.49 [—]‡	118.02 [45.71, 190.34]	6.743 [6.029, 7.457]	0.5062 [−2.1732, 3.1855]	0/5/4
R0B100	6	184.85 [—]‡	137.67 [16.59, 258.74]	7.149 [6.343, 7.956]	0.2938 [−0.7372, 1.3249]	0/5/4
R0B100	7	510.31 [—]‡	1000.00 [-2.45×10^5, 2.47×10^5]†	5.437 [4.245, 6.629]	0.0723 [−0.3615, 0.5061]	0/5/4
R0B100	8	223.59 [—]‡	117.62 [−19.43, 254.67]	5.653 [4.490, 6.816]	0.2306 [−1.0237, 1.4850]	0/5/4

† At or near a parameter bound (within the first or last 1% of the permitted range). Ordinary unconstrained interval coverage is not assured near a constraint. For R100B0 plant 3, g_m reached its upper bound; J_{max} reached its upper bound for R50B50 plant 7 and R0B100 plant 7.

‡ No informative local interval: a zero-width or numerically inactive direction of the local covariance approximation is displayed as —, rather than as zero uncertainty. The fitted point estimate is retained.

Branch counts assign each observed point to the smallest modelled net rate, A_c, A_j or A_p; the counts sum to nine. Thirty curves had no A_c-limited observation points. These counts explain the parameter-specific resolving power and should be read together with the coupled-parameter sensitivity results in Section S5.

The fitted TPU in this table is a free FvCB parameter and differs from TPU_{op}, the operational high-C_i proxy calculated from observed assimilation.

Table S11. Modelled electron transport at a PPFD of 1000 and ambient finite-g_m descriptors for individual curves.

Spectrum	Plant	T_{ref} (°C)	J_{max}	J at PPFD 1000	Ambient g_m	Ambient C_c
R100B0	1	26.63	166.16	133.83	0.4048	199.43
R100B0	2	26.67	356.65	209.91	0.1062	82.01
R100B0	3	26.93	72.02	66.37	4.9904†	169.35
R100B0	4	26.70	212.09	158.66	0.4489	176.67
R100B0	5	26.83	186.94	145.68	0.2587	159.29
R100B0	6	26.82	236.33	169.84	0.4297	164.99
R100B0	7	26.85	211.13	158.19	0.3891	182.57
R100B0	8	26.65	203.49	154.39	0.4172	210.47
R90B10	1	26.55	170.07	136.14	0.4390	221.25
R90B10	2	26.67	170.04	136.12	0.1696	126.10
R90B10	3	27.11	89.46	80.58	0.0996	104.65
R90B10	4	26.41	207.12	156.21	0.6714	222.58
R90B10	5	26.53	190.81	147.77	0.5107	217.70
R90B10	6	26.70	233.75	168.71	0.4524	176.46
R90B10	7	26.78	206.60	155.95	0.4319	188.82
R90B10	8	26.68	195.17	150.09	0.4897	223.01
R50B50	1	26.78	142.17	118.80	0.4994	238.69
R50B50	2	27.16	214.37	159.77	0.1007	88.10
R50B50	3	27.46	69.03	63.85	0.1279	125.51
R50B50	4	26.58	171.08	136.73	0.5670	223.91
R50B50	5	26.68	165.77	133.59	0.5146	220.50
R50B50	6	26.84	190.86	147.80	0.4173	186.83
R50B50	7	27.12	1000.00	270.03	0.1309	82.06
R50B50	8	27.01	163.87	132.46	0.4124	225.66
R0B100	1	27.22	103.20	91.24	0.4374	252.32
R0B100	2	27.30	109.31	95.82	0.0397	71.06
R0B100	3	27.48	45.96	43.72	0.0445	145.34
R0B100	4	26.72	124.20	106.58	0.4933	267.39
R0B100	5	26.97	118.02	102.19	0.5089	225.43
R0B100	6	27.05	137.67	115.82	0.2941	201.10
R0B100	7	27.40	1000.00	270.03	0.0726	59.63
R0B100	8	27.24	117.62	101.90	0.2304	226.86

The values are derived from the finite-g_m fit used for the main manuscript descriptors. T_{ref} is the curve-mean measured leaf temperature. J_{max} and J at a PPFD of 1000 are in μmol e⁻ m^{-2} s^{-1}; ambient g_m is in mol CO_2 m^{-2} s^{-1}; and ambient C_c is in μmol CO_2 mol^{-1}.

J at a PPFD of 1000 is evaluated at T_{ref} using the nonrectangular J–PPFD hyperbola with $\alpha_J = 0.30$ and $\theta_J = 0.70$. Thus, J_{max} is the fitted asymptote, whereas J at a PPFD of 1000 is the electron transport rate predicted at that irradiance. Neither quantity is fluorescence-derived apparent ETR.

Ambient descriptors use the retained observation with measured C_a nearest 399.01 μmol mol^{-1}. The conductance is adjusted to the observation temperature; $C_c = C_i - A_{model}/g_m$ uses fitted net assimilation. No interpolation to the nominal C_a 400 was applied.

† The R100B0 plant 3 conductance is shown here as an individual model result. The group g_m summary uses the seven non-boundary estimates because plant 3's fitted reference temperature g_m reached its upper bound. Its associated C_c remains in the original eight-plant C_c summary.

The individual conditional intervals for the free-fit parameters are given in Table S10. Ambient C_c is a derived model quantity; an independent local confidence interval for it was not calculated.

Section S10. Comparison of the 16-descriptor and 15-descriptor PCA

The primary PCA integrates the 16 reported descriptors. The complementary analysis uses the 15 Figure 3 descriptors, the same 32 plant × spectrum curve sets, scaling and observed-cell NIPALS procedure. The single missing g_m value remains missing. Both analyses retain four components; their explained variance and subspace agreement are summarised in Table S12. This comparison assesses whether adding TPU_{op} determines the broad multivariate structure.

Table S12. Sensitivity of the response curve PCA to descriptor selection. The primary 16-variable PCA includes TPU_{op} alongside the 15 Figure 3 descriptors; the complementary analysis uses those 15 descriptors. Both use the same 32 plant × spectrum curve sets and retain four components. Values are percentages of variance in their respective scaled matrices; component axes are fitted separately.

Component	Primary variance (%)	15-descriptor variance (%)	Primary cumulative (%)	15-descriptor cumulative (%)
PC1	46.4053	47.6527	46.4053	47.6527
PC2	18.6865	18.4696	65.0918	66.1223
PC3	15.8495	15.5343	80.9413	81.6566
PC4	9.1396	8.1268	90.0809	89.7834

Both analyses centre and scale observed cells to unit sample variance, retain the single missing g_m value without imputation and use observed-cell NIPALS. The canonical correlations between the retained four-dimensional score subspaces are 1.00000, 0.99999, 0.99975 and 0.98995. These are subspace correlations, not correlations between the individual PC rows. The component signs are arbitrary.

Section S11. Fitted-coefficient sensitivity and multivariate influence

The primary finite-g_m coefficients were re-evaluated at the measured C_i, PPFD, temperature and pressure, without changing the original observations, equations or estimates. This reproduced the 30 curves with no model-assigned A_c-limited observation. The three FvCB limitation expressions describe potential controlling regimes; their inclusion in the model does not require each measured curve to contain a separately identifiable segment for every regime. Of the seven points assigned to A_c, six occurred in R100B0 plant 3, with g_m at its upper bound; the remaining point in R90B10 plant 1 was a numerical A_c–A_j tie near $C_c = \Gamma^*$ and provided negligible V_{cmax} sensitivity. A separate scalar solution of $A = \min\{A_c, A_j, A_p\}$, with $C_c = C_i - A/g_m$, reproduced all pointwise predictions and assignments. These model assignments therefore do not demonstrate the physiological absence of Rubisco limitation. The parameter-specific diagnostics in Section S5.7 determine the scope of interpretation.

We summarised the primary J_{max} estimates and the existing assumption-sensitivity fits separately (Table S13). The primary fit used the five starting vectors described in Section S5.6. The nominal-setting sensitivity fit and its assumption variants used three starting vectors: the primary [V_{cmax}, J_{max}, TPU, g_m] vector, [100, 150, max(A)/3, 0.20] and [200, 300, max(A)/3, 0.10], clipped within the relevant bounds with a margin of 10^{-6}. These sensitivity fits are distinct from the primary solution. The upper-bound and α_J perturbations use their nominal-setting fit as the control when quantifying the changes previously reported in Section S5.7. Table S13 instead gives the largest RMSE difference from the primary solution for every scenario, making the distinction explicit. These scenarios preserve the observed responses and classical equations; they test the dependence of the fitted coefficients on numerical and fixed-parameter choices. Small changes in residual error remain compatible with that dependence.

The two primary estimates at J_{max} = 1000 μmol e^- m^{-2} s^{-1} both belonged to plant 7, under R50B50 and R0B100. We compared the complete 32-curve dataset with two 31-

curve sets, a 30-curve set and a balanced 28-curve set, as specified below Table S13. All original observations and primary results are retained. In the 30-curve comparison, the R50B50 mean ± SEM was 159.59 ± 17.37 versus 264.64 ± 106.12 in the primary analysis; the R0B100 mean was 108.00 ± 11.14 versus 219.50 ± 111.92 μmol e^- m^{-2} s^{-1}. The balanced 28-curve comparison gave $F(3,18) = 6.6375$, $p = 0.0033$, versus $F(3,21) = 0.2241$, $p = 0.8786$ for the primary plant-blocked HC3 test. Thus, both the coefficient magnitudes and the nominal inference were sensitive to these estimates. The comparison does not replace the primary test or select a preferred biological conclusion. Between-plant SEMs describe variation among conditional point estimates and do not resolve within-curve identification.

For PCA, each comparison matrix used the 16 original descriptors, re-centred and rescaled, with the missing g_m cell still unavailable and NIPALS applied to observed cells. Retention continued to use eigenvalues ≥ 1. Agreement was assessed through canonical correlations between score spaces on common curve rows and principal angles between the four-dimensional loading subspaces. The 30-curve analysis retained four components, which explained 90.3158% of the variance, compared with 90.0809% in the primary analysis. The smallest score canonical correlation was 0.994393, but the largest loading-subspace angle was 18.3687°. The balanced 28-curve analysis retained four components, which explained 90.2200% of the variance, with corresponding values of 0.993909 and 18.3193° (Table S14; Figure S5). The overall score configuration therefore remained close while the contribution of the fitted coefficients changed appreciably. No individual component is interpreted as an independently established biochemical mechanism.

A further sensitivity retained nine operational light-response and empirical descriptors: $A_{p,max}$, A_{max}, α, $R_{d,light}$, light compensation point, $PPFD_{75}$, g_{sw} at PPFD 400, empirical $A_{max,CO2}$ and paired Yin $R_{d,CO2}$. This matrix contained all 32 curve sets and these nine descriptors only. Three components explained 93.9555% of the nine-variable variance. Their canonical correlations with the primary four-dimensional score space were 0.998988, 0.992414 and 0.932736. Because the feature sets and retained dimensionalities differ, these values quantify overlap and do not validate the additional model-dependent dimensions. The original 16-descriptor PCA remains a descriptive summary, interpreted alongside these checks and the direct response-curve comparisons.

Table S13. Sensitivity and influence of fitted J_{max} estimates.

**Analysis**	**R100B0**	**R90B10**	**R50B50**	**R0B100**	**Conditional spectrum *p***	**Largest |ΔRMSE|**
Primary estimates	8: 205.60 ± 27.90	8: 182.87 ± 15.24	8: 264.64 ± 106.12	8: 219.50 ± 111.92	0.8786	0.00000
Nominal settings (three starts)	8: 186.09 ± 17.70	8: 183.01 ± 15.23	8: 272.30 ± 105.88	8: 329.80 ± 146.56	0.6974	0.23727
J_{max} upper bound 2000	8: 186.02 ± 17.69	8: 183.67 ± 15.17	8: 393.89 ± 230.12	8: 579.80 ± 310.06	0.5637	0.23727
$\alpha_J = 0.20$	8: 281.26 ± 39.30	8: 274.13 ± 35.02	8: 397.51 ± 133.19	8: 232.97 ± 110.23	0.7391	0.24303
$\alpha_J = 0.40$	8: 166.65 ± 14.84	8: 168.02 ± 12.61	8: 213.73 ± 63.88	8: 326.29 ± 147.28	0.7221	0.23723
31 curves (R50B50 $n = 7$)	8: 205.60 ± 27.90	8: 182.87 ± 15.24	7: 159.59 ± 17.37	8: 219.50 ± 111.92	—	—
31 curves (R0B100 $n = 7$)	8: 205.60 ± 27.90	8: 182.87 ± 15.24	8: 264.64 ± 106.12	7: 108.00 ± 11.14	—	—
30 curves (two spectra $n = 7$)	8: 205.60 ± 27.90	8: 182.87 ± 15.24	7: 159.59 ± 17.37	7: 108.00 ± 11.14	—	—

Analysis	R100B0	R90B10	R50B50	R0B100	Conditional spectrum p	Largest ∣ΔRMSE∣
28 curves (plants 1–6 and 8)	7: 204.81 ± 32.20	7: 179.49 ± 17.16	7: 159.59 ± 17.37	7: 108.00 ± 11.14	0.0033	—

Group cells show n: mean ± SEM for J_{max} in μmol e^- m^{-2} s^{-1}. Primary estimates are unchanged. The primary fitting procedure used five starting vectors: an algorithmic initialisation and four multiplicative perturbations. The previously calculated assumption sensitivities used three deterministic starting vectors: the primary coefficient vector, [100, 150, maximum observed A/3, 0.20] and [200, 300, maximum observed A/3, 0.10] for [V_{cmax}, J_{max}, TPU, g_m], clipped inside each scenario's bounds.

The first five rows compare primary estimates with previously calculated refits under the same classical finite-g_m equations: nominal settings, upper bound 2000, α_J = 0.20 and α_J = 0.40. Respiration offsets, θ_J = 0.70 and the retained observations are unchanged in these four refit scenarios. The nominal three-start refit is a sensitivity control and does not replace the five-start primary fit. ∣ΔRMSE∣ in this table is measured relative to the primary estimates, in μmol CO_2 m^{-2} s^{-1}; the earlier maximal changes of 0.00484 and 0.0409 in Section S5 compare changed assumptions with the three-start nominal control.

The final four rows compare defined curve sets. Each 31-curve set contains plants 1–6 and 8 for the named spectrum and all eight plants for the other spectra. The 30-curve set contains plants 1–6 and 8 for R50B50 and R0B100, and all eight for R100B0 and R90B10. The balanced 28-curve set contains plants 1–6 and 8 for every spectrum. The two upper-bound J_{max} estimates belong to plant 7 under R50B50 and R0B100. All 32 curves remain in the primary analysis; these comparisons do not diagnose plant 7 as biologically invalid.

Spectrum p values are plant-blocked HC3 comparisons of fitted point estimates. They are conditional on the parameterisation and do not include within-curve parameter uncertainty. Sensitivity tests are shown only for the balanced 28-curve set; the 31-curve comparisons are descriptive checks.

SEM and between-plant intervals describe dispersion among fitted estimates. Stability of an omnibus p value does not demonstrate that a coefficient is independently identified or that physiological capacities are equivalent.

Table S14. Influence checks and operational-descriptor sensitivity of the response-curve PCA.

Analysis	Curves	Variables	Retained PCs	Retained variance (%)	Smallest score canonical correlation	Largest loading angle (°)
All 32 curves	32	16	4	90.08	1.00000	0.00
31 curves (R50B50 n = 7)	31	16	4	89.78	0.99860	4.00
31 curves (R0B100 n = 7)	31	16	4	89.62	0.99908	7.46
30 curves (two spectra n = 7)	30	16	4	90.32	0.99439	18.37
28 curves (plants 1–6 and 8)	28	16	4	90.22	0.99391	18.32
Nine operational light/empirical descriptors	32	9	3	93.96	0.93274	—

The primary PCA uses the unchanged 16-descriptor matrix of 32 plant × spectrum curves. Each omission check uses the same variables, observed-cell NIPALS method and retention rule, with centring and scaling recalculated from its retained observations. The single missing g_m cell remains missing.

For the 16-variable comparisons, agreement is assessed between the first four loading dimensions and between four-dimensional score spaces on common curve rows. Retention according to eigenvalues ≥ 1 is reported separately; a comparison of four dimensions does not force four components to be retained.

The nine-variable comparison retains $A_{p,max}$, A_{max}, α, $R_{d,light}$, LCP, $PPFD_{75}$, g_{sw} at PPFD 400, empirical $A_{max,CO2}$ and paired Yin $R_{d,CO2}$. This secondary sensitivity contains these nine variables only, with all 32 curve sets; the primary 16-variable matrix is preserved.

The nine-variable analysis has three retained dimensions, compared with the primary four-dimensional space. Its three canonical correlations describe overlap between spaces of different dimensions. No loading angle is reported across different feature sets; close overlap does not validate the additional model-derived dimensions.

PCA describes covariance among curve-level descriptors. Neither the original nor sensitivity ordinations independently identify biochemical mechanisms. Component signs are arbitrary.

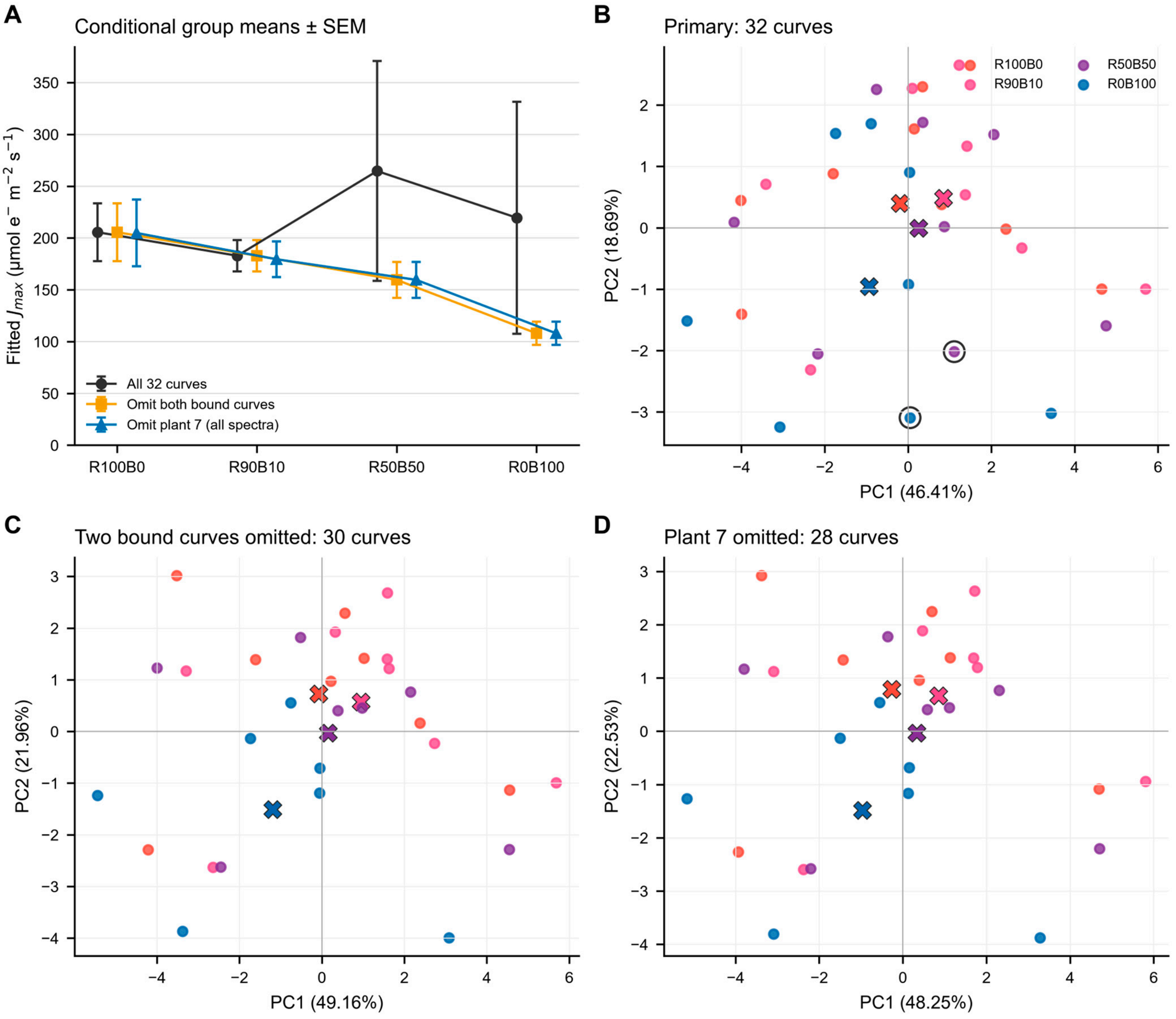

Figure S5. Influence of upper-bound J_{max} estimates on group summaries and response-curve PCA. (A) Mean ± SEM estimates for the primary 32-curve, complementary 30-curve and balanced 28-curve sets defined below Table S13. (B) Primary 16-descriptor PCA; circles outline the two upper-bound curve sets. (C) The 30-curve PCA. (D) The 28-curve PCA comprising plants 1–6 and 8 across all spectra. Crosses show spectral centroids. Each sensitivity uses its own centring and scaling; displayed axes are its own PC1 and PC2. Full four-dimensional comparisons are reported in Table S14. These are influence diagnostics; all observations and primary estimates are retained.

Section S12. Stability classifications and observed-response sensitivity

Recorded analyser classifications were linked to all 608 response-curve observations by curve identifier and within-curve point index: 32 ten-point light curves and 32 nine-point CO_2 curves from eight plants under four spectral recipes. All observations were retained in the primary analyses. The complementary sensitivity compared the complete profiles with two predefined subsets: points classified as 1/2 or 2/2, and points classified as 2/2. Classifications, point counts and response-axis coverage were summarised by spectrum and programmed step.

Direct observed assimilation was compared at all 10 nominal PPFD steps and all nine nominal CO_2 steps using the available within-plant spectral pairs. These descriptive

comparisons report the number of complete pairs, with no substitution of observations. For each subset, the common integration interval was the intersection of the observed domains across all 32 curves, requiring at least two observations per curve. The complete profile and its subset were integrated over this same interval using piecewise-linear interpolation within observed bounds, without extrapolation, and divided by the interval width. All six spectral contrasts used eight within-plant pairs and unadjusted descriptive 95% paired t intervals. The nonlinear models continued to use the complete curves; no nonlinear fits were made to these subsets. Because classifications depend on the measured signals, the comparisons describe sensitivity within the represented domains and do not establish physiological equilibrium.

The distribution of classifications varied markedly across programmed steps (Table S15). In the CO_2 protocol, 31 of 32 observations at the initial C_a 400 step were classified as 2/2, compared with none at C_a 300, 200, 1500 or 2000. There were 22 such observations at C_a 50 and eight at C_a 700. Across recipes, 2/2 counts ranged from 12 to 19 of 72 CO_2 observations and from 55 to 64 of 80 light-response observations.

The 2/2 subset contained 1–4 observations per CO_2 curve, with a median of two; none met the original six-point minimum. Eight curves contributed one observation, so this subset had no common integration domain. The 1/2-or-2/2 subset contained 4–9 points per curve, with a median of seven, and 28 of 32 curves met the point minimum. Its common C_i domain was 57.88–530.50 μmol mol^{-1}, corresponding to 35.7% of the complete-profile common-domain width (Table S16). This sensitivity therefore addresses the lower and intermediate C_i region; the high-C_i observations remain part of the complete-profile analysis.

The light-response subsets covered broader domains. The 1/2-or-2/2 subset contained 298 points over a common PPFD domain of 30.00–999.95 μmol photons m^{-2} s^{-1}; the 2/2 subset contained 242 points over 60.01–800.23. The mean spectral ordering was preserved in the complete-profile and subset comparisons on identical domains (Table S17), with lower precision for some contrasts. For the 2/2 common domain, the R90B10–R50B50 difference was 0.819 [0.228, 1.410] for complete profiles and 0.455 [−0.189, 1.099] for the subset; the corresponding R90B10–R0B100 differences were 1.987 [0.966, 3.007] and 1.781 [0.748, 2.814] μmol CO_2 m^{-2} s^{-1}. These are descriptive, unadjusted intervals.

Within the common CO_2 domain represented by the 1/2-or-2/2 subset, the mean spectral ordering was also preserved. The R100B0–R0B100 difference was 6.265 [4.847, 7.683] for complete profiles and 6.485 [4.818, 8.151] μmol CO_2 m^{-2} s^{-1} for the subset. At C_a 2000, the 2/2 subset provided no paired contrast; the 1/2-or-2/2 subset provided two or three pairs for comparisons with R0B100. Their mean differences were positive, but these small subsets do not provide an independent assessment of the complete high-C_i response. The primary curve comparisons and model estimates continue to use all measured points.

Table S15. Recorded analyser stability classifications by spectrum and programmed response-curve step. Each cell gives the numbers of observations classified as 2/2; 1/2; 0/2, in that order, across the eight plants. Every cell sums to eight. Steps follow the programmed within-curve sequence.

Protocol	Step: target	R100B0	R90B10	R50B50	R0B100
A–PPFD	1: 1000	8; 0; 0	7; 1; 0	8; 0; 0	8; 0; 0
A–PPFD	2: 800	8; 0; 0	8; 0; 0	7; 1; 0	8; 0; 0
A–PPFD	3: 600	8; 0; 0	7; 1; 0	7; 1; 0	6; 2; 0
A–PPFD	4: 400	4; 4; 0	3; 4; 1	5; 3; 0	6; 1; 1
A–PPFD	5: 250	3; 3; 2	1; 2; 5	5; 2; 1	3; 1; 4
A–PPFD	6: 150	6; 2; 0	3; 2; 3	4; 3; 1	5; 3; 0

Protocol	Step: target	R100B0	R90B10	R50B50	R0B100
A–PPFD	7: 100	7; 1; 0	7; 0; 1	7; 1; 0	6; 2; 0
A–PPFD	8: 60	8; 0; 0	8; 0; 0	8; 0; 0	6; 1; 1
A–PPFD	9: 30	6; 2; 0	6; 2; 0	8; 0; 0	8; 0; 0
A–PPFD	10: 0.05	3; 5; 0	5; 2; 1	5; 3; 0	6; 1; 1
A–C_i	1: 400	7; 1; 0	8; 0; 0	8; 0; 0	8; 0; 0
A–C_i	2: 300	0; 5; 3	0; 6; 2	0; 7; 1	0; 6; 2
A–C_i	3: 200	0; 7; 1	0; 6; 2	0; 3; 5	0; 6; 2
A–C_i	4: 100	1; 4; 3	0; 4; 4	0; 6; 2	0; 4; 4
A–C_i	5: 50	6; 2; 0	6; 2; 0	6; 2; 0	4; 3; 1
A–C_i	6: 700	4; 4; 0	2; 5; 1	2; 3; 3	0; 7; 1
A–C_i	7: 1000	1; 6; 1	0; 5; 3	0; 6; 2	0; 7; 1
A–C_i	8: 1500	0; 6; 2	0; 4; 4	0; 6; 2	0; 4; 4
A–C_i	9: 2000	0; 6; 2	0; 4; 4	0; 5; 3	0; 4; 4

Targets are nominal PPFD in μmol photons m^{-2} s^{-1} for *A*–PPFD and nominal reference CO_2 in μmol mol^{-1} for A–C_i. Measured PPFD and C_i are used in the common-domain calculations.

The recorded classifications were retained unchanged. *A* classification of 2/2 means both configured analyser criteria were met; it does not establish full physiological equilibration.

Table S16. Observation and domain coverage of the complete profiles and predefined classification subsets. All original observations are retained in the primary analysis. The common domain is the intersection of the observed response-axis ranges across all 32 curves, requiring at least two observations per curve and a positive-width intersection.

Protocol	Subset	Retained points	Per-curve range (median)	Curves meeting point minimum	Common observed domain
A–PPFD	All observations	320	10–10 (10.0)	32/32	0.05–999.95
A–PPFD	1/2 or 2/2	298	6–10 (10.0)	32/32	30.00–999.95
A–PPFD	2/2	242	3–10 (8.0)	30/32	60.01–800.23
A–C_i	All observations	288	9–9 (9.0)	32/32	57.88–1380.41
A–C_i	1/2 or 2/2	219	4–9 (7.0)	28/32	57.88–530.50
A–C_i	2/2	63	1–4 (2.0)	0/32	Unavailable

The original software minimum was five points for the light model and six for A–C_i. Meeting this point count does not establish parameter identification. Nonlinear model estimates were obtained from complete curves; no model was refitted to the subsets.

Domain units are PPFD (μmol photons m^{-2} s^{-1}) for A–PPFD and measured C_i (μmol mol^{-1}) for A–C_i. Eight A–C_i curves have one 2/2 point, so that subset has no common integration domain. No observation at C_a 1500 or 2000 has a 2/2 classification.

The complete A–C_i profiles span a common domain of 57.88–1380.41 μmol mol^{-1}; the 1/2-or-2/2 subset spans 57.88–530.50 μmol mol^{-1}, representing 35.7% of that width. The high-C_i plateau is represented in the complete profiles.

Table S17. Within-plant contrasts of normalised observed area on identical common domains. Each contrast uses eight plant pairs. Complete profiles and their classification subsets are integrated on the same domains specified in Table S16, holding the integration interval constant for each comparison. Differences are in μmol CO_2 m^{-2} s^{-1}.

Protocol	Subset	Contrast	All-point difference [95% interval]	Subset difference [95% interval]
A–PPFD	1/2 or 2/2	R100B0 − R90B10	−1.352 [−2.950, 0.245]	−1.223 [−2.967, 0.521]
A–PPFD	1/2 or 2/2	R100B0 − R50B50	−0.467 [−2.430, 1.496]	−0.468 [−2.428, 1.491]
A–PPFD	1/2 or 2/2	R100B0 − R0B100	0.801 [−1.145, 2.748]	0.860 [−1.119, 2.840]
A–PPFD	1/2 or 2/2	R90B10 − R50B50	0.885 [0.159, 1.612]	0.755 [0.093, 1.417]
A–PPFD	1/2 or 2/2	R90B10 − R0B100	2.153 [0.971, 3.336]	2.083 [0.779, 3.387]
A–PPFD	1/2 or 2/2	R50B50 − R0B100	1.268 [0.193, 2.343]	1.329 [0.245, 2.412]
A–PPFD	2/2	R100B0 − R90B10	−1.270 [−2.781, 0.242]	−0.963 [−2.629, 0.703]
A–PPFD	2/2	R100B0 − R50B50	−0.450 [−2.220, 1.319]	−0.508 [−2.238, 1.223]
A–PPFD	2/2	R100B0 − R0B100	0.717 [−1.116, 2.550]	0.818 [−0.759, 2.396]
A–PPFD	2/2	R90B10 − R50B50	0.819 [0.228, 1.410]	0.455 [−0.189, 1.099]
A–PPFD	2/2	R90B10 − R0B100	1.987 [0.966, 3.007]	1.781 [0.748, 2.814]
A–PPFD	2/2	R50B50 − R0B100	1.167 [0.247, 2.088]	1.326 [0.723, 1.929]
A–C_i	1/2 or 2/2	R100B0 − R90B10	−0.466 [−0.997, 0.065]	−0.170 [−1.121, 0.781]
A–C_i	1/2 or 2/2	R100B0 − R50B50	1.895 [1.189, 2.602]	2.277 [1.334, 3.220]
A–C_i	1/2 or 2/2	R100B0 − R0B100	6.265 [4.847, 7.683]	6.485 [4.818, 8.151]
A–C_i	1/2 or 2/2	R90B10 − R50B50	2.361 [2.088, 2.634]	2.447 [2.222, 2.672]
A–C_i	1/2 or 2/2	R90B10 − R0B100	6.731 [5.624, 7.837]	6.655 [5.373, 7.936]
A–C_i	1/2 or 2/2	R50B50 − R0B100	4.369 [3.446, 5.293]	4.208 [2.809, 5.606]

Normalised areas use piecewise-linear integration over observed bounds, divided by the common-domain width; no endpoint is extrapolated.

Intervals are unadjusted descriptive paired t intervals (n = 8), with all six contrasts reported. Signal-based classification does not define random or necessarily less biased subsets.

The 2/2 A–C_i subset has no common integration domain. These subset comparisons do not independently assess the high-C_i plateau or biochemical TPU capacity.
